# Active Media Perfusion in Bioprinted Highly Concentrated Collagen Bioink Enhances the Viability of Cell Culture and Substrate Remodeling

**DOI:** 10.3390/gels10050316

**Published:** 2024-05-05

**Authors:** Denisa Kanokova, Roman Matejka, Margit Zaloudkova, Jan Zigmond, Monika Supova, Jana Matejkova

**Affiliations:** 1 Department of Biomedical Technology, Faculty of Biomedical Engineering, Czech Technical University in Prague, Sitna 3105, 272 01 Kladno, Czech Republic; kanokden@fbmi.cvut.cz (D.K.); roman.matejka@cvut.cz (R.M.); jan.zigmond@fbmi.cvut.cz (J.Z.); 2 Department of Composites and Carbon Materials, Institute of Rock Structure and Mechanics, Czech Academy of Sciences, 182 09 Prague, Czech Republic; zaloudkova@irsm.cas.cz (M.Z.); supova@irsm.cas.cz (M.S.)

**Keywords:** collagen, active perfusion, bioprinting, bioinks, high-concentrated polymer hydrogels

## Abstract

The bioprinting of high-concentrated collagen bioinks is a promising technology for tissue engineering and regenerative medicine. Collagen is a widely used biomaterial for bioprinting because of its natural abundance in the extracellular matrix of many tissues and its biocompatibility. High-concentrated collagen hydrogels have shown great potential in tissue engineering due to their favorable mechanical and structural properties. However, achieving high cell proliferation rates within these hydrogels remains a challenge. In static cultivation, the volume of the culture medium is changed once every few days. Thus, perfect perfusion is not achieved due to the relative increase in metabolic concentration and no medium flow. Therefore, in our work, we developed a culture system in which printed collagen bioinks (collagen concentration in hydrogels of 20 and 30 mg/mL with a final concentration of 10 and 15 mg/mL in bioink) where samples flow freely in the culture medium, thus enhancing the elimination of nutrients and metabolites of cells. Cell viability, morphology, and metabolic activity (MTT tests) were analyzed on collagen hydrogels with a collagen concentration of 20 and 30 mg/mL in static culture groups without medium exchange and with active medium perfusion; the influence of pure growth culture medium and smooth muscle cells differentiation medium was next investigated. Collagen isolated from porcine skins was used; every batch was titrated to optimize the pH of the resulting collagen to minimize the difference in production batches and, therefore, the results. Active medium perfusion significantly improved cell viability and activity in the high-concentrated gel, which, to date, is the most limiting factor for using these hydrogels. In addition, based on SEM images and geometry analysis, the cells remodel collagen material to their extracellular matrix.

## 1. Introduction

Bioprinting combines principles, protocols, and fabrication techniques from various disciplines, such as biomedicine, engineering, electronics, material science, and cell biology [1]. One of the aims of this field is to develop three-dimensional (3D) native-like heterogeneous complexes that incorporate cells. The bioprinting of polymers enables the creation of complex structures that possess characteristics similar to biological mass or extracellular matter, thereby increasing the possibility of tissue generation. Bioinks are cell-laden polymers deposited in a hydrogel to create a controlled pattern to fabricate living tissues and organs [2]. While the use of bioinks is hindered by the need to use a delivery medium for cells, appropriate bioinks provide cell-binding sites that are desirable for cell attachment, spreading, growth, and differentiation [3]. A wide range of natural or synthetic biomaterials are used in tissue engineering, but only a few possess the necessary features to create an ideal bioink. Polymers that are suitable for bioprinting should have specific characteristics such as bioprintability, high mechanical strength and stability, insolubility in cell culture medium, an appropriate biodegradability rate for the regenerating tissue, an absence of toxicity and immunogenicity, and the ability to facilitate cell adhesion [2]. Proper mechanical, rheological, chemical, and biological characteristics are also required [4]. Natural polymers obtained through physical or chemical methods are preferred over synthetic materials due to their excellent biological compatibility. However, artificially produced materials are recognized for their good mechanical properties [5]. Ultimately, easy manufacturing and processing, affordability, and commercial availability are crucial factors in choosing the appropriate bioink [2].

Bioprinting using collagen hydrogel is a promising technology for tissue engineering and regenerative medicine due to the natural abundance of collagen in the extracellular matrix of many tissues and its biocompatibility [6]. Collagen, being the primary structural protein of extracellular matrix (ECM) and having a strong affinity for adherent cells, is commonly utilized in biomedical applications [7]. Collagen hydrogels find their usage in a wide range of fields, ranging from testing materials for 3D bioprinting to creating general tissue models for in vitro cell studies [8] and drug testing [9]. They are also used to develop specialized tissue models for various applications such as epithelium [9], neural [10], skin [11], or osteogenic [12], especially when cell-laden hydrogels are used. High-concentrated collagen hydrogels have shown great potential in tissue engineering due to their favorable mechanical and structural properties, making them suitable for creating complex tissue constructs that closely resemble native tissue. Furthermore, collagen can be easily modified to mimic specific tissue types, allowing the creation of tissue-specific constructs [13]. On the other hand, collagen is a natural material that can be extracted from various animal and waste sources. Therefore, its integrity, structural stability, resistance to different treatments, and gelling ability can be influenced by several factors, such as the source of collagen and the isolation method. Thus, the properties of collagen can vary between batches, which can lead to inconsistencies in the quality and properties of the printed constructs [14]. In addition, collagen has relatively poor mechanical strength and stability, which can limit its use in creating larger, more complex structures [15]. This can be improved by using collagen with a high protein concentration (>10 mg/mL), but due to the solidification of the hydrogel, when the physical conditions (temperature, pH) change, the meltability of the material during printing is relatively high [16]. In general, it is also considered that high protein concentration in collagen hydrogels can lead to poor cell viability and limited functionality of printed constructs [17]. Finally, collagen is relatively expensive compared to other biomaterials, which can make it less accessible to some researchers [18].

The stiffness of collagen hydrogels varies depending on various factors, such as collagen concentration, crosslinking agent, and printing parameters [19]. Generally, high-concentrated collagen hydrogels have been shown to have favorable mechanical properties for tissue engineering applications, but their stiffness can still be lower than that of native tissue [20]. However, there are techniques to improve the stiffness of bioprinted collagen hydrogels, such as crosslinking with chemical agents or incorporating other materials into the hydrogel. In the past decade, many synthetic photosensitive biomaterials have been developed that allow for the spatiotemporal control of cross-link formation by light [21]. While these materials offer precise control over material and chemical properties, they often require additional modifications to maintain bioactivity and biodegradability for use as scaffolds for tissue regeneration.

Similarly, natural polymers such as collagen are modified to increase their mechanical resistance and printing accuracy [22]. Collagen modified with methacrylate groups achieves better mechanical stability and greater control over the crosslinking process, resulting in more precise and consistent printing [16]. This modification also enables the incorporation of other functional groups into the collagen, such as growth factors or drugs, for targeted delivery in tissue engineering and regenerative medicine applications [23]. Other methods involved modification of the collagen with acrylate or methacrylate groups prior to crosslinking with an activated photoinitiator, leading to undesirable gelation during the reaction or partial denaturation of the collagen [24]. Methacrylate collagen has shown promising results in developing complex tissue constructs with improved mechanical properties and cell viability [25]. However, methods of photo cross-linking fibrillar hydrogels of type I collagen have significant limitations. For example, some methods required UV irradiation in the presence of flavin mononucleotide, which resulted in minimal changes in mechanical properties and required crosslinking prior to self-assembly [26]. 

Young’s modulus of collagen hydrogels then ranges from a few hundred Pa to a few kPa, depending on the crosslinking method used, but is still much lower than that of native tissue [27]. On the other hand, excessively cross-linked collagen may have a structure different from natural collagen, which can result in changes in the rate of gel degradation and mechanical properties. Moreover, it may also increase the probability of cytotoxicity [28], when some crosslinking agents can be toxic to cells and may reduce cell viability [29]. It has been shown that increasing the pH to 7.3 and the temperature to 37 °C of the collagenous solution can induce the crosslinking of the entire structure, which is then strong enough to form a stable 3D construct and does not reduce the survival of cells incorporated into the hydrogel structure [30].

However, ensuring the viability of cell culture within bioprinted hydrogels remains a challenge. The flow of the surrounding medium is essential to provide a continuous supply of nutrients and gases and the removal of metabolites, leading to improved cell proliferation, differentiation, and production of the extracellular matrix [31]. This method has been successfully applied to various tissue types, including bone, cartilage, and muscle [31]. Incorporating active perfusion during cultivation can significantly improve the functionality and longevity of tissue constructs while exposing cells to a more physiologically relevant environment [29]. The combination of bioprinting and active media perfusion has an excellent potential to create complex tissue constructs that closely resemble native tissue. Overall, this approach can significantly advance the field of regenerative medicine and pave the way for the development of functional tissue replacements. Therefore, based on our previous experience with decellularized and recolonized pericardia in the application of cardiovascular patches [32,33], we investigated the effect of active media perfusion of a highly concentrated bioprinted collagen hydrogel on cell viability with a comparison of a proliferation growth culture medium and a smooth muscle cell differentiation culture medium.

## 2. Results and Discussion

### 2.1. Titration of Collagen and Determination of Optimal NaOH Addition

**Figure 1:**
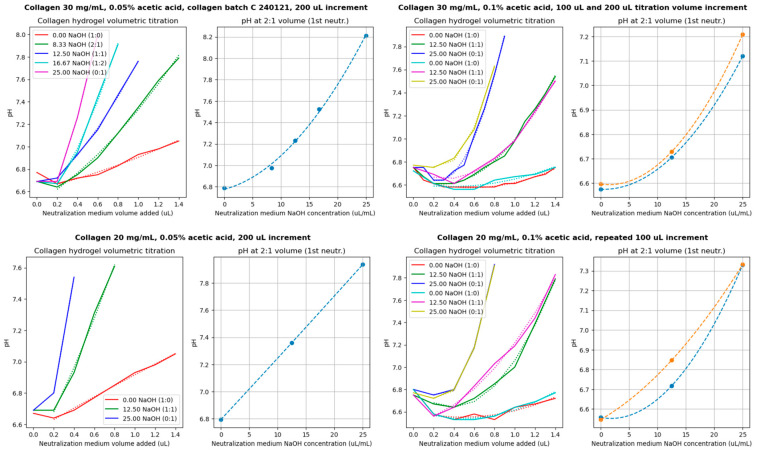
Volumetric titration curves for determining the addition of NaOH to ensure the optimal pH of the bioink after first neutralization (7.0–7.2). Dotted lines represent 2nd order polynomial fit of measured data or prediction model.

Figure 1 shows the volumetric titrations of collagen hydrogel prepared at collagen concentrations of 20 mg/mL and 30 mg/mL dissolved in 0.05% and 0.1% acetic acid. The volumetric increment was 100 μL or 200 μL of the neutralizing medium.

In the graph for a collagen concentration of 30 mg/mL and 0.1% acetic acid, the test increments were 200 μL. Then, the same ratios were repeated with increments of 100 μL to ensure that the mixing system presented in our previous article [34] dosed the correct medium amount. The results showed that they differed minimally. However, the volumetric titration for collagen 20 mg/mL and 0.1% acetic acid, which was tested the same way but first with increments of 100 μL and then repeated in the same ratios with increments of 100 μL, resulted in a slight change in a final titration curve due to the lack of material. 

For future collagen volumetric titrations of new batches, titration points can be obtained by adding only 650 μL for each ratio. 

### 2.2. Mechanics of the Collagen Hydrogel

**Figure 2:**
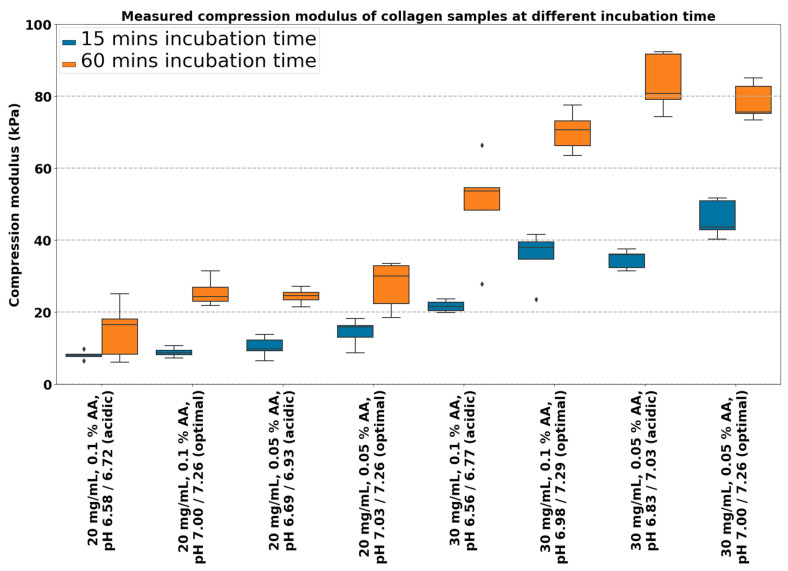
Compression modulus of collagen samples for 20 and 30 mg/mL concentrations with pH uncompensated (acidic) and optimal at 15 and 60 min incubation time. Boxplots with inner lines represent the median values; box borders 1st and 3rd quartiles; whiskers are the minimum and maximum; and the dots are outlier values.

Figure 2 shows the compression moduli of collagen hydrogels prepared from collagens at 20 and 30 mg/mL concentrations dissolved in 0.05 and 0.1% acetic acid. The resulting pH was set in two manners: (1) acidic with no compensation and (2) compensated to optimal pH according to the titration curves and predicted models. Each sample was measured approximately 15 and 60 min after printing (with N = 5) for each sample. There is a significant difference in compression modulus between 20 and 30 mg/mL with different types of preparation. Therefore, collagen concentration plays the main role in overall stiffness (unpaired *t*-test; for more details, see Supplementary Material S1).

According to linear regression parameter estimates, all three parameters significantly affect the resulting stiffness of collagen bioink see Table 1 and Table 2. The increase in strength over time is evident. After 60 min, the measured modulus did not increase further; therefore, the gels reached their final stiffness (model estimate ‘Incubation time’). A comparison of gels prepared at different concentrations of acetic acid showed an increased compression modulus of gels prepared in 0.05% AA for both collagen concentrations (model estimate ‘AA concentration’—negative coefficient).

The collagen titration method was essential at the beginning of bioink preparation. Without the compensation of pH with the addition of NaOH, the gel’s stiffness is significantly reduced. However, the addition of NaOH in the optimal ratio also significantly increased gel stiffness (model estimate ‘pH optimization’). 

The compressive modulus of collagen hydrogels is known to increase with increasing collagen concentration because collagen molecules form a densely packed network at higher concentrations, resulting in higher mechanical strength and stiffness of the hydrogel [35]. However, there is a limit to how much the compressive modulus can increase with an increasing concentration, as excessively high concentrations can form a more brittle and less elastic hydrogel [35]. Determining a specific value for the compression modulus of collagen hydrogels is challenging. Each research group uses its own methodology for preparing and measuring hydrogels, so it is not easy to generalize and compare the results. 

Acetic acid is used to solubilize collagen and adjust the pH for gelation. The acetic acid concentration can affect the hydrogel’s structure and mechanical properties [36]. An increasing acetic acid concentration can form a denser network of collagen fibrils, resulting in a higher Young’s modulus. However, other sources state that a higher concentration of acetic acid can lead to a lower pH and a less crosslinked collagen structure, resulting in a lower Young’s modulus [37]. Careful control of the concentration of acetic acid is essential for achieving the desired mechanical properties for tissue engineering applications.

Acetic acid and sodium acetate (formed during the neutralization of acetic acid by sodium hydroxide) are typical examples of buffers. A buffer refers to a solution that can withstand changes in pH when an acidic or basic component is added. It has the ability to neutralize small amounts of added acid or base, thereby keeping the pH of the solution relatively constant and stable. If the concentration of acetic acid is lower, then the buffering capacity of the entire system is also lower. This makes it easier to achieve a neutral pH, which, in turn, leads to the formation of a gel.

### 2.3. Cell Viability and Morphology

**Figure 3:**
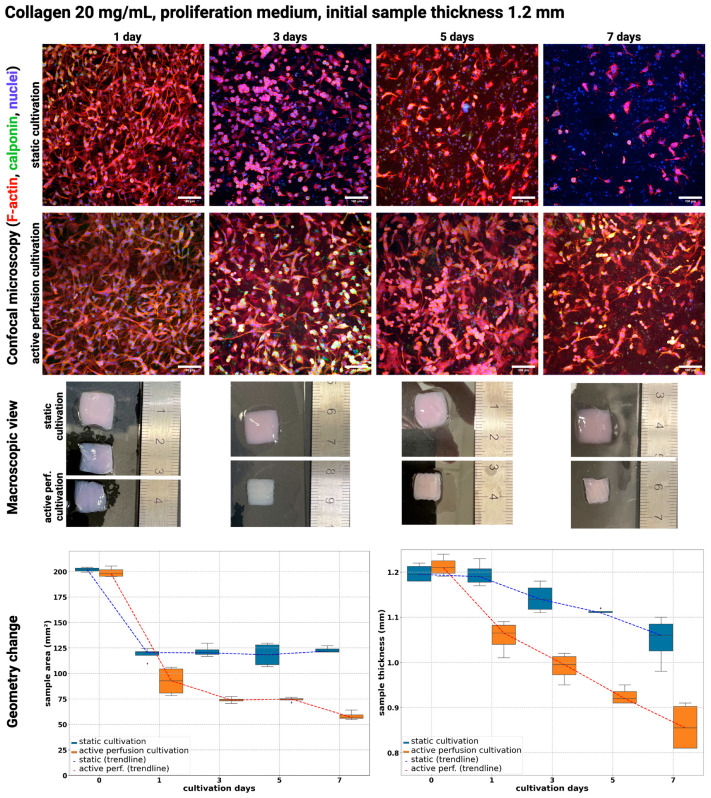
Confocal microscopy images (**top**) of cultivated samples in 20 mg/mL collagen and their macroscopic view (**middle**) and geometrical change of samples area and thickness (**bottom**). Cultivation for 1, 3, 5, and 7 days in a proliferation culture medium under static and dynamic conditions. F-actin was stained using Alexa Fluor™ 594 Phalloidin (red), Calponin H1 with Alexa Fluor™ 488 (green), and cell nuclei were counterstained using DAPI (blue), scale 100 µm. Nikon CSU-W1 confocal microscope with CFI Plan Apo VC 20× dry objective. Orthographic MIP-rendered projection. Boxplots with inner lines represent the median values; box borders 1st and 3rd quartiles; whiskers are the minimum and maximum; and the dots are outlier values.

**Figure 4:**
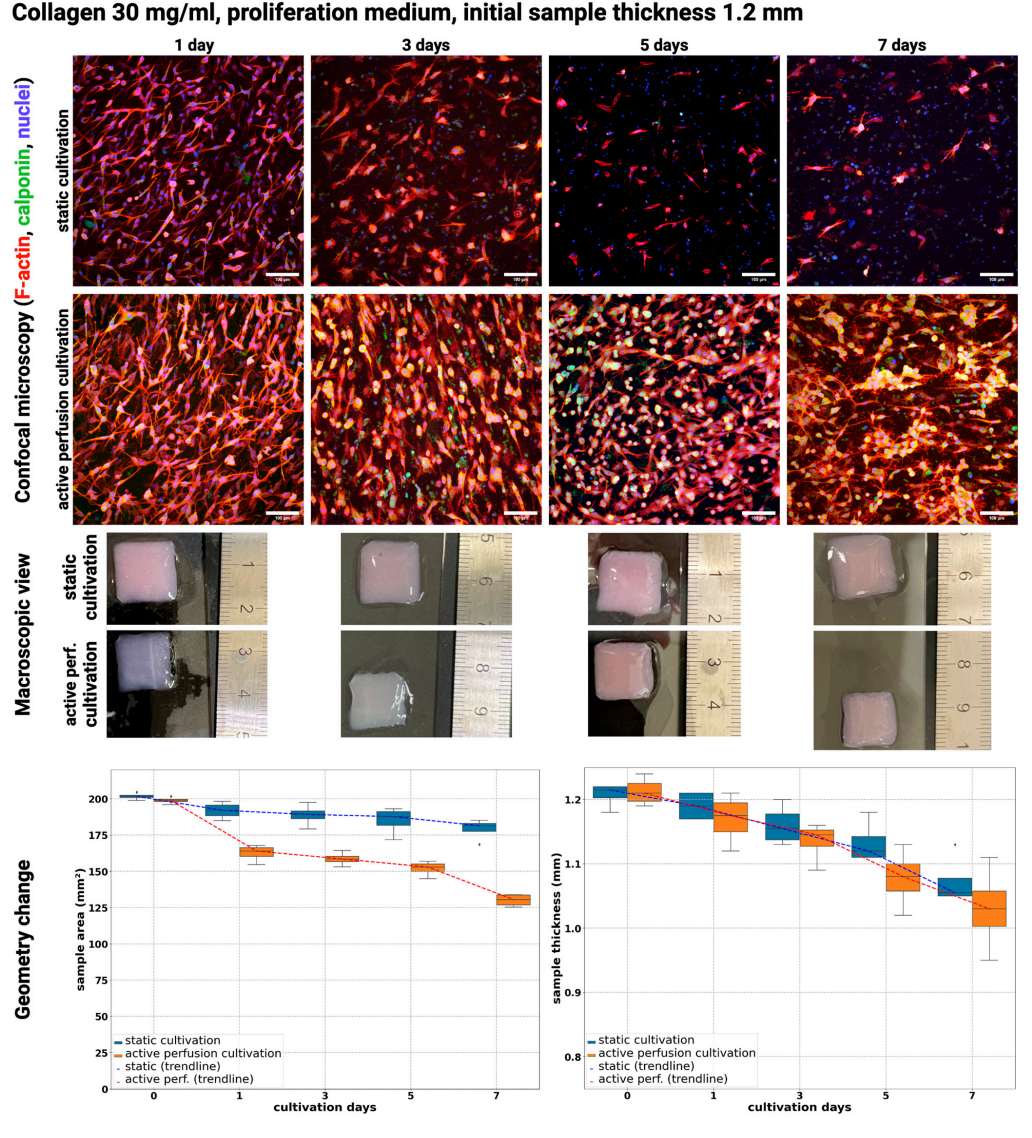
Confocal microscopy images (**top**) of cultivated samples in 30 mg/mL collagen and their macroscopic view (**middle**) and geometrical change of samples area and thickness (**bottom**). Cultivation for 1, 3, 5, and 7 days in a proliferation culture medium under static and dynamic conditions. F-actin was stained using Alexa Fluor™ 594 Phalloidin (red), Calponin H1 with Alexa Fluor™ 488 (green), and cell nuclei were counterstained using DAPI (blue), scale 100 µm. Nikon CSU-W1 confocal microscope with CFI Plan Apo VC 20× dry objective. Orthographic MIP-rendered projection. Boxplots with inner lines represent the median values; box borders 1st and 3rd quartiles; whiskers are the minimum and maximum; and the dots are outlier values.

Figure 3 and Figure 4 show a comparison of static culture and active perfusion ‘dynamic’ culture samples when cultured in a growth culture medium in the hydrogel with an initial collagen concentration of 20 or 30 mg/mL. In both types of gels, a decrease in cell number can be observed in static culture from the beginning to day 7 of culture. Cell loss was insignificant on the third day of culture, but cells were round, indicating poor cell viability. On the fifth day of culture, there was already a noticeable decrease in cell number, and by the seventh day, almost no viable cells were found in the culture. The culture of the samples with active perfusion stimulated the cells to grow and divide, and the cells elongated and intermingled. Their number increased until the fifth day of culture, but by the seventh day, their number was reduced because their differentiation potential was suppressed by growing in a proliferative culture medium.

In bioprinted collagen hydrogels, the typical cell morphology can vary depending on the specific cell type used for culture. Generally, cells in collagen hydrogels exhibit a rounded morphology as they adapt to their new environment and begin to proliferate. As cells grow and remodel the surrounding collagen matrix, they can take on a more elongated or spindle-like shape. Cells embedded deep in the hydrogel, especially in static cultures, have a poor nutrient supply, so their shape is often round. However, the cells on the surface are already elongated like fibroblasts [38]. In general, bioprinted collagen hydrogels that support high cell viability and functionality tend to exhibit well-organized, elongated cell structures that resemble native tissue [39], which was achieved in our samples with active perfusion.

Moreover, cells remodeled the printed substrate according to its dimensional changes, as illustrated in macroscopic views and charts in Figure 3 and Figure 4. According to linear regression estimates, all observed parameters have a significant role in substrate remodeling; see Table 3 and Table 4. The type of cultivation promoted over a prolonged cultivation period has the most significant impact on geometry change. 

In 20 mg/mL bioink, there was a change in area from approx. 200 mm^2^ to approx. 60 mm^2^ when active perfusion for 7 days was used, whereas in static conditions, it was lowered to approx. 125 mm^2^. Especially for static cultivation, there was a major change after 1 day of cultivation; however, this change stagnated. In active perfusion, the change was observed on all days of cultivation. Thickness was reduced from approx. 1.2 mm to 0.85 in active perfusion to 1.06 in static. 

In 30 mg/mL bioink, there was a change in area from approx. 200 mm^2^ to approx. 125 mm^2^ when active perfusion for 7 days was used, whereas in static conditions, it was lowered to approx. 175 mm^2^. This change developed on all days of cultivation. Thickness was reduced from approx. 1.2 mm to 1.05 mm in active perfusion to 1.07 mm in static. This thickness change is not so high compared to 20 mg/mL collagen but is still statistically significant according to the linear regression model.

An increased rate of remodeling in active perfusion correlates with higher viability and metabolic activity.

**Figure 5:**
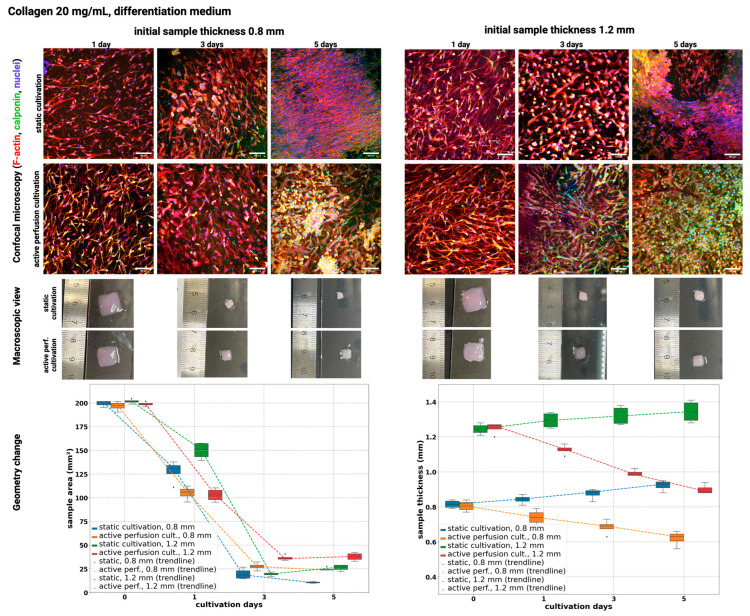
Confocal microscopy images (**top**) of cultivated samples in 20 mg/mL collagen and their macroscopic view (**middle**) and geometrical change of samples area and thickness (**bottom**). Culturing for 1, 3, and 5 days under static and dynamic conditions in SMC differentiation culture medium. F-actin was stained using Alexa Fluor™ 594 Phalloidin (red), Calponin H1 with Alexa Fluor™ 488 (green), and cell nuclei were counterstained using DAPI (blue), scale 100 µm. Nikon CSU-W1 confocal microscope with CFI Plan Apo VC 20× dry objective. Orthographic MIP-rendered projection. Boxplots with inner lines represent the median values; box borders 1st and 3rd quartiles; whiskers are the minimum and maximum; and the dots are outlier values.

Figure 5 shows the results of static and active perfusion ‘dynamic’ culturing in hydrogels at 20 mg/mL concentrations for two hydrogel thicknesses when cultured in a culture medium for differentiation into smooth muscle cells. The results were relatively comparable for static culture and culture with active perfusion regarding proliferation and cell number. In both cultures, there is an increase in cell number at all intervals; the cells are elongated and, by the fifth day, have already formed a compact structure. However, there is shrinkage and thickening of the collagen hydrogel at the same time, and on day 7, due to collagen remodeling, the shrinkage was so significant that the results could not be analyzed without distortion of the data. 

However, there is a significant difference between the two cultures in producing calponin, which regulates the growth and differentiation of smooth muscle cells. On the first day of culture, the differences are not significant. However, on the third day of culture, the calponin production is several times higher in the culture with active perfusion, as well as on the fifth day of culture. This indicates a much higher potential for cells to differentiate into smooth muscle when cells are supported by active perfusion, which agrees with the existing literature [29,31]. 

**Figure 6:**
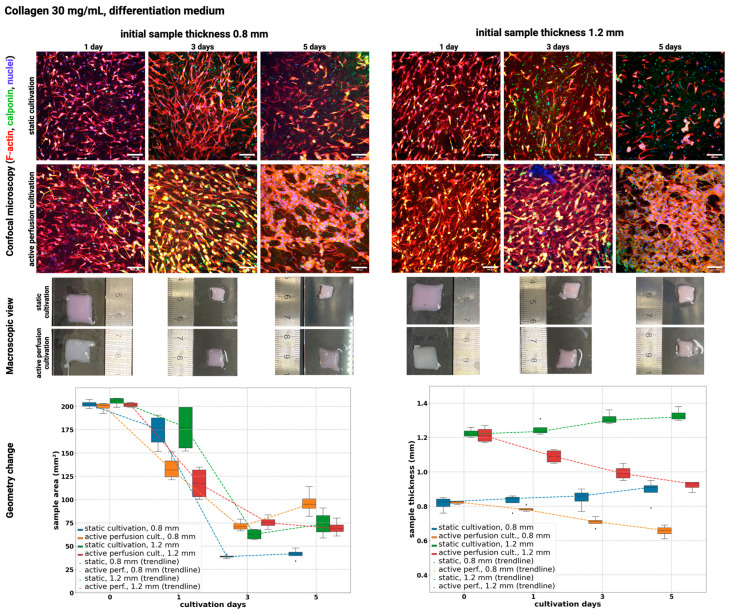
Confocal microscopy images (**top**) of cultivated samples in 30 mg/mL collagen and their macroscopic view (**middle**) and geometrical change of samples area and thickness (**bottom**). Culturing for 1, 3, and 5 days under static and dynamic conditions in SMC differentiation culture medium. F-actin was stained using Alexa Fluor™ 594 Phalloidin (red), Calponin H1 with Alexa Fluor™ 488 (green), and cell nuclei were counterstained using DAPI (blue), scale 100 µm. Nikon CSU-W1 confocal microscope with CFI Plan Apo VC 20× dry objective. Orthographic MIP-rendered projection. Boxplots with inner lines represent the median values; box borders 1st and 3rd quartiles; whiskers are the minimum and maximum; and the dots are outlier values.

Figure 6 shows the results of static culture and culture with ‘dynamic’ active perfusion in a 30 mg/mL hydrogel for two hydrogel thicknesses when cultured in a culture medium for differentiation into smooth muscle cells.

For these samples, the resulting collagen concentration is 15 mg/mL, which is already a relatively high concentration for cells in static culture, resulting in gradual cell death, especially in the thicker sample in which there is insufficient diffusion of gases and metabolites. Despite this, the cells form an elongated shape, indicating partial differentiation of the cells into smooth muscle cells, as shown by the SMC marker calponin. On the other hand, under active perfusion, the cells prosper, their number increases, and in 5 days, they form a fully grown structure with high differentiation toward SMC. As in the previous case, the collagen hydrogel was so remodeled and shrunken that it was impossible to evaluate the results on day 7 without biasing the data. 

During cultivation, the shrinkage and remodeling of hydrogel samples from both types of cultures occurred independently of the collagen concentration. Hydrogel shrinkage is a natural process that occurs in cell cultures with physical stresses (pressure, electric field, etc.) [40]. Other mechanisms of its formation include dehydration or cell contraction [40]. The shrinkage process is essentially the repeated spreading of cells, their pull on the hydrogel in the radial direction, and the reduction of hydrogel elongation over time [41]. The shrinkage can lead to changes in the mechanical properties of the hydrogel, affecting cell behavior and tissue development. Gel shrinkage induces changes in mass transfer efficiency, cell distribution, and density of adhesive ligands of the surrounding matrix [42].

Shrinkage can be beneficial, as it can lead to increased mechanical stability and improved cell alignment within the hydrogel; on the other hand, excessive shrinkage can negatively impact the viability and functionality of cells within the hydrogel, as it can lead to increased stress and strain on cells [40]. In our samples, the reduction of the pattern area from approx. 200 mm^2^ (initial size 14 × 14 mm) dropping to 25 mm^2^ was achieved (Figure 3, Figure 4, Figure 5 and Figure 6). Morphology and viability assays showed that even under this significant change, cells are still viable (in samples with active media perfusion) and form the structure of future tissue. The reduction in the area of the sample is further supported by the fact that the cells metabolize the surrounding collagen into their own extracellular matrix, thus remodeling the entire sample.

To demonstrate this fact, we again evaluated cell area and sample thickness for 20 and 30 mg/mL collagen, both cultivations and initial print heights in the differentiation medium. Also, linear regression model estimates were made (see Table 5 and Table 6). The differentiation medium promoted remodeling and collagen shrinkage in both cultivation types. Not even a significant difference was achieved by type of cultivation, which was also confirmed by the regression model.

However, there was one interesting finding: in the proliferation medium, the cells were able to remodel structure in terms of area and thickness in the same manner as in the differentiation medium; the thickness change was inverse (negative rank for cultivation in the regression model). In active perfusion, there was a similar scenario as in proliferation media. The initial thickness was reduced from approx. 1.2 or 0.8 mm to approx. 0.9 or 0.6 mm. On the other hand, in static conditions, the thickness increased over cultivation time from an initial approx. 1.2 or 0.8 mm to approx. 1.3 or 0.9 mm. We believe this is caused by shrinkage caused by calponin active and viable cells on the top side of the sample, whereas mainly dead or apoptotic cells are on the bottom side. The active perfusion allows overall diffusion; thus, both sides have viable cells, and substrates are remodeled homogenously. The dynamic samples were homogenously reduced in terms of thickness, and the static samples created more blob-like structures, as seen in cell morphology. 

**Figure 7:**
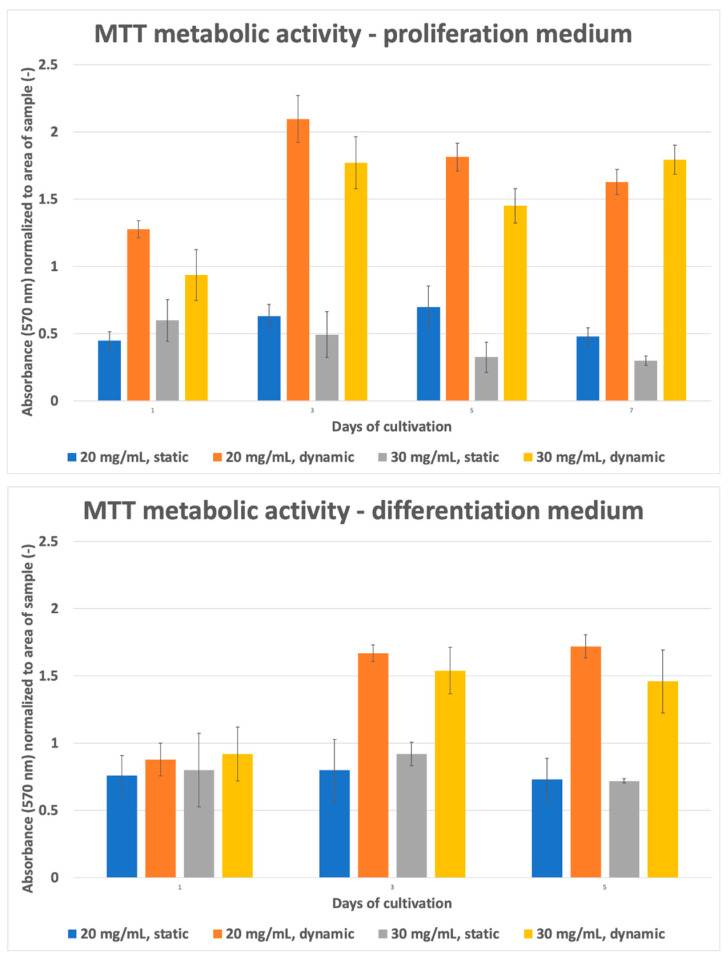
MTT metabolic activities for different collagen concentrations and types of cultivation.

In addition, the MTT metabolic activity was determined. These MTTs are collated with confocal images and LD staining. The metabolic activity in dynamic culture is significantly higher than in static culture, as illustrated in Figure 7. 

**Figure 8:**
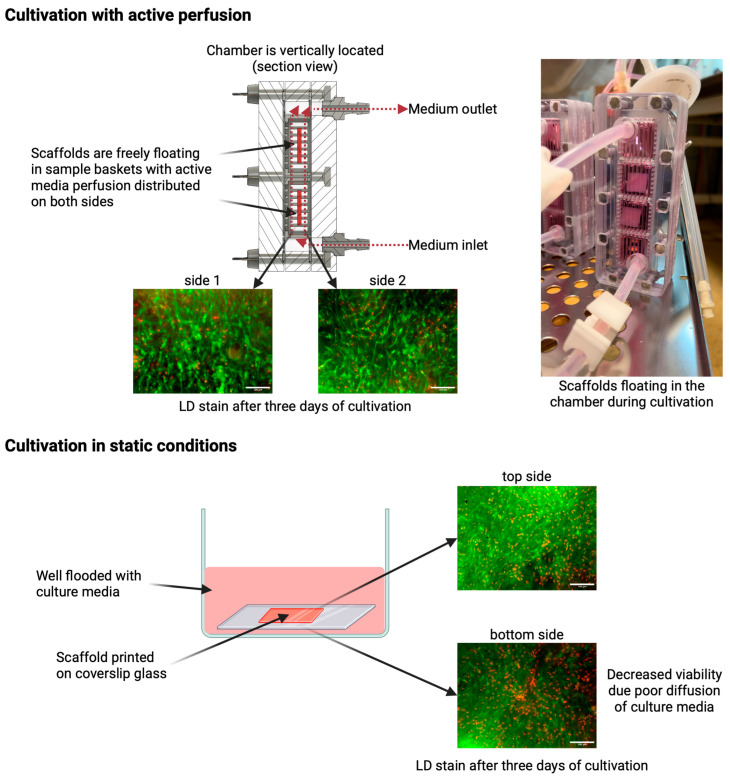
(**Above**) Culturing with active perfusion results in media flow around the samples, which float freely in the media stream. Nutrient and gas diffusion is enhanced, resulting in high viability in all gel layers. (**Bottom**) Static culture samples adhere to the slide. Due to the reduced diffusion concentrated on only one side of the gel, cell viability is reduced, especially on the bottom side.

**Figure 9:**
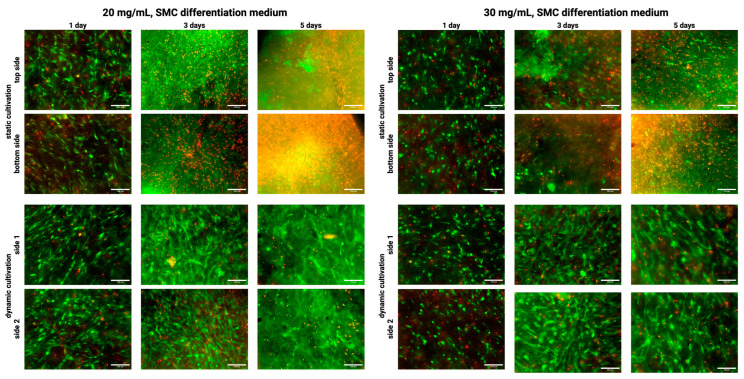
Cell viability for 20 and 30 mg/mL collagens in static and active media perfusion ‘dynamic’ cultivation in differentiation culture medium after 1, 3, and 5 days of cultivation. The images show both sides of each sample to demonstrate the unequal distribution of dead cells in the static sample, as the part of the gel adhered to the slide is not sufficiently nourished. Live cells are fluorescing green due to FDA uptake, and dead cells are fluorescing red due to PI uptake.

Several factors can affect cell viability in collagen hydrogel, including collagen concentration in the hydrogel, stiffness, porosity, or hydrogel geometry [43]. The high protein concentration in the hydrogel can lead to poor cell viability and limited functionality of the printed constructs. Nutrient supply and/or removal of waste products is critical to maintaining cell viability in a dense collagen gel, which was proven by an experiment where the viability of the static sample was only 20% compared to the perfused sample with 80% live cells [44]. Incorporating active perfusion during cultivation can significantly improve the functionality and longevity of tissue constructs while cells are exposed to a more physiologically relevant environment [45]. The same results were obtained in our study. In samples with active perfusion (Figure 8 above), the culture medium flows around the entire gel surface, increasing the diffusion efficiency and exchange of gases and metabolites. The sample is well populated with dead cells evenly distributed on both halves. The gels adhere to the culture glass on one side for static samples, and the sample is flooded with culture medium in the well (Figure 8 below). Diffusion is, thus, limited to surfaces without a substrate barrier, insufficient to nourish cells located inside and towards the bottom edge. Therefore, cell viability is reduced at these sites, as shown in Figure 9. Utilizing hydrogels in bioprinting can shield cells from shear stress and consequent membrane damage, thereby improving their viability in the printed structure. Mechanical stresses experienced during extrusion can damage cell membranes and result in decreased cell viability [46].

**Figure 10:**
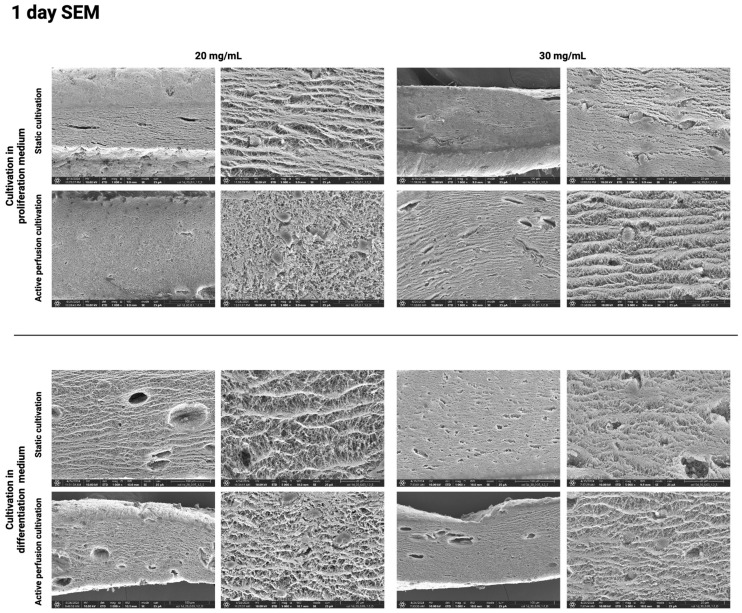
Hydrogel architecture visualized by SEM microscopy after 1 day of cultivation for both collagen concentrations (20 and 30 mg/mL), both culture media, and static and active perfusion cultivation.

**Figure 11:**
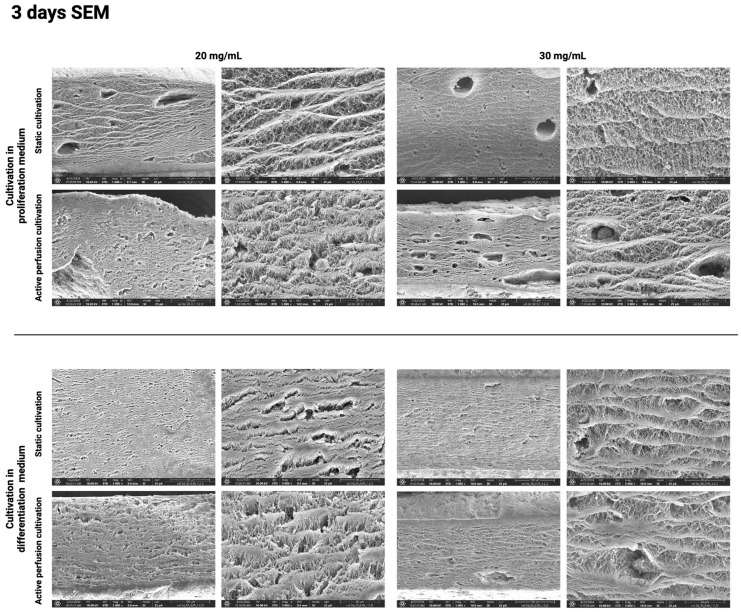
Hydrogel architecture visualized by SEM microscopy after 3 days of cultivation for both collagen concentrations (20 and 30 mg/mL), both culture media, and static and active perfusion cultivation.

**Figure 12:**
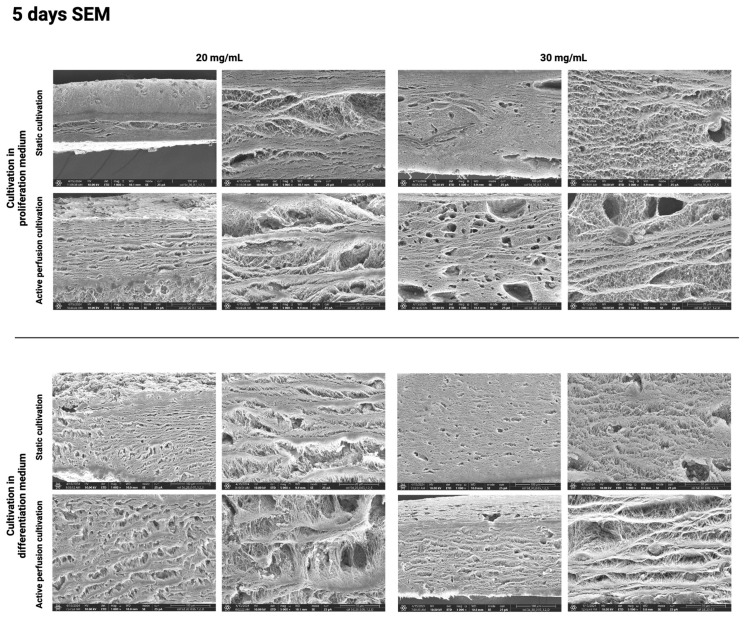
Hydrogel architecture visualized by SEM microscopy after 5 days of cultivation for both collagen concentrations (20 and 30 mg/mL), both culture media, and static and active perfusion cultivation.

**Figure 13:**
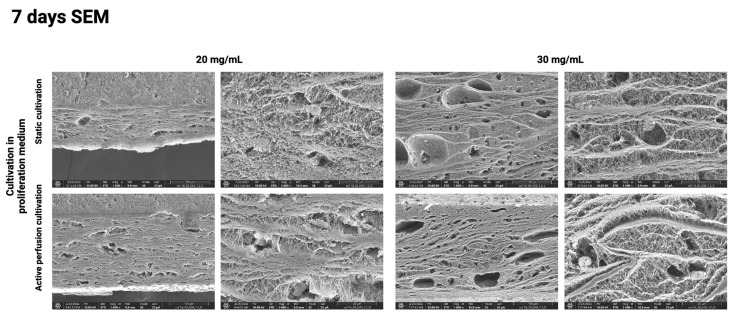
Hydrogel architecture visualized by SEM microscopy after 7 days of cultivation for both collagen concentrations (20 and 30 mg/mL) and static and active perfusion cultivation.

The above results were further confirmed using the SEM method, which visualizes the hydrogel surface in detail. Figure 10, Figure 11, Figure 12 and Figure 13 show the difference in the inner architecture of the gels after 1, 3, 5, and 7 days of cultivation. On the first day of cultivation, the hydrogels are compact, without significant cavities, except for naturally occurring air bubbles. The compactness of the gel is higher in the case of a higher collagen concentration (30 mg/mL). Cell growth through the hydrogel can be observed on the third and fifth day of culture. Cells contract the gel and remodel the collagenous matrix into its own extracellular matrix. It can be observed that the occurrence of cavities and pores is more pronounced in the active perfusion system. Finally, on the seventh day, the structural changes are the most significant, with the appearance of empty spaces around individual cells, which remodel the collagen matrix.

**Figure 14:**
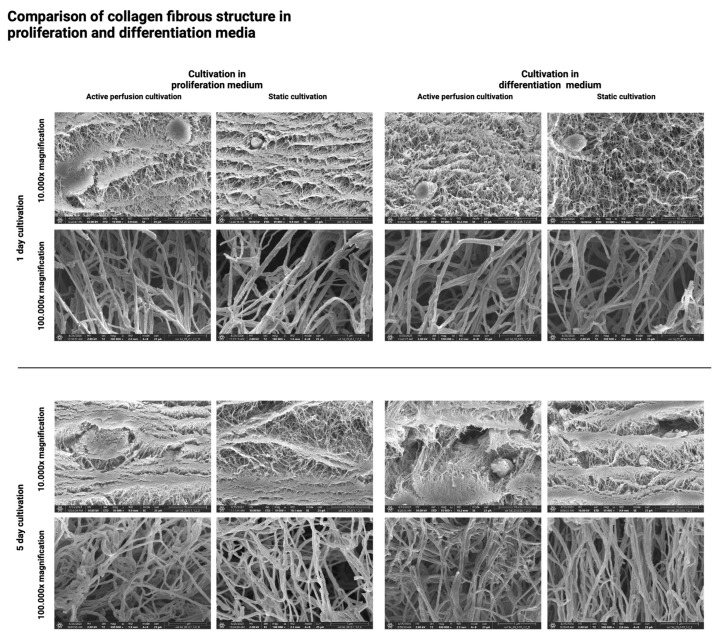
Comparison of collagen fibrous structure visualized by SEM microscopy for proliferation and differentiation medium after 1 and 5 days of static and active perfusion cultivation.

The changes that occur in the collagen hydrogel’s internal structure are clearly visible at the macroscopic level, i.e., at tens to hundreds of micrometers. The contrary would be at the microscopic level, i.e., at the level of nanometer units, when individual collagen fibers or filaments are already visible (Figure 14) without their geometrical change over the cultivation period.

Static cultivation with both proliferation and differentiation media has reduced viability. In proliferation media, a large number of apoptotic cells or cell debris were observed in confocal images. Also, according to the SEM images and the geometrical change, the remodeling was minimal. In differentiation media, surface cells were partially differentiated into SMC-like cells. Apoptotic cells in lower layers caused asymmetric tension and remodeling, generating a blob-like formation of the sample. This was also confirmed by the increased thickness of samples after cultivation.

Active perfusion with proliferation media maintains viable cells in overall volume with homogenous remodeling of the substrate as contrasted to SEM images. 

Active perfusion with a differentiation medium promotes partial cell differentiation into SMCs within the whole volume and cell viability with strong substrate remodeling and shrinkage. This is demonstrated with significant geometrical change.

**Figure 15:**
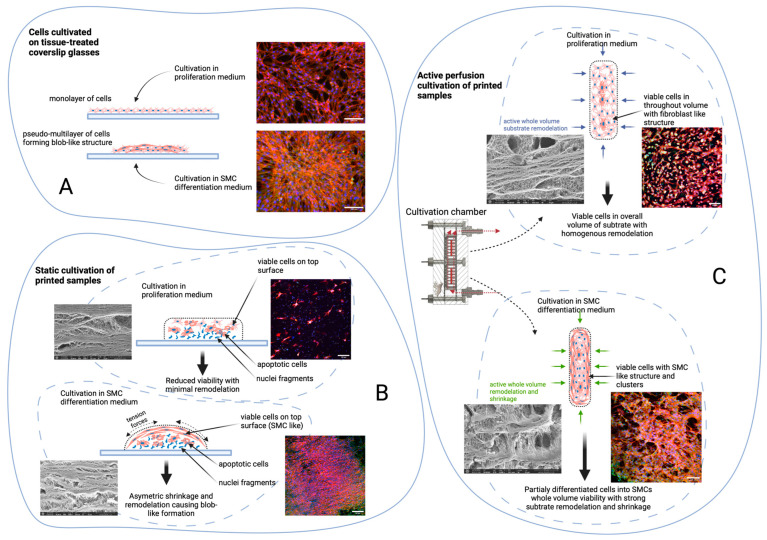
Summary of different types of cultivation and their effect on cell viability and proliferation.

When comparing the samples of all culture types and culture media, it can be concluded that the best optimal morphological proliferation is achieved by the bioink sample with active perfusion with the SMC differentiation culture medium. In this sample, cells have high viability throughout the culture period, an optimal elongated shape with protrusions, high calponin production, and a high degree of remodeling of the collagen hydrogel in the ECM. This confirms that the choice of culture with active proliferation and the promotion of cell differentiation by culture medium chemicals have a significant impact on the success of cell cultures in highly concentrated collagen hydrogels. All these milestones are illustrated in Figure 15. 

## 3. Conclusions

In conclusion, bioprinting of high-concentrated collagen hydrogels holds excellent potential for tissue engineering and regenerative medicine applications. In this study, we have shown that incorporating active media perfusion while cultivating highly concentrated collagen hydrogels significantly improves cell viability and metabolic activity. We used our collagen isolated from porcine skins, and to ensure consistent results, we developed a method to provide the same pH and hydrogel properties each time the bioink was prepared, regardless of the batch. A porcine skin collagen derived (of initial concentrations of 20 and 30 mg/mL and final collagen concentration in bioink of 10 and 15 mg/mL) was used for the experiments. Our results showed that active media perfusion significantly enhances cell culture viability in bioprinted collagen with high protein concentrations. Using a differentiation medium promotes the growth and differentiation of cells in the hydrogel. In contrast, suppression of cell differentiation by using a growth medium leads to cell apoptosis. The method of active perfusion can provide a continuous supply of nutrients and gases, enhancing cell proliferation, differentiation, and extracellular matrix production. Its combination with bioprinting can pave the way for the development of functional tissue replacements that closely resemble native tissue. This approach could be useful in developing functional tissue constructs for various applications in regenerative medicine.

## 4. Materials and Methods

### 4.1. Collagen Titration

**Figure 16:**
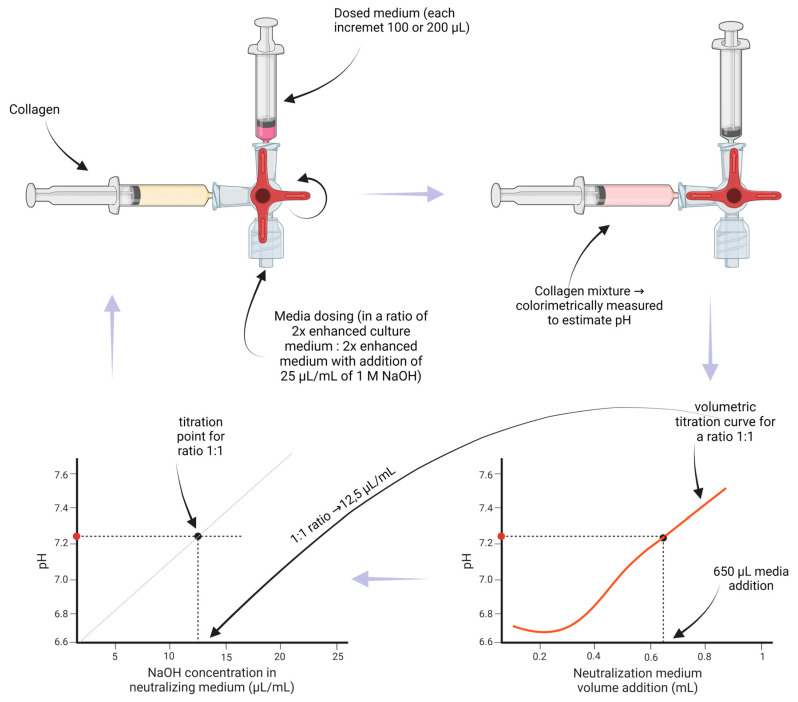
A schematic process for collagen titration using different ratios to receive a volumetric titration curve and a titration point.

**Figure 17:**
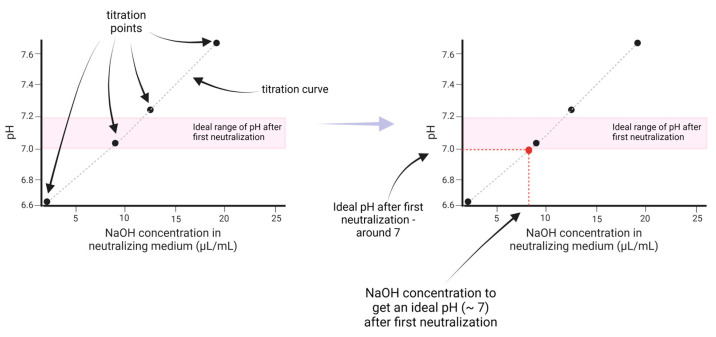
The estimation procedure to obtain the ideal ratio for the first neutralization to have a pH between 7 and 7.2. The obtained titration points (**left**) were interpolated with a titration curve. The ideal NaOH concentration in the medium equals the x-value for the intersection of the titration curve and the pH value (**right**).

Since each batch of collagen is different, we proposed a method (see Figure 16 and Figure 17 and Appendix B) to determine the ideal ratio (of a 2× enhanced culture medium and a 2× enhanced culture medium with the addition of 25 μL/mL of 1M NaOH) used to obtain a pH between 7 and 7.2 after the first neutralization of collagen. For each collagen, with concentrations of 20 mg/mL and 30 mg/mL dissolved in 0.05% and 0.1% AA, volumetric titration curves were prepared. To 1300 μL of collagen, a neutralizing medium was added in 100 or 200 μL increments (till 1300 μL) for different ratios of culture medium (2× enhanced medium: 2× enhanced medium with added NaOH). The ratios used were mainly 1:0 (i.e., using only medium without NaOH addition), 0:1 (i.e., using only medium with NaOH addition), 1:1, and other ratios to obtain a more accurate titration curve. The components were thoroughly mixed for each increment, and the colorimetric value of the mixture was measured to estimate the pH. After enough ratios were performed, a titration curve for a first neutralization was obtained from the neutralization points, obtained from the pH value for an addition of 650 μL of the neutralizing medium (y-axis) and from the titration ratio (x-axis). 

### 4.2. Bioink Preparation 

For the experiments, we used collagen isolated from porcine skin. All the isolation, hydrogel preparation, and characterization processes are published in our previous article [34]. The collagen concentrations in the hydrogel are 20 mg/mL and 30 mg/mL, dissolved in 0.05 and 0.1 vol.% acetic acid (AA).

To prepare the printable collagen bioink, we followed a two-step neutralization method presented in our previous article [34]. First, the collagen hydrogel is neutralized to a pH of approximately 7 using the correct ratio (of 2× enhanced culture medium and 2× enhanced culture medium with the addition of 25 μL/mL of 1M NaOH) obtained from the titration curves. The cell suspension is then added. During these procedures, it is crucial to accurately dose the collagen hydrogel and add a 2× enhanced culture medium with the optimized pH of the preconditioning. These components are dosed in small volumes of hundreds of microliters. Even a slight volume change can significantly affect the pH, printing, and gelling parameters of the prepared collagen bioink. A custom semi-automated mixing system has been developed and described in our previous article [40] to ensure precision and repeatability. A new continuous mixing with a set ratio was implemented. This allows the mixing ratio to be set in the range of 0–10 for the basic and NaOH-enriched medium, resulting in a different pH. Ratio-based control is used due to the stepper motor that is actuating reservoir syringes. The dosed volume is divided into ratio segments. Both stepper motors are running simultaneously to ensure mixing during dosing.

### 4.3. Compressive Modulus Measurement

**Figure 18:**
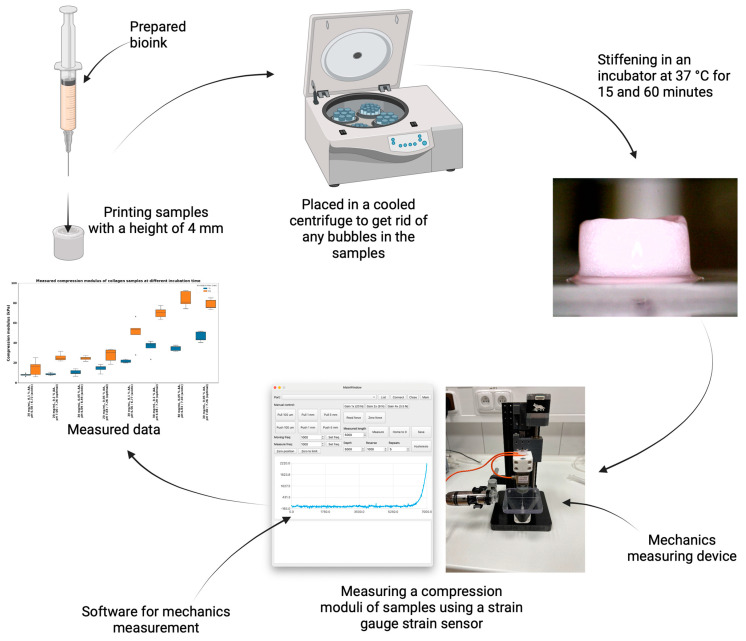
The workflow of measuring the compressive modulus of samples.

To measure the compression moduli of collagen hydrogels, for each collagen (with concentrations of 20 mg/mL and 30 mg/mL dissolved in 0.05% and 0.1% AA), two bioinks were prepared, one for an ideal titration ratio and the other with a ratio for 0 NaOH. Each bioink was printed into custom-made cylindrical molds, with samples of 4 mm in height. After printing, the gel sample molds were placed in a cooled centrifuge to remove any bubbles in the sample, ensuring more accurate mechanical measurements, and then placed in an incubator at a temperature of 37 °C for approximately 15 and 60 min for the samples to solidify. The compression moduli of the samples were measured using a custom-built compression test device. This device utilizes a micrometric linear actuator controlled by a stepper motor and strain gauge strain sensor HT sensor TAS501N (HT Sensor Technology Co., Ltd., Xi’an City, China). The measuring speed was set to 0.1 mm/s. The Dinolite USB microscope (Dino-Lite Digital Microscope, AnMo Electronics Co., Taipei, Taiwan), perpendicular to the sample, was attached to visualize the dimensional change. Measured force/compression depth data were transformed into stress/strain curves. Real stress and strain were estimated using changing geometry from microscope data. Compression modulus was estimated as a linear part at 0.3–0.4 strain region. The entire process is imaged in Figure 18. 

### 4.4. Cell Culture, Bioprinting, and Sample Culture

Four groups of samples were printed, varying in collagen concentration and cultivation method: 20 mg/mL and 30 mg/mL, static cultivation, and active medium perfusion (‘dynamic’ cultivation). The porcine stromal cells used in this article were isolated from porcine neck adipose tissue. Cells were cultured in the growth culture medium DMEM:F12 (Dulbecco’s modified Eagle Medium and Ham’s F-12 Medium, Gibco, Grand Island, NY, USA; supplemented with 2.438 g/L sodium bicarbonate) with 10% fetal bovine serum (FBS, Gibco, Grand Island, NY, USA), 1% ABAM antibiotics (100 IU/mL penicillin, 100 µg/mL of streptomycin, and 0.25 µg/mL of Gibco Amphotericin B; Sigma-Aldrich, St. Louis, MO, USA), and 10 ng/mL FGF2, and passaged with 10% Tryple/mM EDTA in DPBS (all Gibco, Grand Island, NY, USA). Cells were harvested in the fourth passage to prepare the suspension and resuspended in a 2× enhanced culture medium. The final cell density was 13 mil. cells/1 mL of bioink. This density was verified by the digestion of the printed collagen sample with collagenase 1. After digestion, the remaining supernatant was centrifuged. The pellet was resuspended in a PBS solution, fixed using 70% ethanol, and stained with DAPI. The cell nuclei were then counted in the cytometer cell. 

Sample printing was performed using a modified 3D bioprinter based on Cellink Inkredible+ (CELLINK/Bico Group, Göteborg, Sweden). The 14 × 14 × 1.2 mm and 14 × 14 × 0.8 mm squares were printed on tissue-treated coverslip glass using a G17 nozzle (HotAIR, Ostrava, Czech Republic). The feed rate was set at 2000 mm/min; the print head temperature was <10 °C, and the platform temperature was maintained at 37 °C. 

After the gel had solidified (approximately 3 min in the air), the samples were flooded with culture medium and placed in an incubator for 20 min for the final stiffening. The samples were then peeled off the coverslips and placed in perfusion system cups (for active perfusion of the medium—in more detail described below) or 6-well culture plates (for static samples). Using custom-built pneumatically driven membrane pumps and membrane valves, the active perfusion system, as shown in Figure 14, ensured continuous culture medium replacement by gently pulsing the medium to create a medium flow around the sample. This method was compared to conventional static culture, where samples were cultured without active perfusion and with manual medium changes every 72 h. The samples were grown for 1, 3, 5, and 7 days of cultivation. Each culture was then microscopically evaluated (cell viability, morphology) using the live-dead assay protocol and SEM. 

As culture medium, we used a proliferation growth culture medium (DMEM:F12, 10% FBS, 1% ABAM and 10 ng/mL FGF2) for a batch and a differential culture medium for smooth muscle cells (SMC) DMEM: F12 with 10% FBS, 1% ABAM, 50 μg/mL ascorbic acid, 2.5 ng/mL TGF-β1 (Transformation growth factor β), and 2.5 ng/mL BMP4 (Bone morphological protein 4) for another batch. 

Active media perfusion was realized in our custom-built pneumatic perfusion and stimulation system with a unique cultivation chamber. According to our previous studies, dynamic cultivation promoted cell proliferation, differentiation, and recolonization of decellularized substrates. This was achieved by combining pulsatile perfusion with controlled flow and pressure change [32,33].

Due to different sample types and relatively high cell density, the cultivation setup was modified. First, we have designed a special cultivation chamber allowing printed samples to float freely in a perfusing medium, enabling optimal diffusion of nutrients from all sides of the sample. To achieve this, a chamber with sample baskets was created. We have made these sample baskets using a 3D SLA print from biocompatible material (Formlabs Biomed Clear, Formlabs, Somerville, MA, USA). These baskets have an inner space of 22 × 22 × 8 mm with regularly spaced lamellae (lamella size 1.2 mm with 1.2 mm spacing). These baskets with the samples are fixed in a polycarbonate-based chamber with inlet and outlet fluidic ports, allowing medium flow. During cultivation, the chambers with samples are vertically oriented. This allows samples to float freely in a prefunding medium and also minimizes the formation of unwanted bubbles. Each chamber can hold two baskets, or the extended version can hold up to four baskets with samples. These chambers can be connected in series to cultivate more samples, as was used in our design (one chamber for one cultivation interval). The cultivation chamber cross-section with perfusion scheme and sample floating in prefunding medium is illustrated in Figure 8.

**Figure 19:**
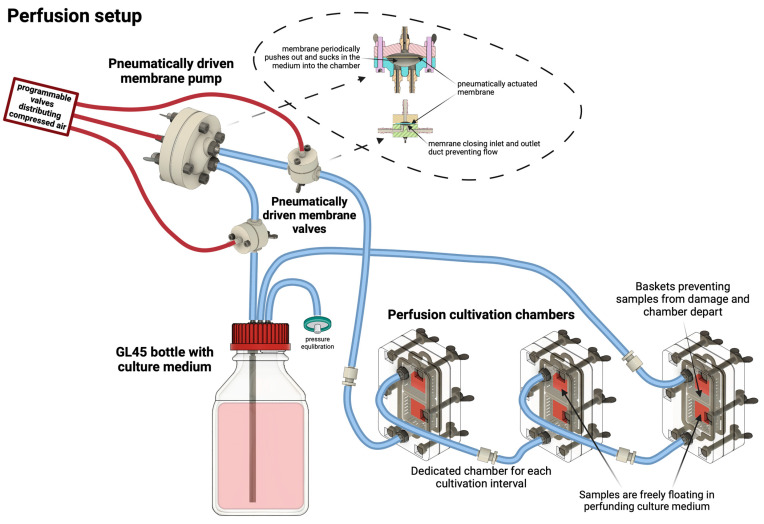
Active perfusion system compounded of a system of reservoir, pump, valves, and culture chambers, with samples freely floating in prefunding culture medium.

Perfusion was established using a pneumatic-based membrane pump. In our published studies, we used linearly actuated syringe pumps; however, they have limitations in overall volume. Due to the high cell density in samples, we had to utilize larger volumes of culture media; thus, a pneumatic system was used. The pumping of the medium is realized by a membrane pump together with two membrane valves connected to the system, distributing compressed air to these components. The membrane pump has a 3 mL chamber from which the medium is periodically ejected into the perfusion circuit. Membrane valves are repeatedly opened and closed, allowing the pushing media from the membrane pump into a circuit with connected chambers and refiling the pump chamber with culture media, generating pulsatile flow. For these experiments, we utilized a 4 s ejection phase with a 6 s refilling, giving us 15 mL/min perfusion. This relatively slow ejection and refilling phase was set to minimize pressure pulses that can stimulate cells [32] for differentiation. Pressure pulses were kept below 5 mmHg (0.6 kPa). The perfusion setup is illustrated in Figure 19.

### 4.5. Cell Viability

To determine cell viability, live cell staining was performed with fluorescein diacetate (FDA, 5 mg/mL; F1303, ThermoFisher, Waltham, MA, USA), and dead cell staining was performed with propidium iodide (PI, 2 mg/mL; P1304MP, ThermoFisher) after 1, 3, and 5 days of culture. First, cells were washed with PBS and then incubated with FDA and PI, which were diluted in a culture medium without fetal bovine serum for seven minutes at room temperature. The stained cells are then observed under a custom-built Thorlabs CERNA wide-field fluorescence microscope equipped with Olympus Plan Fluorite 10× objectives and a CCD camera from The Imaging Source, with live cells fluorescing green due to FDA uptake, and dead cells fluorescing red due to PI uptake. 

### 4.6. Morphology

Filamentous actin (F-actin) was visualized in the cell cytoskeleton using Alexa Fluor^TM^ 594 phalloidin (Thermo-Fisher, Waltham, MA, USA, cat. no. A12381, 200 U/mL) and cell nuclei using DAPI (Thermo-Fisher, D1306, concentration 300 nM). As an early marker of SMC differentiation, we used Alexa Fluor™ 488 (Thermo-Fisher, Waltham, MA, USA, concentration 2 μg/mL). The samples were fixated using Palay solution for approximately one hour, then permeabilized using 0.1% Triton-X 100 in PBS for 10 min and blocked using blocking solution (1% BSA, 22.52 mg/mL glycine in PBST (PBS + 0.1% Tween 20)) for 25 min. Staining was performed in humidified chambers at room temperature for 120 min, after which the samples were rinsed several times and stored in PBS.

Confocal microscopy was performed using a Nikon CSU-W1 inverted spinning disk microscope, which was based on a Nikon Eclipse Ti2 inverted microscope (Nikon, Tokyo, Japan) with the Yokogawa CSU-W1 spinning disc module (Yokogawa, Tokyo, Japan) and equipped with dual sCMOS PRIME BSI cameras (Teledyne Photometrics, Tucson, AZ, USA). Nikon CFI Plan Apo VC 20× dry lenses with a 25 mm pinhole disc were used in Z stack mode (depth −0–250 mm) to image the samples. The images were captured and subsequently processed using the Imaris 10.1.0 software (Oxford Instruments, Abingdon-on-Thames, UK).

### 4.7. MTT Assay

Cells were also tested with an MTT assay to measure cellular metabolic activity as an indicator of cell viability, proliferation, and cytotoxicity. After each cultivation period, a mixture of MTT labeling reagent (0.25 mg/mL MTT in DPBS, Sigma-Aldrich, St. Louis, MO, USA) and growth culture medium (DMEM:F12 with 10% FBS, 1% ABAM, and 10 ng/mL FGF2) in a ratio of 1:10 was added to the samples. After 1 h of cultivation in a humidified chamber at 37 °C, the MTT labeling reagent was drained, and 500 μL of solubilization solution (10% Triton X-100/0.1 M HCl) was added and placed in a vortex in a humidified chamber at 37 °C for approximately an hour. After the incubation period, the absorbance of the samples was measured spectrophotometrically using a Biochrom WPA Lightwave II UV/visible spectrophotometer (Biochrom Ltd., Cambridge, UK). The reference wavelength was set to 570 nm. 

### 4.8. SEM

To visualize the morphology of collagenous gels in situ, samples were viewed by scanning electron microscopy (SEM) on the STEM Apreo S2 microscope (Thermo Scientific, Waltham, MA, USA) in high vacuum mode, standard, and OptiPlan use cases. The Everhart-Thornley detector and Inlens Trinity T2 detector were used in secondary electron mode at 2–10 keV with a magnification of 1000×–100,000×. Collagen hydrogels were fixed in fixative solution (preparation of this solution preparation is given in the Supplementary file) for 2 h at room temperature followed by overnight fixation at 4 °C, further processed by multiply washing in phosphate buffer solution supplemented with glucose and then graded series of ethanol and acetone dehydration in Leica EM TP tissue processor (Specion sro., Praha, CZ), dried on a Leica EM CPD300 critical point dryer. The dried samples were mounted on stubs using carbon adhesive stickers and sputter coated with Pt in an Ar atmosphere in a Leica EM ACE600 coating system (the thickness of the Pt layer was 13 nm). 

### 4.9. Statistical Evaluation

The statistical significance of individual variables was tested using an unpaired *t*-test in case the variable was stratified into two classes and ANOVA in case of more classes. Linear regression with stepwise selection was used to assess the overall statistical significance of all variables together. The statistical significance level was set to 0.05. The statistical software used was the SAS 9.4 TS Level 1M7 X64_10PRO platform. More detailed results are available in the supplementary materials. Boxplots in figures were created using Python with the Seaborn module.

## Figures and Tables

**Table 1:** Linear regression parameters table for collagen concentration 20 mg/mL.

Variable	Parameter Estimate	Standard Error	*p*-Value
Intercept	11.44946	2.76723	0.0002
AA concentration	−4.90977	1.49797	0.0024
pH optimization	4.58115	1.49797	0.0043
Incubation time	4.14039	0.49786	<0.0001

**Table 2:** Linear regression parameters table for collagen concentration 30 mg/mL.

Variable	Parameter Estimate	Standard Error	*p*-Value
Intercept	41.84674	5.16653	<0.0001
AA concentration	−16.31885	2.82094	<0.0001
pH optimization	9.99045	2.82094	0.0011
Incubation time	12.07068	0.94031	<0.0001

**Table 3:** Linear regression parameters table for substrate sample area change by different concentrations of collagen, days of cultivation, and cultivation types.

Variable	Parameter Estimate	Standard Error	*p*-Value
Intercept	81.146	17.348	<0.0001
Days of cultivation	−8.387	1.120	<0.0001
Concentration	5.665	0.573	<0.0001
Cultivation (1 static, 2 active perfusion)	−32.959	5.738	<0.0001

**Table 4:** Linear regression parameters table for substrate sample thickness change by different concentrations of collagen, days of cultivation, and cultivation types.

Variable	Parameter Estimate	Standard Error	*p*-Value
Intercept	1.151	0.036	<0.0001
Days of cultivation	−0.027	0.002	<0.0001
Concentration	0.006	0.001	<0.0001
Cultivation (1 static, 2 active perfusion)	−0.079	0.012	<0.0001

**Table 5:** Linear regression parameters table for substrate sample area change by different concentrations of collagen, days of cultivation, initial print heights, and cultivation types.

Variable	Parameter Estimate	Standard Error	*p*-Value
Intercept	105.595	15.321	<0.0001
Days of cultivation	−31.000	1.525	<0.0001
Initial print height	Not entering the model (has minimal effect)		
Concentration	2.847	0.585	<0.0001
Cultivation (1 static and 2 active perfusion)	Not entering the model (has minimal effect)		

**Table 6:** Linear regression parameters table for substrate sample thickness change by different concentrations of collagen, days of cultivation, initial print heights, and cultivation types.

Variable	Parameter Estimate	Standard Error	*p*-Value
Intercept	0.054	0.038	0.016
Days of cultivation	−0.014	0.003	<0.0001
Concentration	Not entering the model (has minimal effect)		
Cultivation (−1 static and 1 active perfusion)	−0.088	0.007	<0.0001
Initial print height	0.962 (obvious role)	0.036	0.002

## Data Availability

Data are contained within the article.

## Supplementary Materials

The following supporting information can be downloaded at: https://www.mdpi.com/article/10.3390/gels10050316/s1, Supplementary files contains statistical evaluation of measured data.

## Appendix A

### Preparation of Fixative Solution for SEM

Paraformaldehyde weighing 2.5 g was dissolved in 100 mL of distilled water while stirring and heating to 60 °C. After reaching the temperature, 1M NaOH was gradually added until the solution became clear. After cooling to room temperature, components: 75 mL of 0.4 M phosphate buffer, 10 mL of 25% glutaraldehyde, 0.75 mL of 0.5 wt.% CaCl_2_ were added. Distilled water was added to reach a final volume of 250 mL, and then the mixture was mixed and filtered. Finally, the pH value of the final solution was measured, and 1M NaOH was used to change (when necessary) and stabilize pH at a value of 7.2–7.5.

## Appendix B

### Table for Determining the Ideal Ratio for the First Neutralization of Collagen Hydrogel

The ideal addition of a 2× enhanced culture medium with the addition of 25 μL/mL 1M NaOH can be determined using a titration curve presented in Figure 17. For that, we prepared a simple table for selecting the ideal ratio for the first neutralization of collagen, i.e., if the ideal NaOH addition (μL/mL) is approximately 7, the closest number in the table is 7.14, which suggests that the perfect ratio is 5:2 (2× enhanced culture medium: 2× enhanced culture medium with NaOH addition), see Table A1.

**Table A1:** Table for determining the ideal ratio for the first neutralization of collagen hydrogel.

C1	0	μL/mL NaOH									
C2	25	μL/mL NaOH									
	0	1	2	3	4	5	6	7	8	9	10
0		0.00	0.00	0.00	0.00	0.00	0.00	0.00	0.00	0.00	0.00
1	25.00	12.50	8.33	6.25	5.00	4.17	3.57	3.13	2.78	2.50	2.27
2	25.00	16.67	12.50	10.00	8.33	7.14	6.25	5.56	5.00	4.55	4.17
3	25.00	18.75	15.00	12.50	10.71	9.38	8.33	7.50	6.82	6.25	5.77
4	25.00	20.00	16.67	14.29	12.50	11.11	10.00	9.09	8.33	7.69	7.14
5	25.00	20.83	17.86	15.63	13.89	12.50	11.36	10.42	9.62	8.93	8.33
6	25.00	21.43	18.75	16.67	15.00	13.64	12.50	11.54	10.71	10.00	9.38
7	25.00	21.88	19.44	17.50	15.91	14.58	13.46	12.50	11.67	10.94	10.29
8	25.00	22.22	20.00	18.18	16.67	15.38	14.29	13.33	12.50	11.76	11.11
9	25.00	22.50	20.45	18.75	17.31	16.07	15.00	14.06	13.24	12.50	11.84
10	25.00	22.73	20.83	19.23	17.86	16.67	15.63	14.71	13.89	13.16	12.50
