# Supplementary material for: Active Media Perfusion in Bioprinted Highly Concentrated Collagen Bioink Enhances the Viability of Cell Culture and Substrate Remodeling
Source: Gels. 2024 May 5;10(5):316. doi: 10.3390/gels10050316 (PMC11120981; doi:10.3390/gels10050316)
Supplement: Supplementary file 1 [file gels-10-00316-s001.zip › gels-2990637-supplementary.pdf]

Biomechanical evaluation
Test between colagen concentration

Variable: E_03_04 (Compression modulus (kPa))

Koncentrace_kolagen	Method	N	Mean	Std Dev	Std Err	Minimum	Maximum
**20**		38	16.7350	8.4424	1.3695	6.1239	33.5466
**30**		39	53.3419	22.0537	3.5314	19.8817	92.3457
**Diff (1-2)**	**Pooled**		-36.6069	16.7806	3.8250		
**Diff (1-2)**	**Satterthwaite**		-36.6069		3.7877		

Koncentrace_kolagen	Method	Mean	95% CL Mean		Std Dev	95% CL Std Dev	
**20**		16.7350	13.9601	19.5099	8.4424	6.8828	10.9223
**30**		53.3419	46.1930	60.4909	22.0537	18.0233	28.4223
**Diff (1-2)**	**Pooled**	-36.6069	-44.2267	-28.9872	16.7806	14.4718	19.9727
**Diff (1-2)**	**Satterthwaite**	-36.6069	-44.2180	-28.9959			

Method	Variances	DF	t Value	Pr > |t|
**Pooled**	Equal	75	-9.57	<.0001
**Satterthwaite**	Unequal	49.148	-9.66	<.0001

Equality of Variances				
**Method**	**Num DF**	**Den DF**	**F Value**	**Pr > F**
**Folded F**	38	37	6.82	<.0001

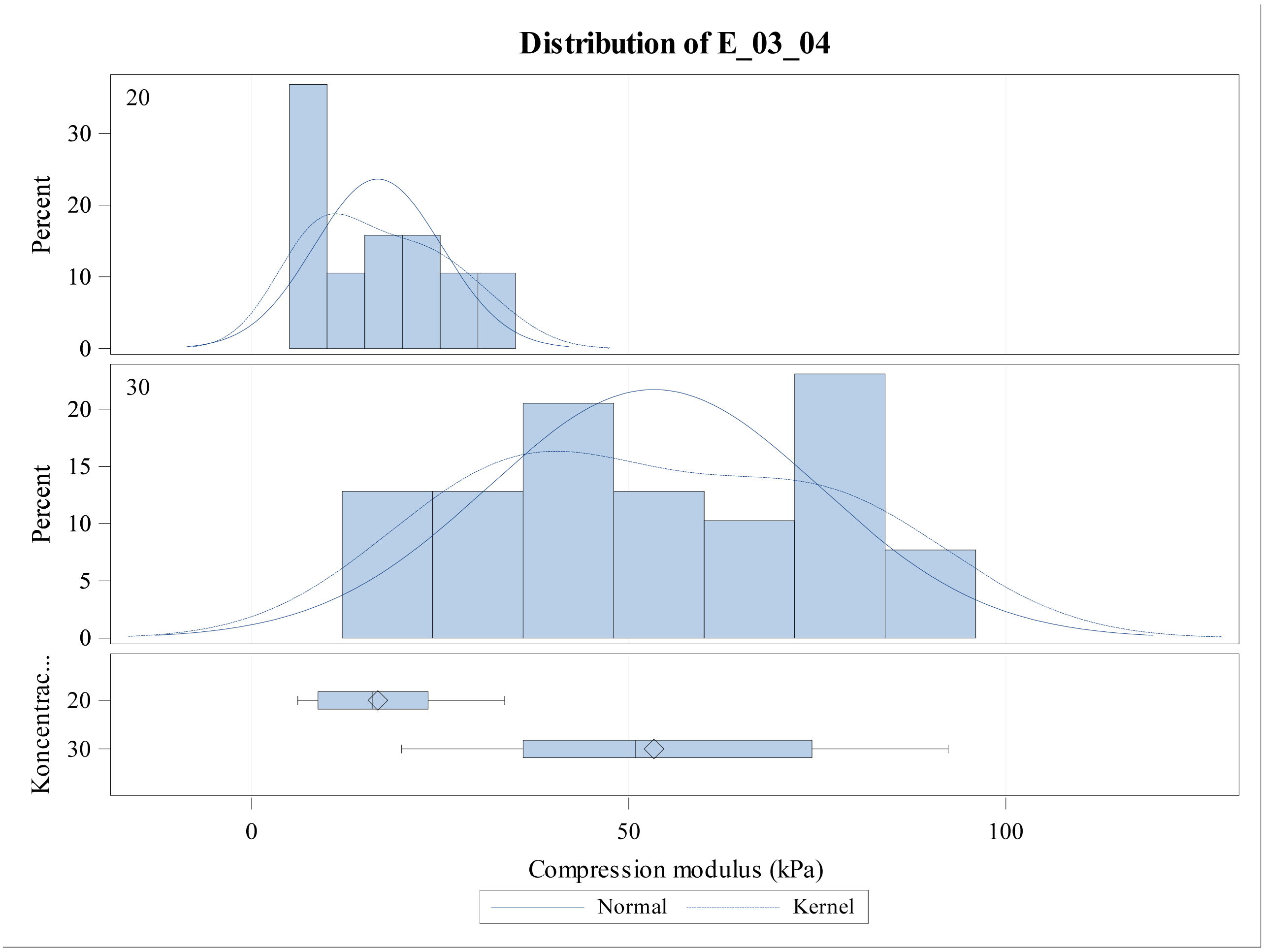
Distribution of E_03_04
20
30
20
10
0
Percent
30
20
15
10
5
0
Percent
Koncentrac...
20
30
0
50
100
Compression modulus (kPa)
Normal
Kernel

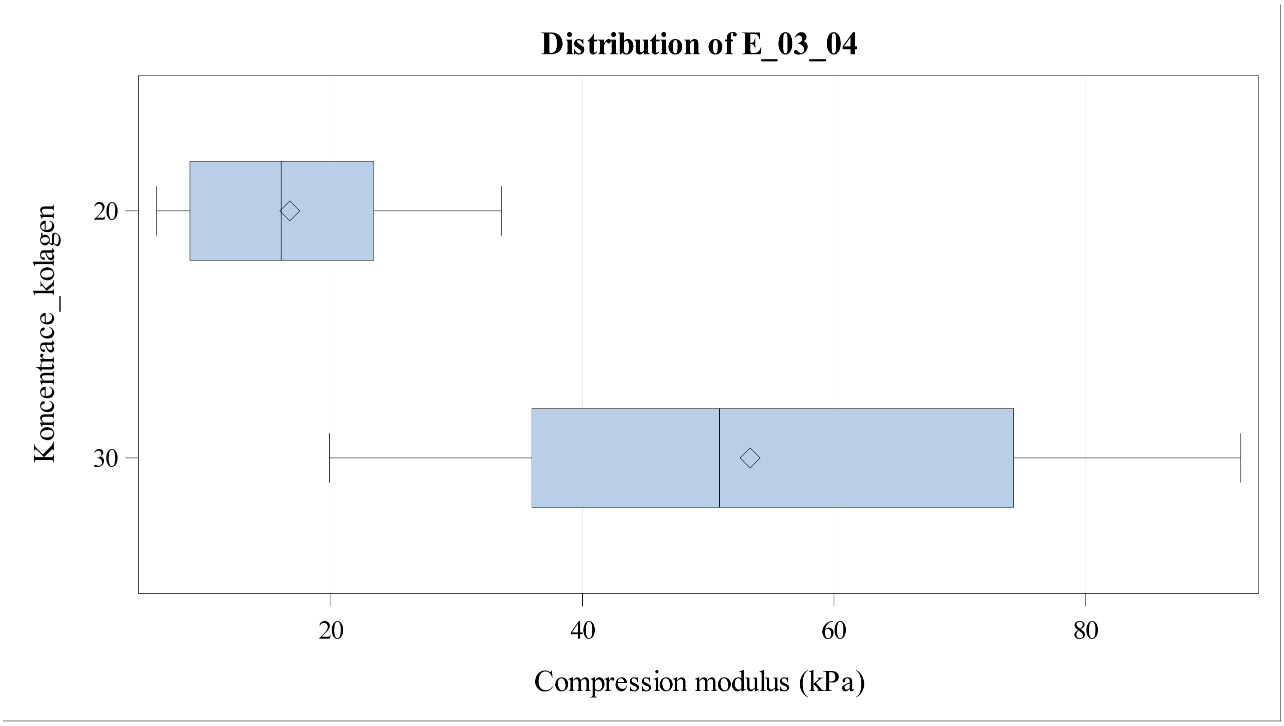
Distribution of E_03_04
Koncentrace_kolagen
20
30
20
40
60
80
Compression modulus (kPa)

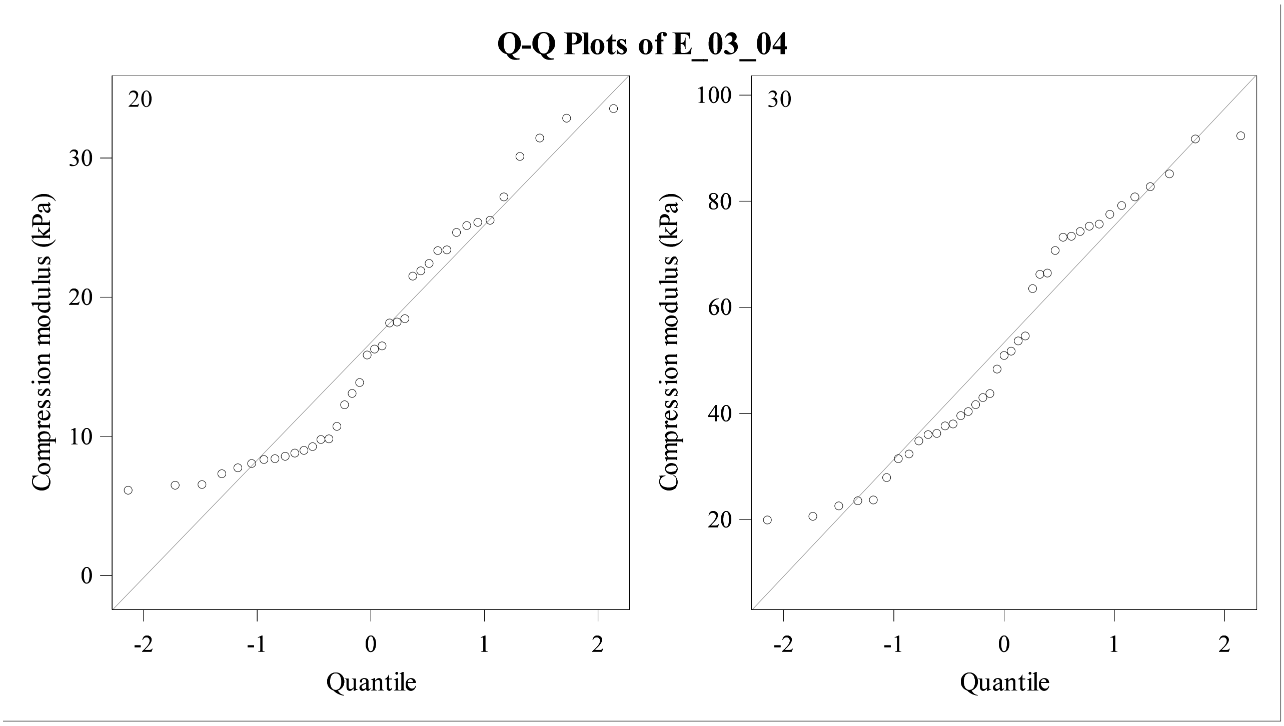

Biomechanical evaluation
Test between colagen concentration

Model: MODEL1
Dependent Variable: E_03_04 Compression modulus (kPa)

Collagen conc. (mg/ml)=20

**Number of Observations Read**	38
**Number of Observations Used**	38

Stepwise Selection: Step 1

Variable inkubace_new Entered: R-Square = 0.5558 and C(p) = 21.2751

Analysis of Variance					
**Source**	**DF**	**Sum of Squares**	**Mean Square**	**F Value**	**Pr > F**
**Model**	1	1465.71023	1465.71023	45.04	<.0001
**Error**	36	1171.41554	32.53932		
**Corrected Total**	37	2637.12577			

Variable	Parameter Estimate	Standard Error	Type II SS	F Value	Pr > F
Intercept	6.38402	1.79858	409.95499	12.60	0.0011
inkubace_new	4.14039	0.61691	1465.71023	45.04	<.0001

Bounds on condition number: 1, 1

Stepwise Selection: Step 2

Variable koncentrace_aa_new Entered: R-Square = 0.6516 and C(p) = 11.3529

Analysis of Variance					
Source	DF	Sum of Squares	Mean Square	F Value	Pr > F
Model	2	1718.37127	859.18564	32.73	<.0001
Error	35	918.75450	26.25013		
Corrected Total	37	2637.12577			

Variable	Parameter Estimate	Standard Error	Type II SS	F Value	Pr > F
Intercept	13.99454	2.93722	595.90503	22.70	<.0001
koncentrace_aa_new	-5.16428	1.66459	252.66104	9.63	0.0038
inkubace_new	4.14039	0.55409	1465.71023	55.84	<.0001

Bounds on condition number: 1, 4

Stepwise Selection: Step 3

Variable phnum Entered: R-Square = 0.7268 and C(p) = 4.0000

Analysis of Variance					
Source	DF	Sum of Squares	Mean Square	F Value	Pr > F
Model	3	1916.58146	638.86049	30.15	<.0001
Error	34	720.54431	21.19248		
Corrected Total	37	2637.12577			

Variable	Parameter Estimate	Standard Error	Type II SS	F Value	Pr > F
**Intercept**	11.44946	2.76723	362.79389	17.12	0.0002
**koncentrace_aa_new**	-4.90977	1.49797	227.66632	10.74	0.0024
**phnum**	4.58115	1.49797	198.21019	9.35	0.0043
**inkubace_new**	4.14039	0.49786	1465.71023	69.16	<.0001

Bounds on condition number: 1.0031, 9.0186

All variables left in the model are significant at the 0.1500 level.

All variables have been entered into the model.

Summary of Stepwise Selection									
**Step**	**Variable Entered**	**Variable Removed**	**Label**	**Number Vars In**	**Partial R-Square**	**Model R-Square**	**C(p)**	**F Value**	**Pr > F**
**1**	inkubace_new		Incubation time (1: 15 min; 4: 60 min)	1	0.5558	0.5558	21.2751	45.04	<.0001
**2**	koncentrace_aa_new		Acetic acid conc. (1:0.05%; 2:0.1%)	2	0.0958	0.6516	11.3529	9.63	0.0038
**3**	phnum		pH-num	3	0.0752	0.7268	4.0000	9.35	0.0043

Model: MODEL1
Dependent Variable: E_03_04 Compression modulus (kPa)

Collagen conc. (mg/ml)=20

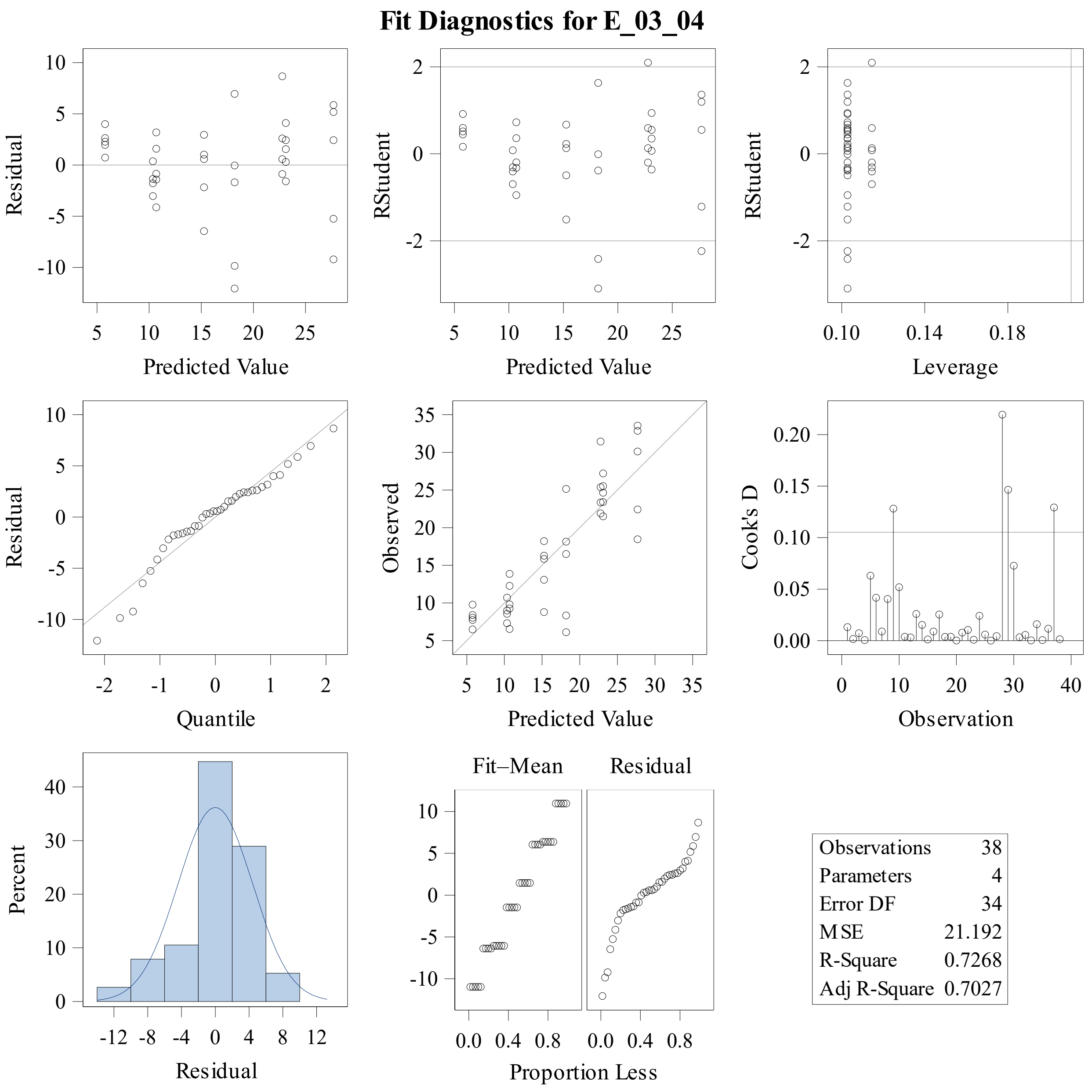

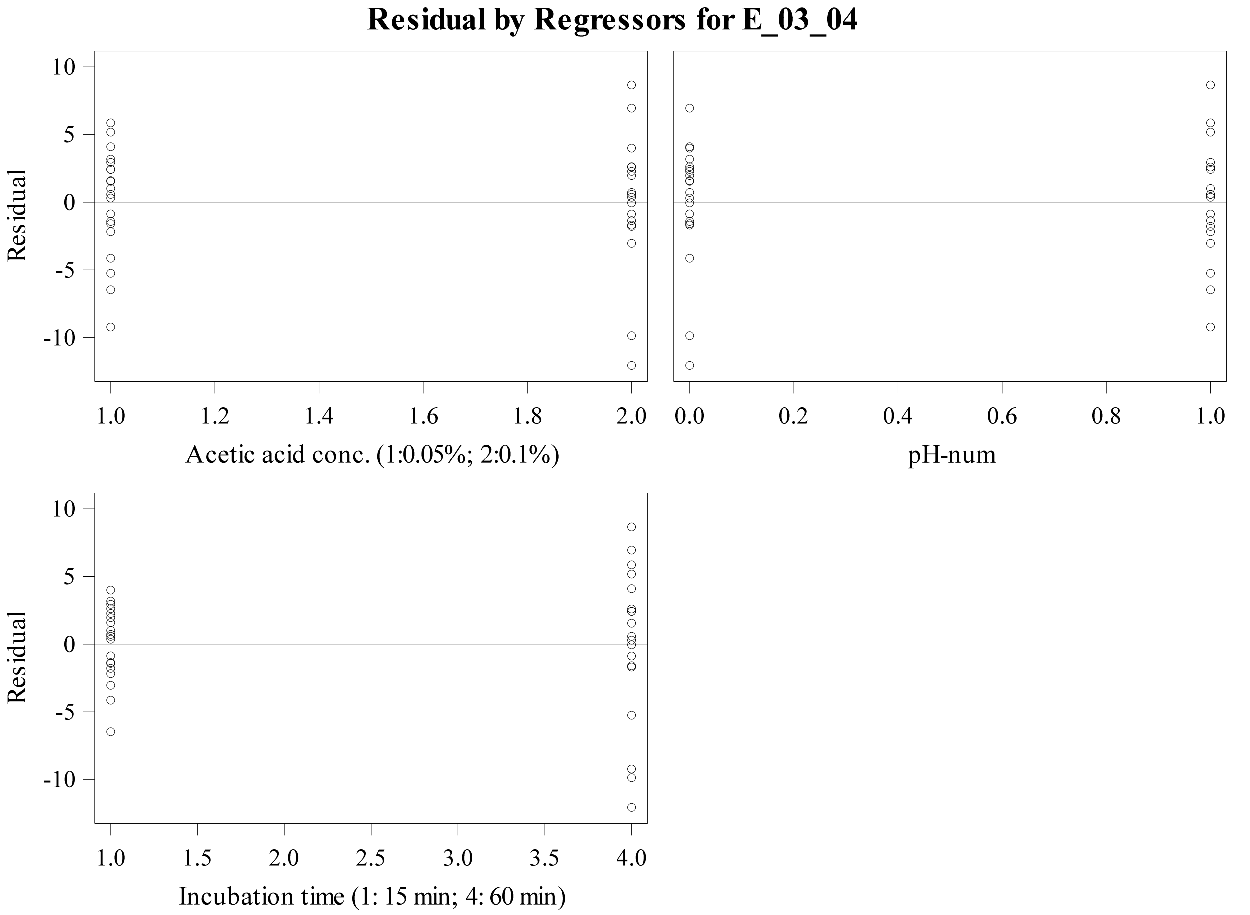

Model: MODEL1
Dependent Variable: E_03_04 Compression modulus (kPa)

Collagen conc. (mg/ml)=30

**Number of Observations Read**	39
**Number of Observations Used**	39

Stepwise Selection: Step 1

Variable inkubace_new Entered: R-Square = 0.6651 and C(p) = 44.9329

Analysis of Variance					
**Source**	**DF**	**Sum of Squares**	**Mean Square**	**F Value**	**Pr > F**
**Model**	1	12293	12293	73.49	<.0001
**Error**	37	6188.91101	167.26787		
**Corrected Total**	38	18482			

Variable	Parameter Estimate	Standard Error	Type II SS	F Value	Pr > F
Intercept	23.28682	4.07186	5470.75045	32.71	<.0001
inkubace_new	11.83989	1.38110	12293	73.49	<.0001

Bounds on condition number: 1, 1

Stepwise Selection: Step 2

Variable koncentrace_aa_new Entered: R-Square = 0.8008 and C(p) = 14.5424

Analysis of Variance					
Source	DF	Sum of Squares	Mean Square	F Value	Pr > F
Model	2	14801	7400.42499	72.38	<.0001
Error	36	3681.03934	102.25109		
Corrected Total	38	18482			

Variable	Parameter Estimate	Standard Error	Type II SS	F Value	Pr > F
Intercept	46.79696	5.71587	6853.90289	67.03	<.0001
koncentrace_aa_new	-16.04884	3.24060	2507.87167	24.53	<.0001
inkubace_new	11.98067	1.08020	12578	123.01	<.0001

Bounds on condition number: 1.0007, 4.0028

Stepwise Selection: Step 3

Variable phnum Entered: R-Square = 0.8534 and C(p) = 4.0000

Analysis of Variance					
Source	DF	Sum of Squares	Mean Square	F Value	Pr > F
Model	3	15772	5257.32208	67.90	<.0001
Error	35	2709.92306	77.42637		
Corrected Total	38	18482			

Variable	Parameter Estimate	Standard Error	Type II SS	F Value	Pr > F
**Intercept**	41.84674	5.16653	5079.43093	65.60	<.0001
**koncentrace_aa_new**	-16.31885	2.82094	2591.07443	33.47	<.0001
**phnum**	9.99045	2.82094	971.11628	12.54	0.0011
**inkubace_new**	12.07068	0.94031	12759	164.78	<.0001

Bounds on condition number: 1.0014, 9.0128

All variables left in the model are significant at the 0.1500 level.

All variables have been entered into the model.

Summary of Stepwise Selection									
**Step**	**Variable Entered**	**Variable Removed**	**Label**	**Number Vars In**	**Partial R-Square**	**Model R-Square**	**C(p)**	**F Value**	**Pr > F**
**1**	inkubace_new		Incubation time (1: 15 min; 4: 60 min)	1	0.6651	0.6651	44.9329	73.49	<.0001
**2**	koncentrace_aa_new		Acetic acid conc. (1:0.05%; 2:0.1%)	2	0.1357	0.8008	14.5424	24.53	<.0001
**3**	phnum		pH-num	3	0.0525	0.8534	4.0000	12.54	0.0011

Model: MODEL1
Dependent Variable: E_03_04 Compression modulus (kPa)

Collagen conc. (mg/ml)=30

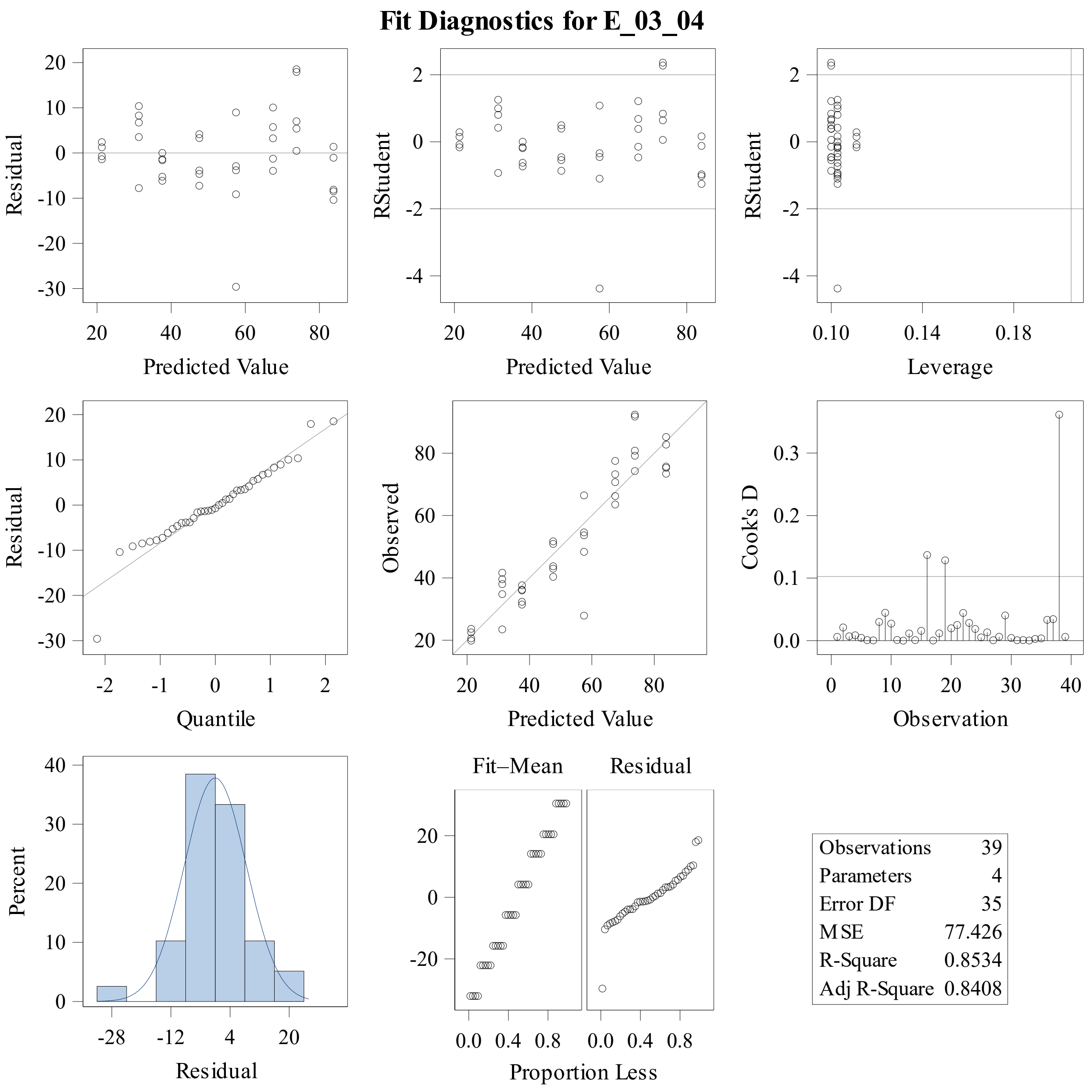

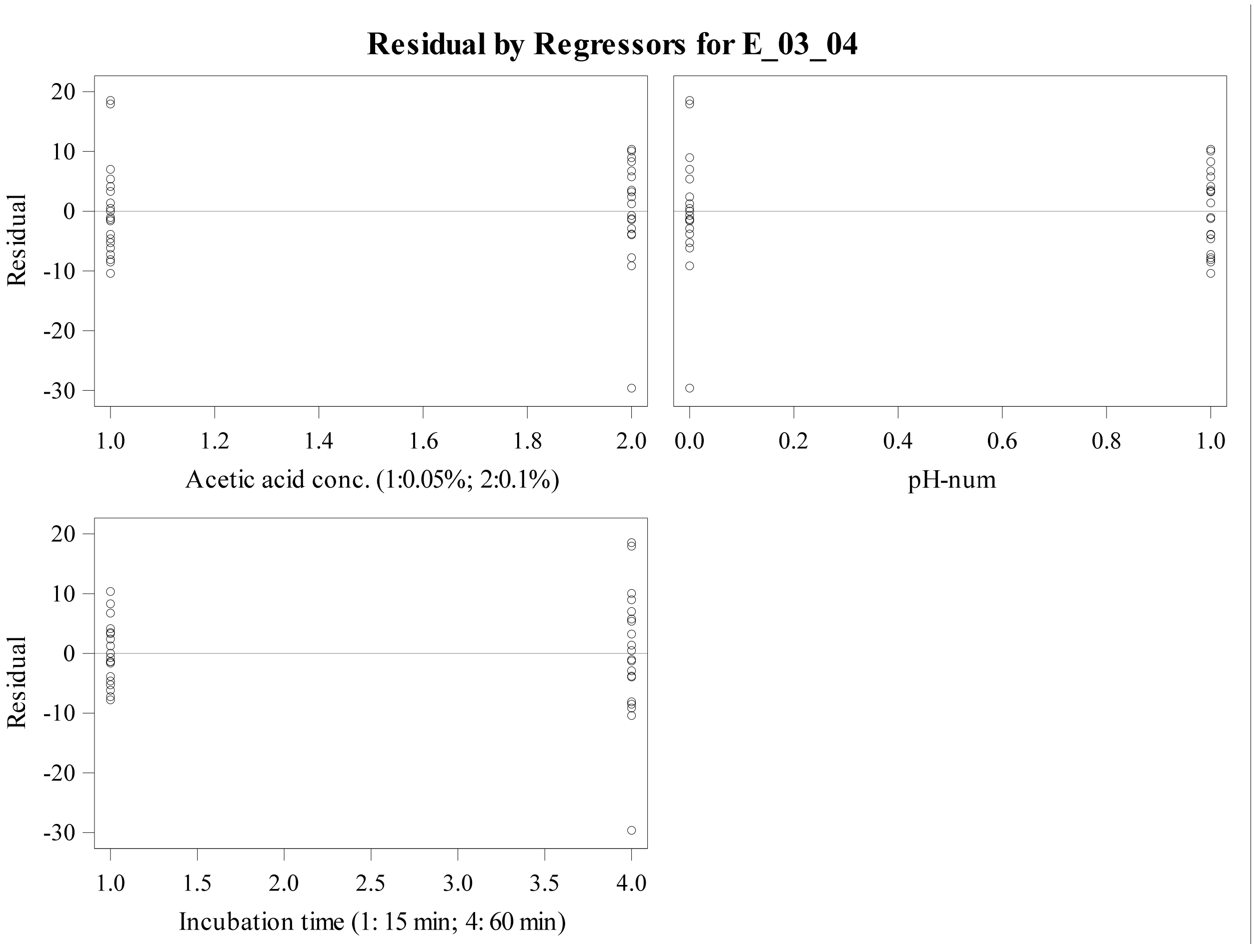

Biomechanical evaluation
Test between colagen concentration

Variable:
Area

Concentration	Method	N	Mean	Std Dev	Std Err	Minimum	Maximum
**20**		40	117.7	46.7145	7.3862	54.8700	201.6
**30**		40	174.8	22.8788	3.6175	125.3	204.5
**Diff (1-2)**	**Pooled**		-57.1453	36.7810	8.2245		
**Diff (1-2)**	**Satterthwaite**		-57.1453		8.2245		

Concentration	Method	Mean	95% CL Mean		Std Dev	95% CL Std Dev	
**20**		117.7	102.7	132.6	46.7145	38.2667	59.9830
**30**		174.8	167.5	182.2	22.8788	18.7414	29.3772
**Diff (1-2)**	**Pooled**	-57.1453	-73.5189	-40.7716	36.7810	31.8050	43.6173
**Diff (1-2)**	**Satterthwaite**	-57.1453	-73.6164	-40.6741			

Method	Variances	DF	t Value	Pr > |t|
**Pooled**	Equal	78	-6.95	<.0001
**Satterthwaite**	Unequal	56.691	-6.95	<.0001

Equality of Variances				
**Method**	**Num DF**	**Den DF**	**F Value**	**Pr > F**
**Folded F**	39	39	4.17	<.0001

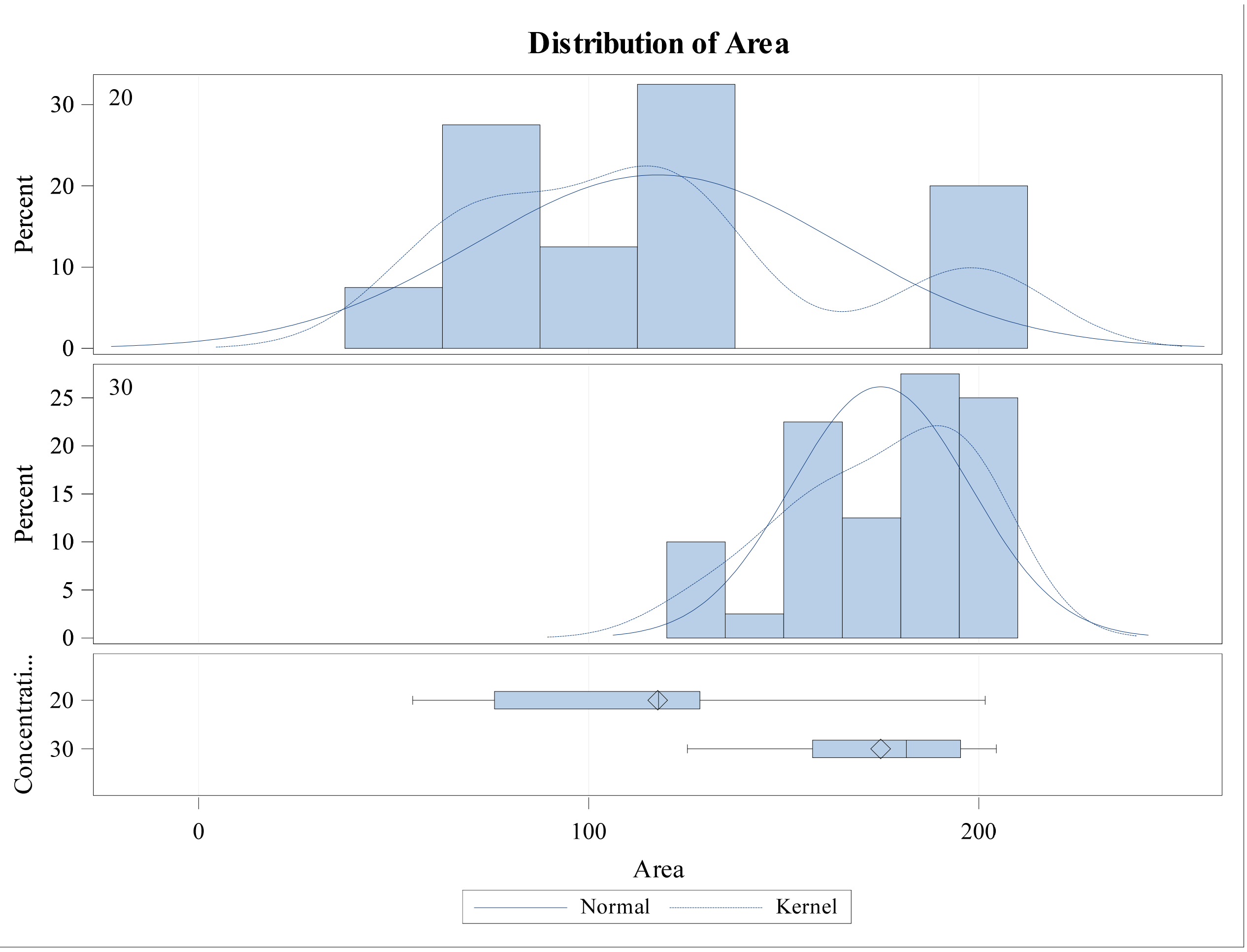

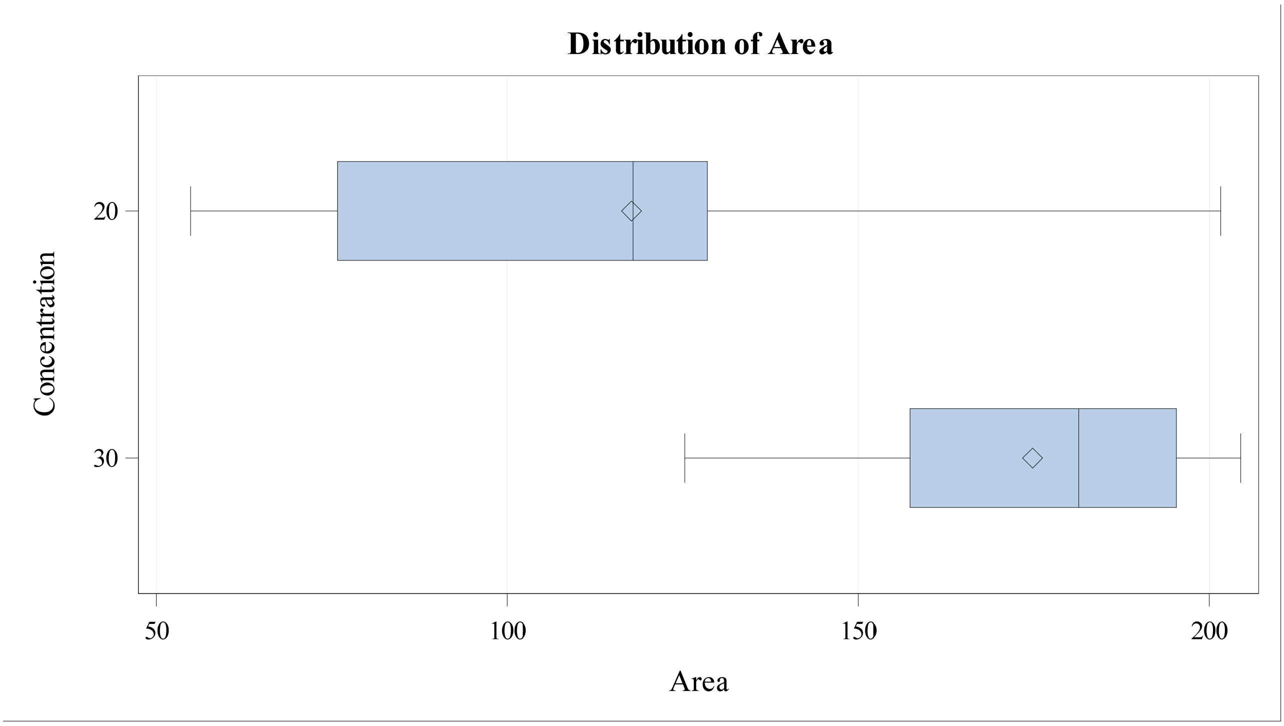
Distribution of Area
Concentration
20
30
50
100
150
200
Area

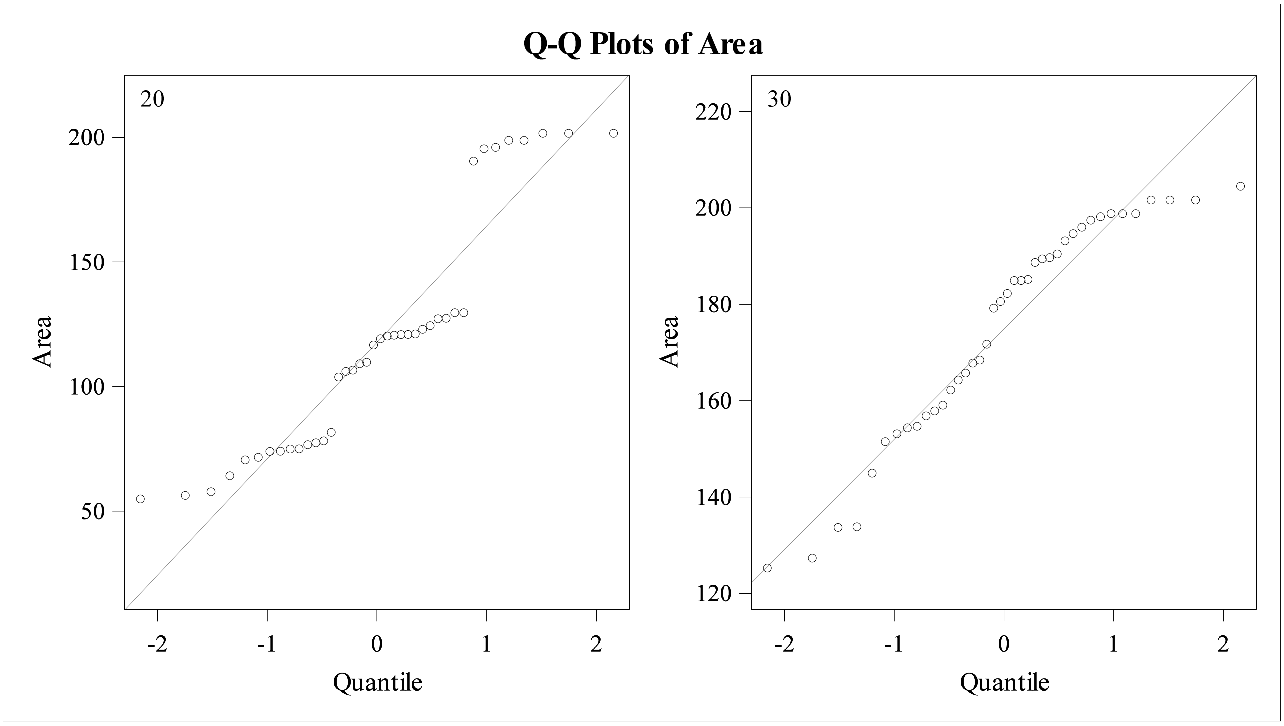
Q-Q Plots of Area
20
Area
200
150
100
50
-2
-1
0
1
2
Quantile
30
Area
220
200
180
160
140
120
-2
-1
0
1
2
Quantile

Biomechanical evaluation
Test between colagen concentration

Variable:
Height

Concentration	Method	N	Mean	Std Dev	Std Err	Minimum	Maximum
**20**		40	1.0740	0.1201	0.0190	0.8100	1.2400
**30**		40	1.1388	0.0677	0.0107	0.9500	1.2400
**Diff (1-2)**	**Pooled**		-0.0647	0.0975	0.0218		
**Diff (1-2)**	**Satterthwaite**		-0.0647		0.0218		

Concentration	Method	Mean	95% CL Mean		Std Dev	95% CL Std Dev	
**20**		1.0740	1.0356	1.1124	0.1201	0.0984	0.1542
**30**		1.1388	1.1171	1.1604	0.0677	0.0554	0.0869
**Diff (1-2)**	**Pooled**	-0.0647	-0.1081	-0.0214	0.0975	0.0843	0.1156
**Diff (1-2)**	**Satterthwaite**	-0.0647	-0.1083	-0.0212			

Method	Variances	DF	t Value	Pr > |t|
**Pooled**	Equal	78	-2.97	0.0039
**Satterthwaite**	Unequal	61.5	-2.97	0.0042

Equality of Variances				
**Method**	**Num DF**	**Den DF**	**F Value**	**Pr > F**
**Folded F**	39	39	3.15	0.0005

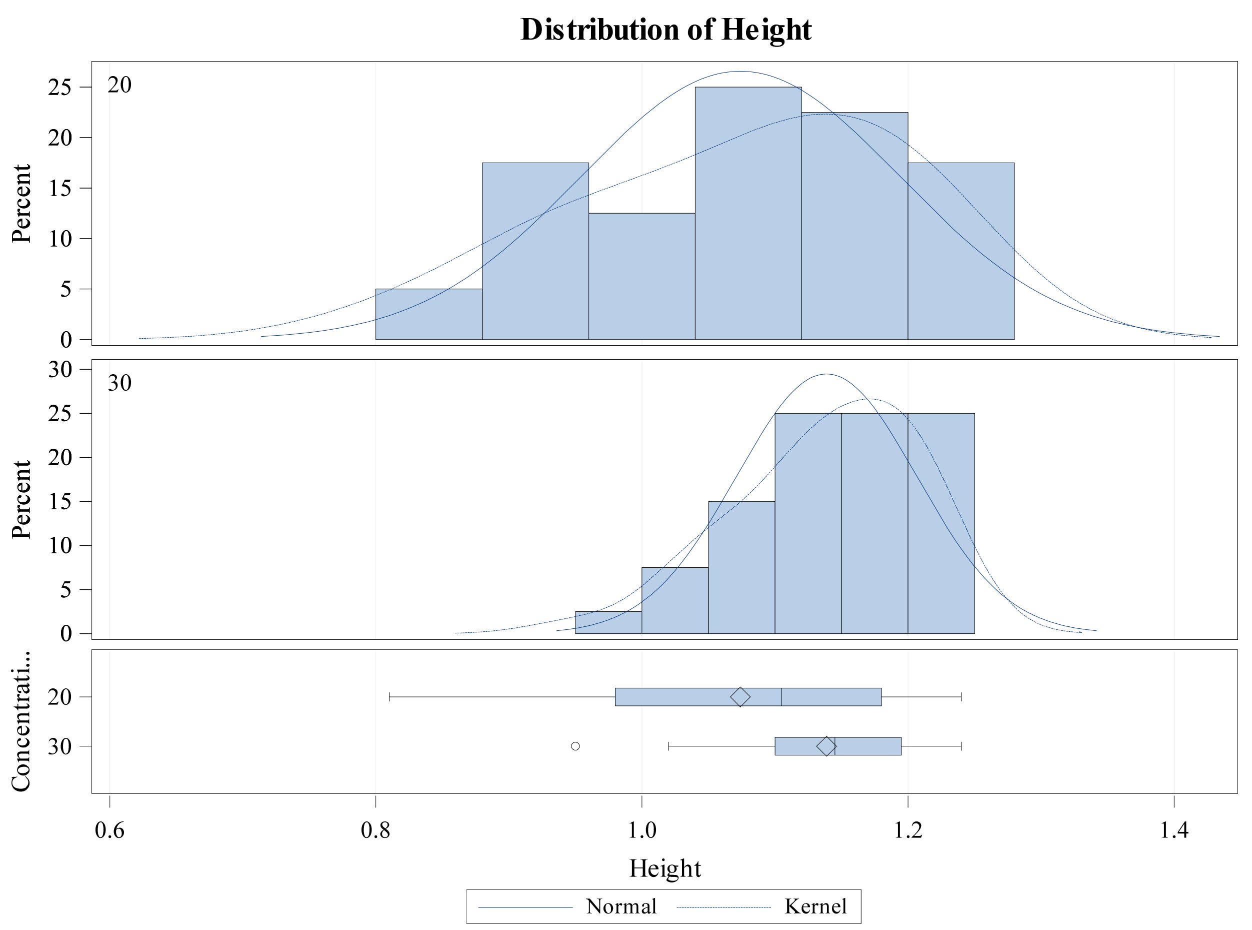
Distribution of Height
20
30
Percent
Concentrati...
0.6
0.8
1.0
1.2
1.4
Height
Normal
Kernel

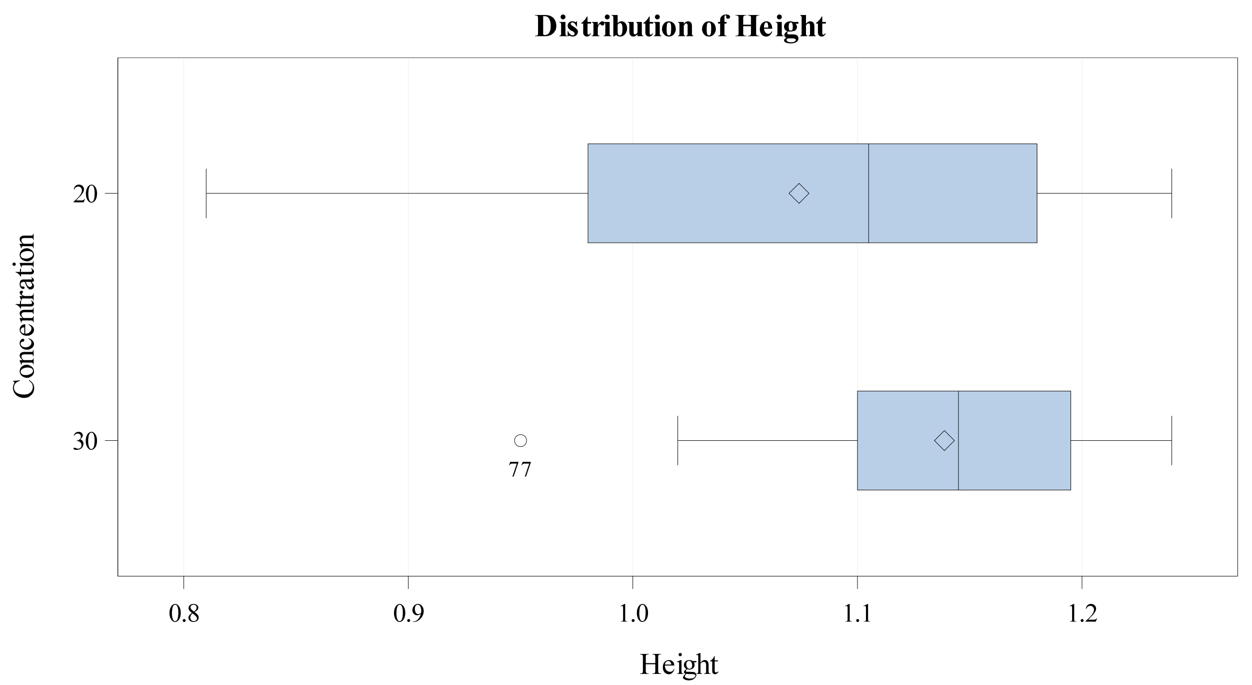
Distribution of Height
Concentration
20
30
77
0.8
0.9
1.0
1.1
1.2
Height

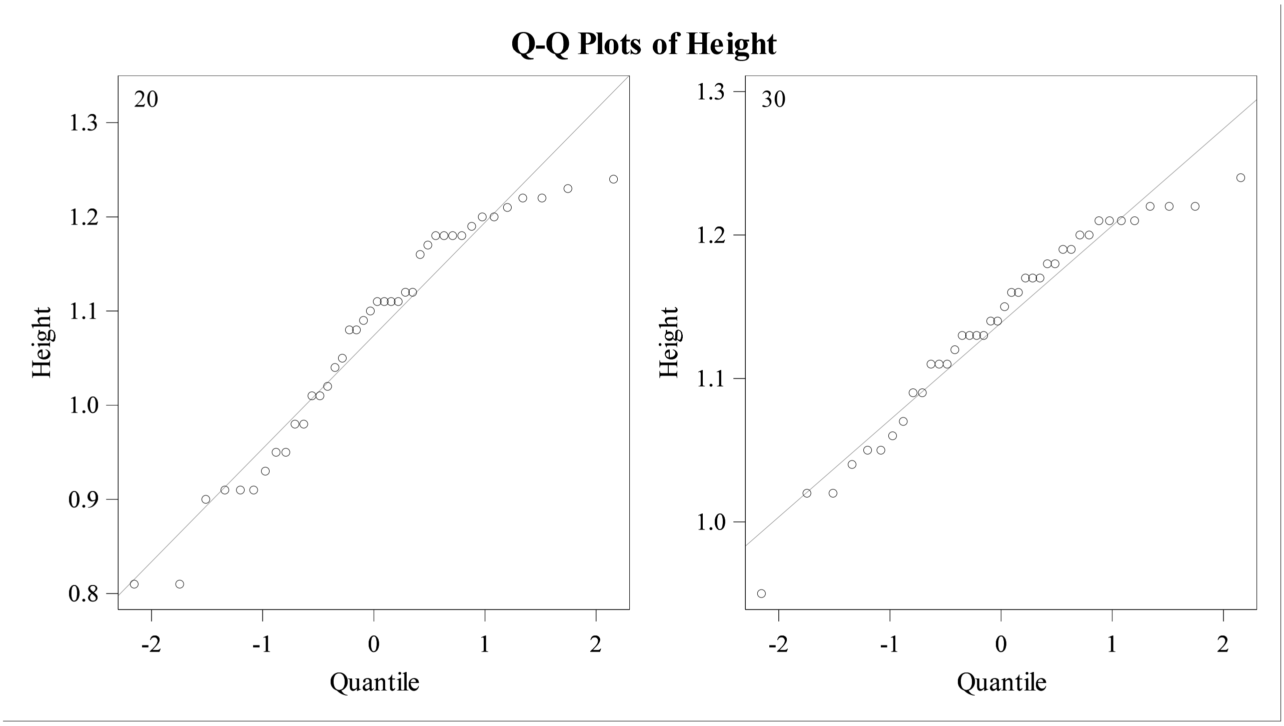

Biomechanical evaluation
Test between colagen concentration

Concentration=20

Class Level Information		
**Class**	**Levels**	**Values**
**Day**	5	0 1 3 5 7

**Number of Observations Read**	40
**Number of Observations Used**	40

Dependent Variable: Area

Concentration=20

Source	DF	Sum of Squares	Mean Square	F Value	Pr > F
**Model**	4	65492.61153	16373.15288	29.22	<.0001
**Error**	35	19614.80616	560.42303		
**Corrected Total**	39	85107.41770			

R-Square	Coeff Var	Root MSE	Area Mean
0.769529	20.11497	23.67326	117.6898

Source	DF	Anova SS	Mean Square	F Value	Pr > F
Day	4	65492.61153	16373.15288	29.22	<.0001

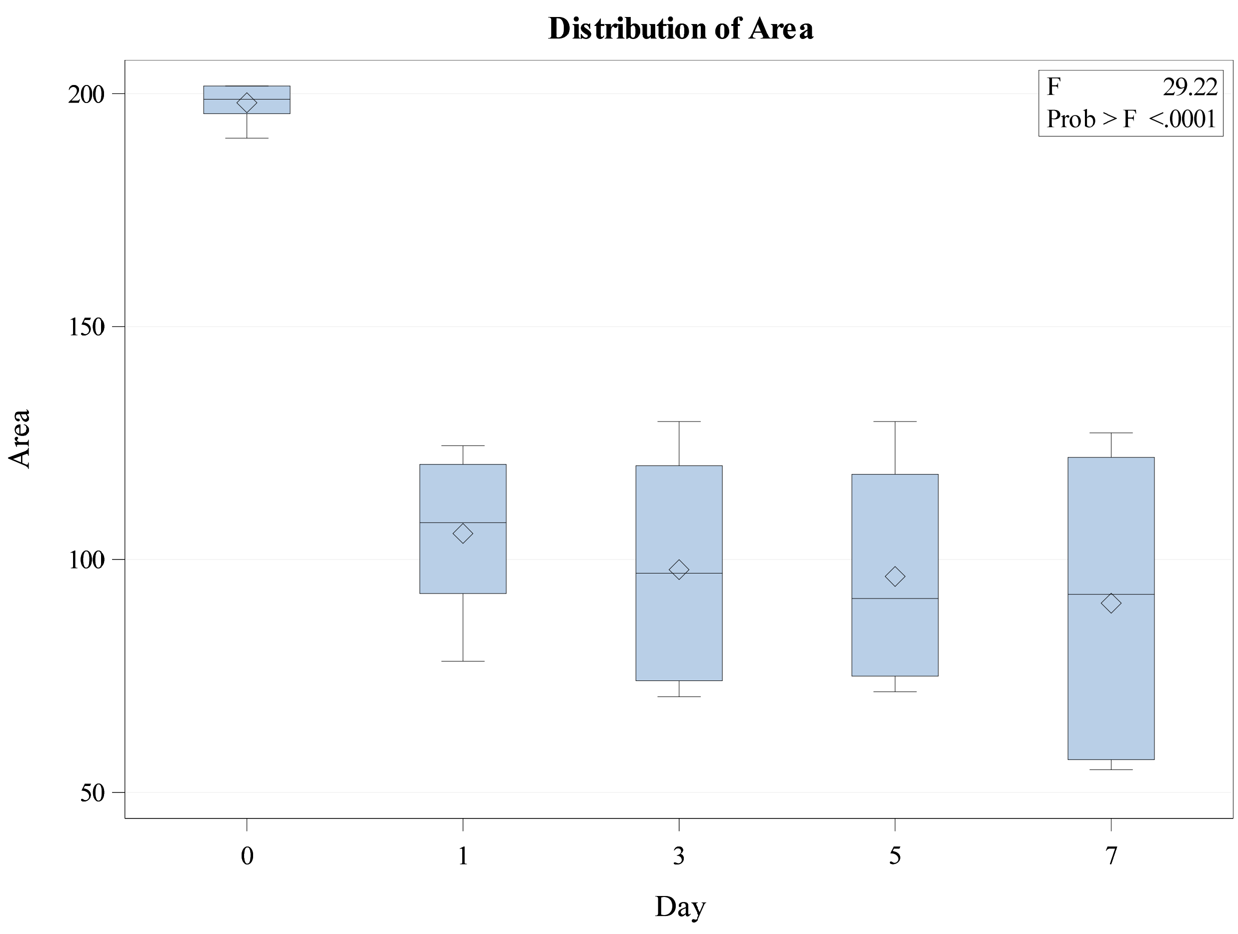

Concentration=30

Class Level Information		
Class	Levels	Values
Day	5	0 1 3 5 7

Number of Observations Read	40
Number of Observations Used	40

Dependent Variable: Area

Concentration=30

Source	DF	Sum of Squares	Mean Square	F Value	Pr > F
Model	4	8771.99462	2192.99866	6.59	0.0005
Error	35	11642.17938	332.63370		
Corrected Total	39	20414.17400			

R-Square	Coeff Var	Root MSE	Area Mean
0.429701	10.43169	18.23825	174.8350

Source	DF	Anova SS	Mean Square	F Value	Pr > F
**Day**	4	8771.994625	2192.998656	6.59	0.0005

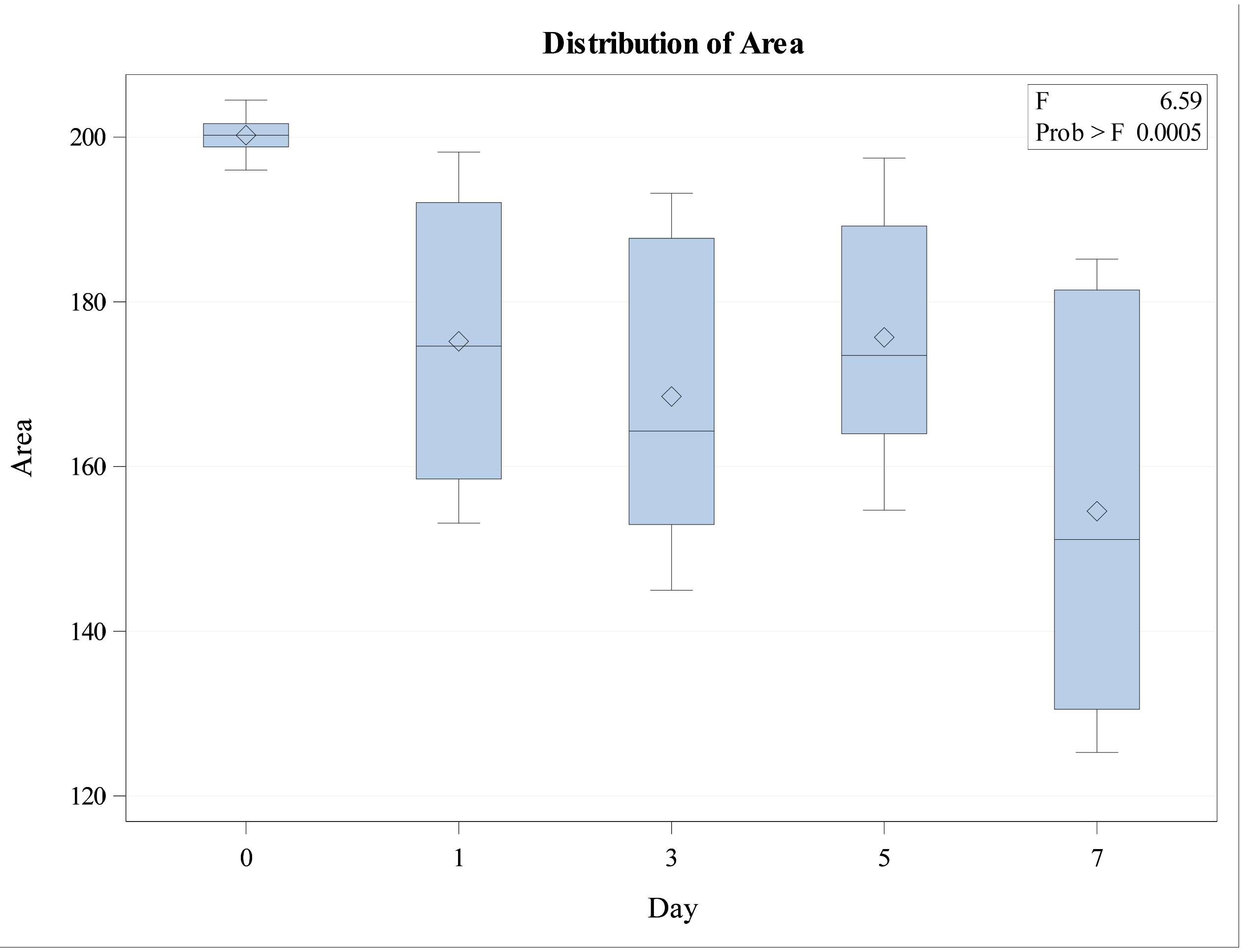

Biomechanical evaluation
Test between colagen concentration

Concentration=20

Class Level Information		
**Class**	**Levels**	**Values**
**Day**	5	0 1 3 5 7

**Number of Observations Read**	40
**Number of Observations Used**	40

Dependent Variable: Height

Concentration=20

Source	DF	Sum of Squares	Mean Square	F Value	Pr > F
**Model**	4	0.29971000	0.07492750	9.98	<.0001
**Error**	35	0.26285000	0.00751000		
**Corrected Total**	39	0.56256000			

R-Square	Coeff Var	Root MSE	Height Mean
0.532761	8.068925	0.086660	1.074000

Source	DF	Anova SS	Mean Square	F Value	Pr > F
**Day**	4	0.29971000	0.07492750	9.98	<.0001

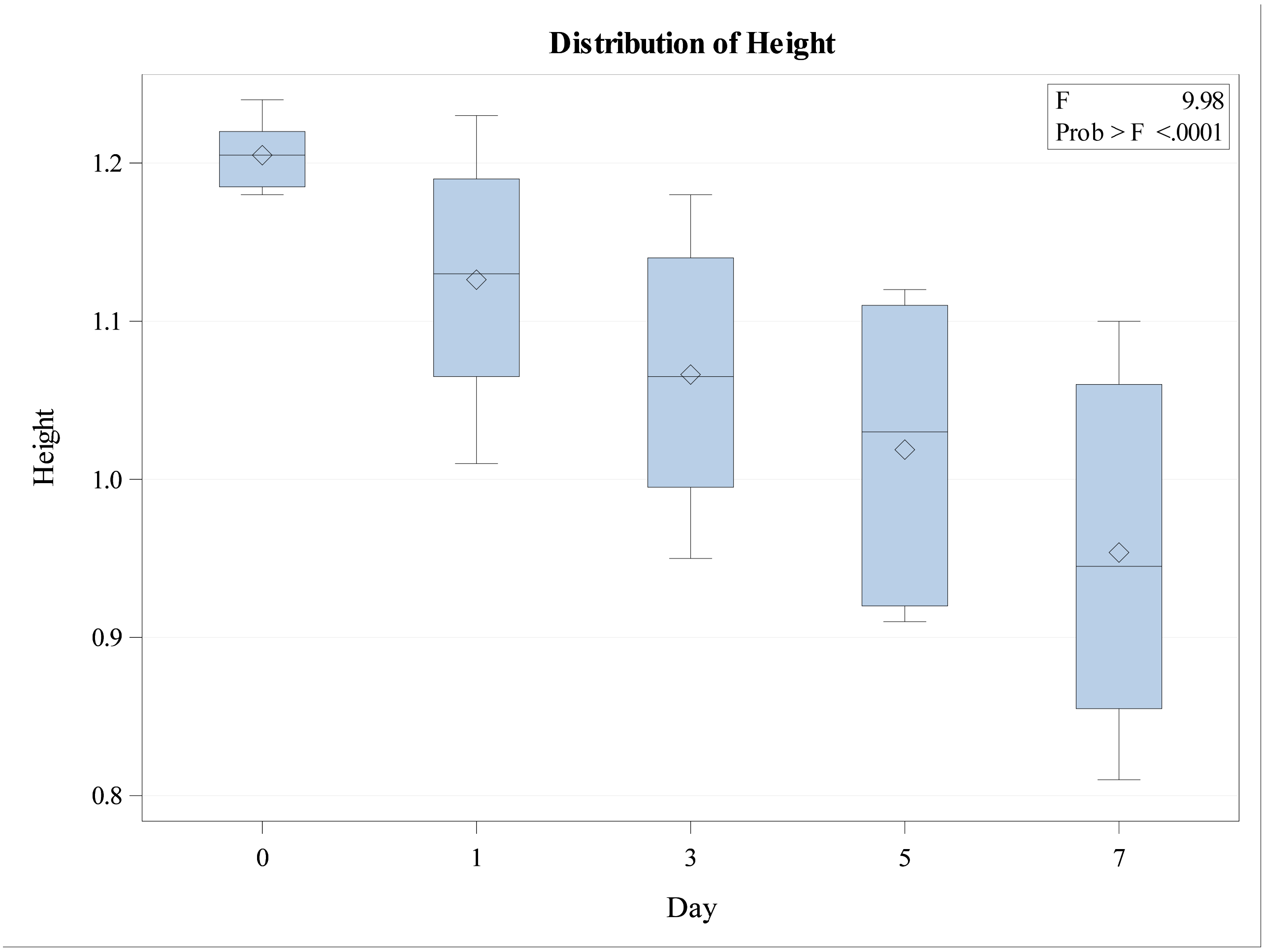

Concentration=30

Class Level Information		
**Class**	**Levels**	**Values**
**Day**	5	0 1 3 5 7

**Number of Observations Read**	40
**Number of Observations Used**	40

Dependent Variable: Height

Concentration=30

Source	DF	Sum of Squares	Mean Square	F Value	Pr > F
**Model**	4	0.12520000	0.03130000	20.50	<.0001
**Error**	35	0.05343750	0.00152679		
**Corrected Total**	39	0.17863750			

R-Square	Coeff Var	Root MSE	Height Mean
0.700861	3.431316	0.039074	1.138750

Source	DF	Anova SS	Mean Square	F Value	Pr > F
**Day**	4	0.12520000	0.03130000	20.50	<.0001

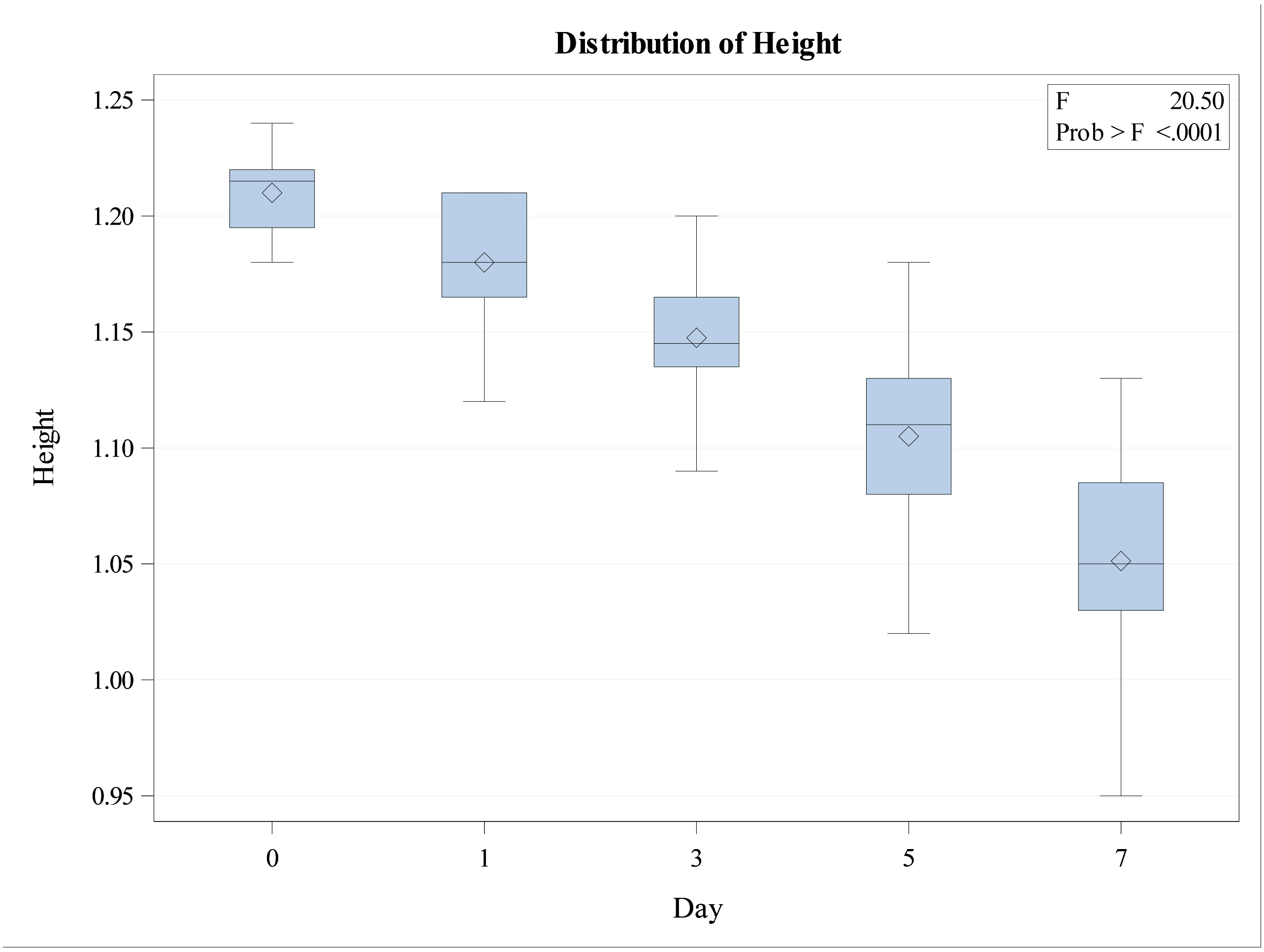

Biomechanical evaluation
Test between colagen concentration

Variable:
Area

Concentration=20

Cultivation	Method	N	Mean	Std Dev	Std Err	Minimum	Maximum
**D**		20	99.1900	51.6515	11.5496	54.8700	201.6
**S**		20	136.2	33.0296	7.3857	106.6	201.6
**Diff (1-2)**	**Pooled**		-36.9995	43.3522	13.7092		
**Diff (1-2)**	**Satterthwaite**		-36.9995		13.7092		

Cultivation	Method	Mean	95% CL Mean		Std Dev	95% CL Std Dev	
**D**		99.1900	75.0164	123.4	51.6515	39.2804	75.4407
**S**		136.2	120.7	151.6	33.0296	25.1187	48.2422

Cultivation	Method	Mean	95% CL Mean		Std Dev	95% CL Std Dev	
**Diff (1-2)**	**Pooled**	-36.9995	-64.7523	-9.2467	43.3522	35.4294	55.8714
**Diff (1-2)**	**Satterthwaite**	-36.9995	-64.9136	-9.0854			

Method	Variances	DF	t Value	Pr > |t|
**Pooled**	Equal	38	-2.70	0.0103
**Satterthwaite**	Unequal	32.313	-2.70	0.0110

Equality of Variances				
**Method**	**Num DF**	**Den DF**	**F Value**	**Pr > F**
**Folded F**	19	19	2.45	0.0583

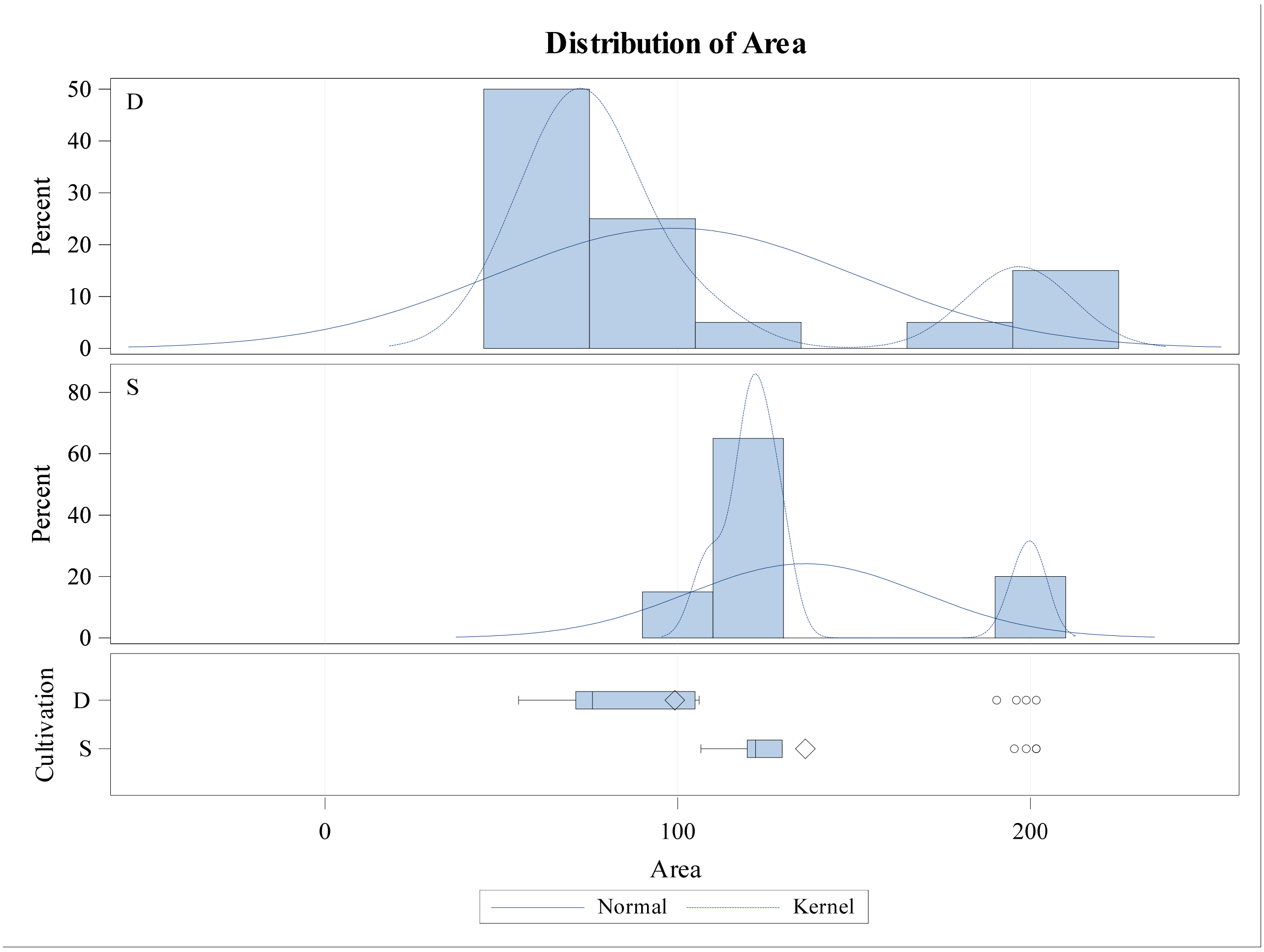

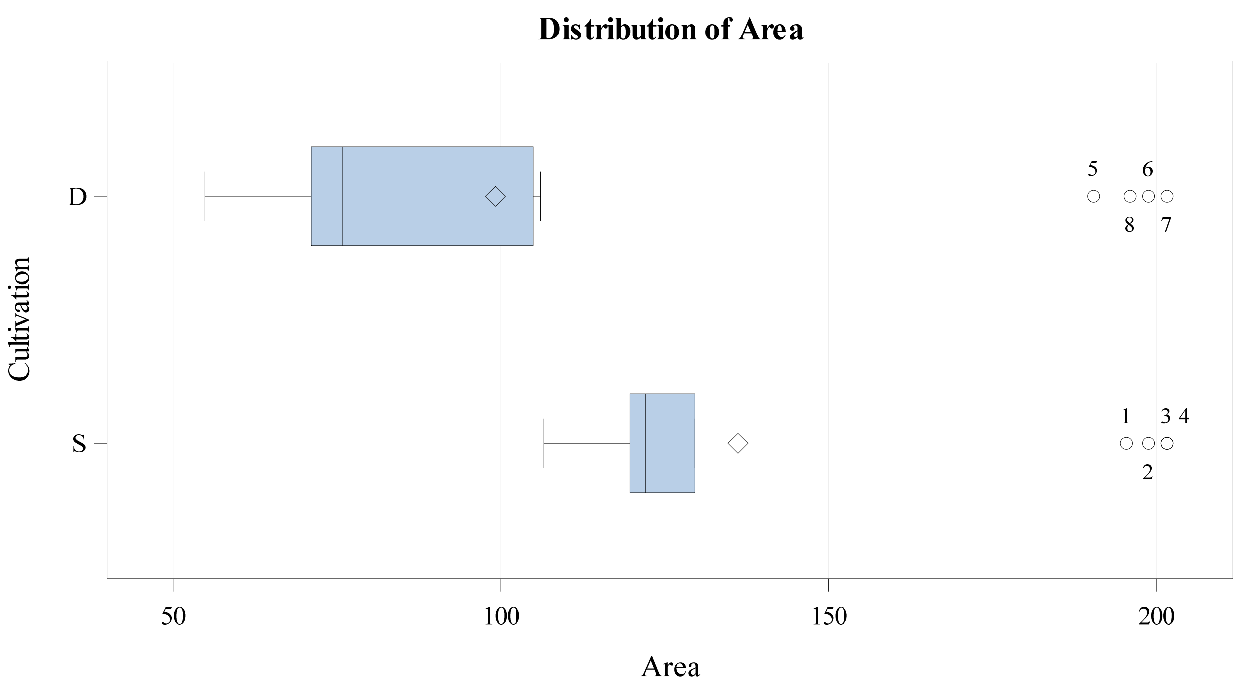

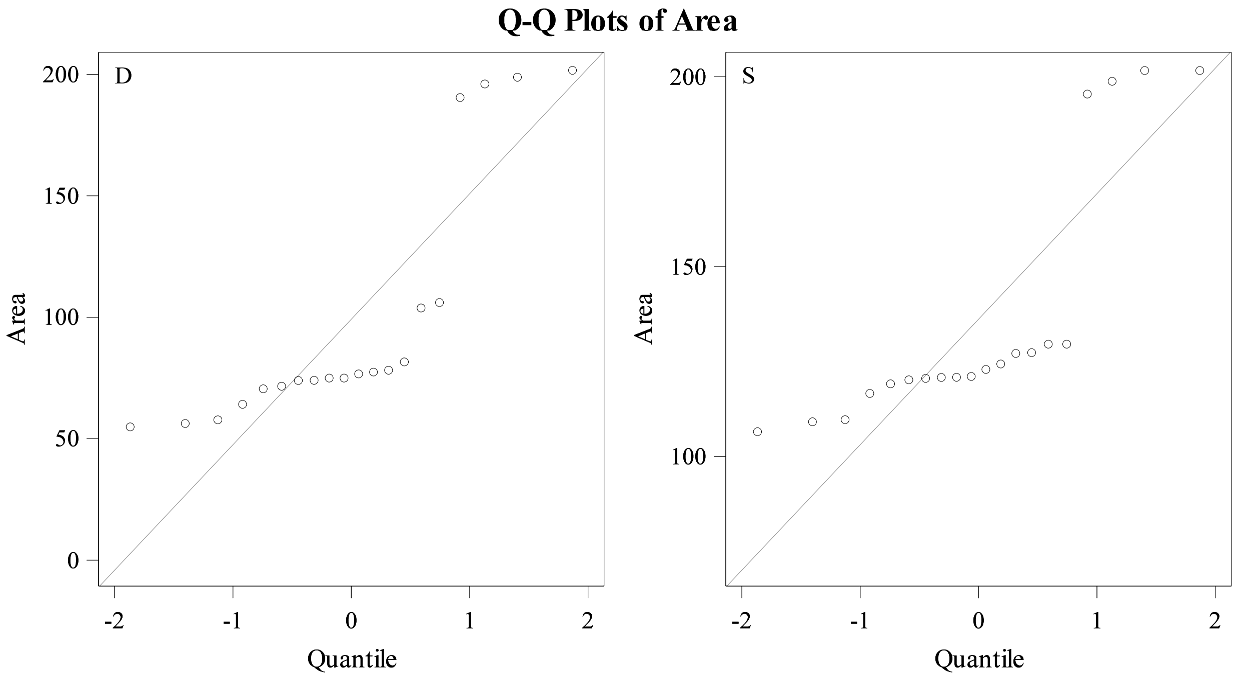

Variable:
Area

Concentration=30

Cultivation	Method	N	Mean	Std Dev	Std Err	Minimum	Maximum
**D**		20	160.4	23.2004	5.1878	125.3	201.6
**S**		20	189.3	9.8511	2.2028	168.4	204.5
**Diff (1-2)**	**Pooled**		-28.8850	17.8228	5.6361		

Cultivation	Method	N	Mean	Std Dev	Std Err	Minimum	Maximum
**Diff (1-2)**	**Satterthwaite**		-28.8850		5.6361		

Cultivation	Method	Mean	95% CL Mean		Std Dev	95% CL Std Dev	
**D**		160.4	149.5	171.3	23.2004	17.6437	33.8858
**S**		189.3	184.7	193.9	9.8511	7.4917	14.3882
**Diff (1-2)**	**Pooled**	-28.8850	-40.2946	-17.4754	17.8228	14.5656	22.9696
**Diff (1-2)**	**Satterthwaite**	-28.8850	-40.4781	-17.2919			

Method	Variances	DF	t Value	Pr > |t|
**Pooled**	Equal	38	-5.13	<.0001
**Satterthwaite**	Unequal	25.635	-5.13	<.0001

Equality of Variances				
**Method**	**Num DF**	**Den DF**	**F Value**	**Pr > F**
**Folded F**	19	19	5.55	0.0005

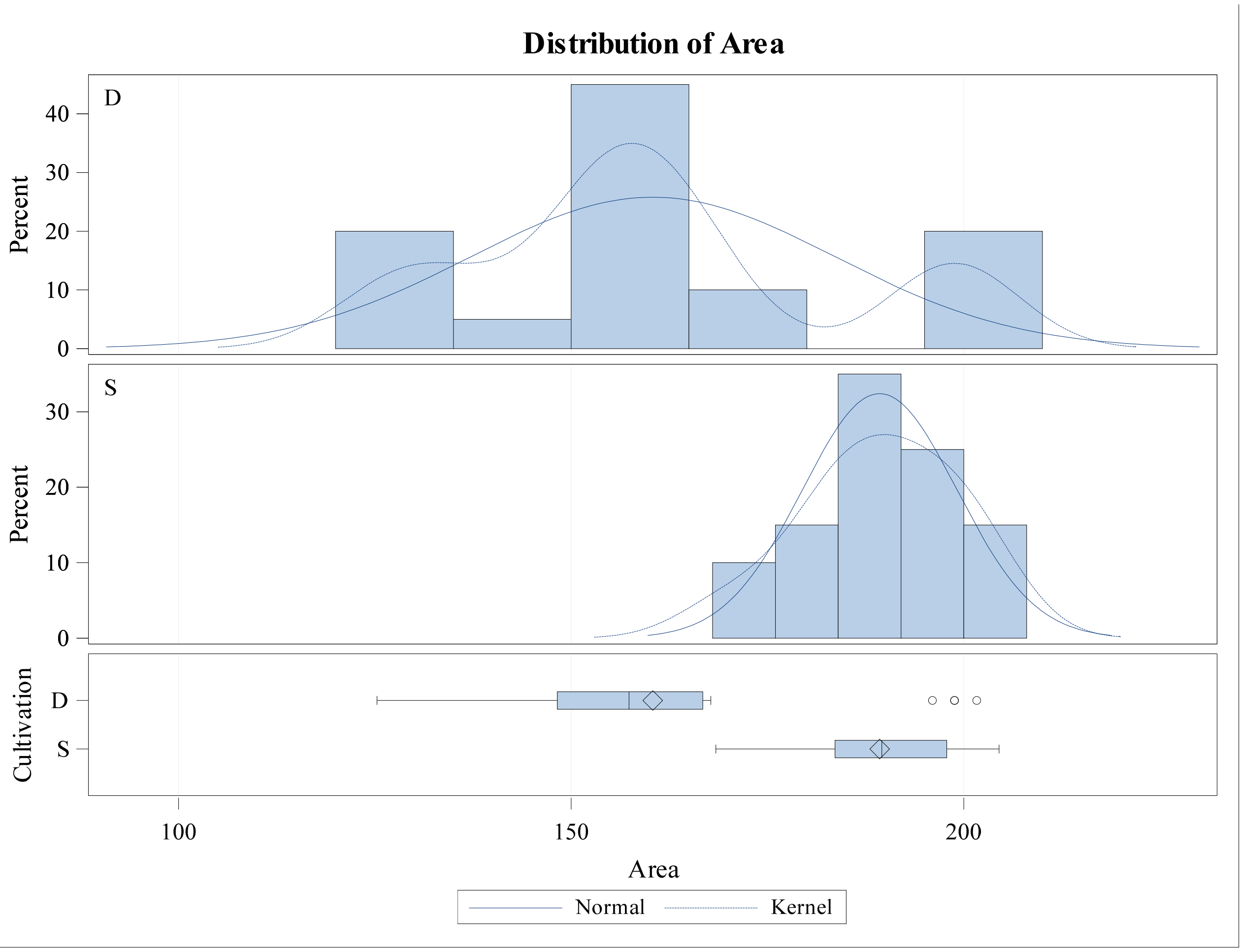

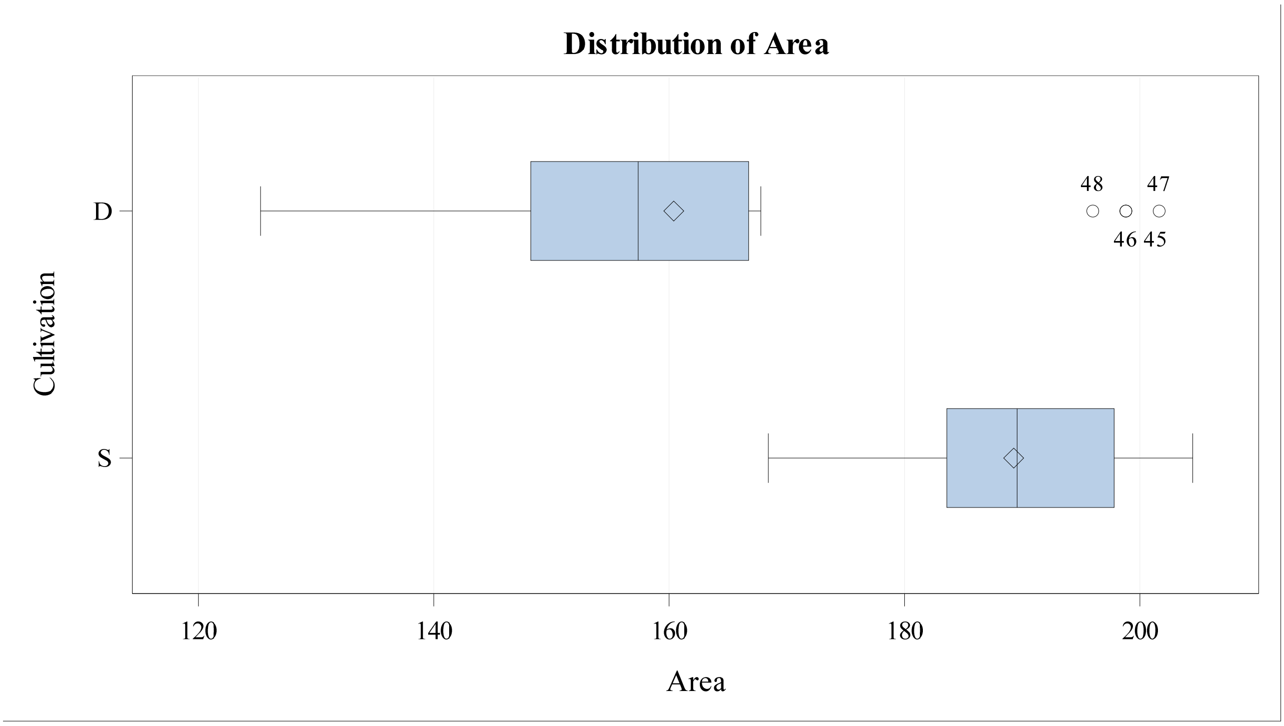

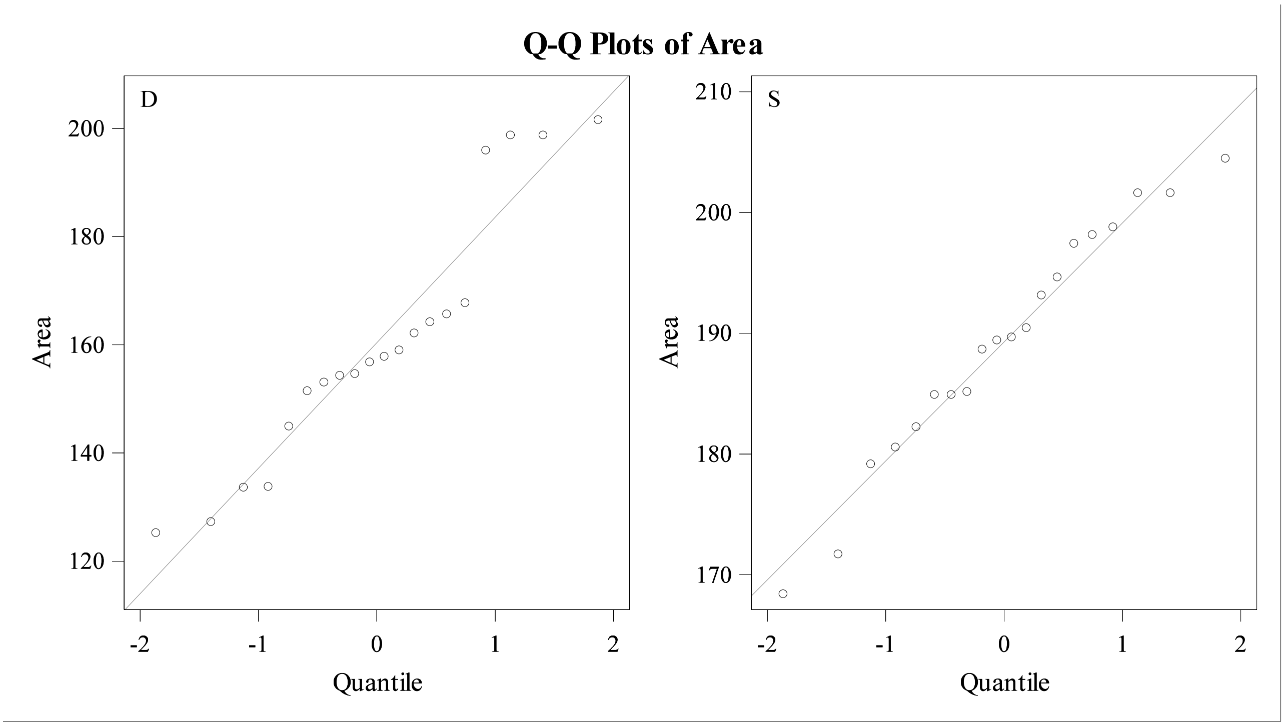

Biomechanical evaluation
Test between colagen concentration

Variable:
Height

Concentration=20

Cultivation	Method	N	Mean	Std Dev	Std Err	Minimum	Maximum
**D**		20	1.0085	0.1288	0.0288	0.8100	1.2400
**S**		20	1.1395	0.0632	0.0141	0.9800	1.2300
**Diff (1-2)**	**Pooled**		-0.1310	0.1014	0.0321		
**Diff (1-2)**	**Satterthwaite**		-0.1310		0.0321		

Cultivation	Method	Mean	95% CL Mean		Std Dev	95% CL Std Dev	
**D**		1.0085	0.9482	1.0688	0.1288	0.0979	0.1881
**S**		1.1395	1.1099	1.1691	0.0632	0.0481	0.0923
**Diff (1-2)**	**Pooled**	-0.1310	-0.1959	-0.0661	0.1014	0.0829	0.1307
**Diff (1-2)**	**Satterthwaite**	-0.1310	-0.1967	-0.0653			

Method	Variances	DF	t Value	Pr > |t|
**Pooled**	Equal	38	-4.08	0.0002
**Satterthwaite**	Unequal	27.652	-4.08	0.0003

Equality of Variances				
**Method**	**Num DF**	**Den DF**	**F Value**	**Pr > F**
**Folded F**	19	19	4.15	0.0032

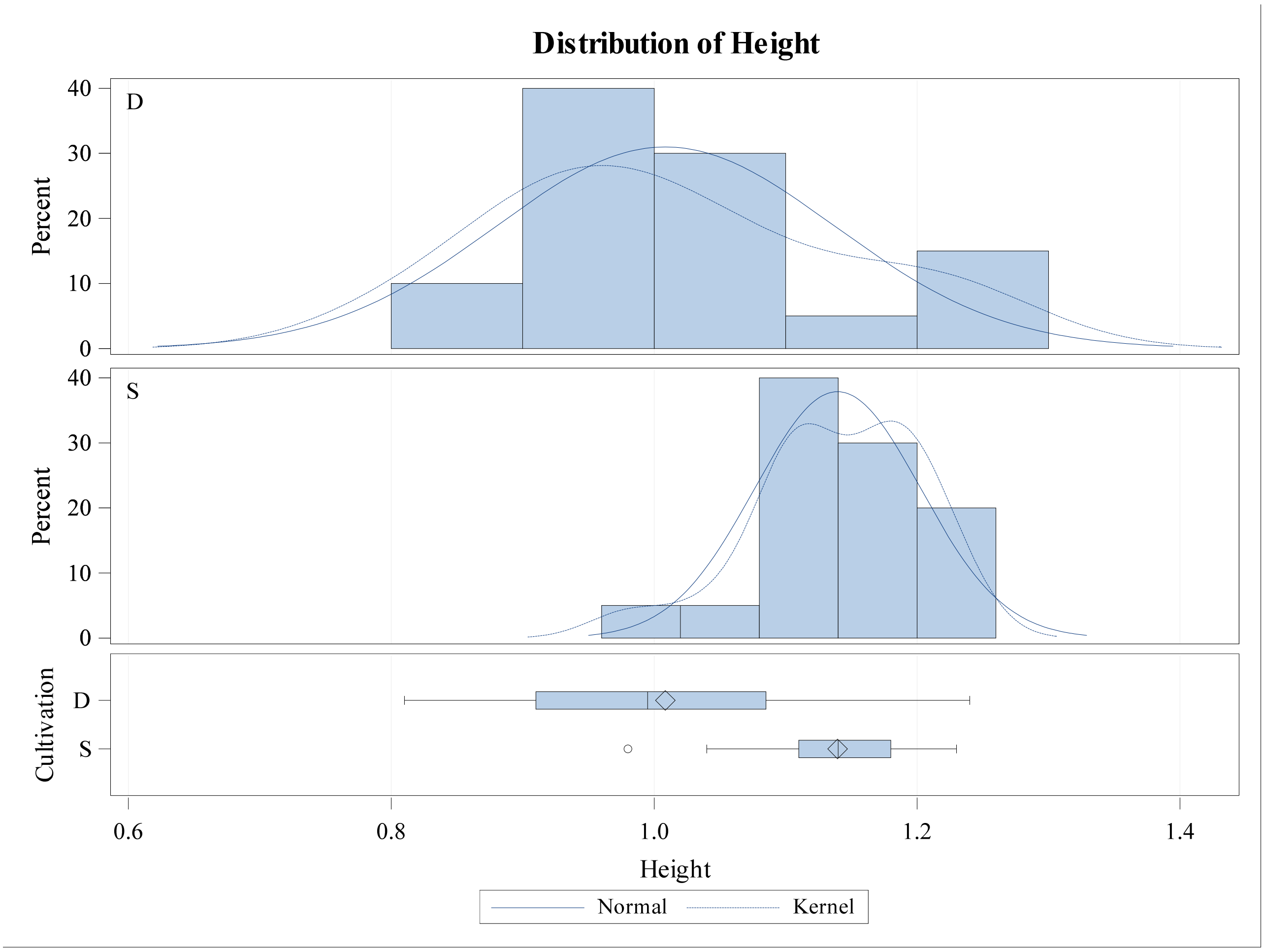

Distribution of Height
D
S
Percent
Cultivation
0
10
20
30
40
0.6
0.8
1.0
1.2
1.4
Height
Normal
Kernel

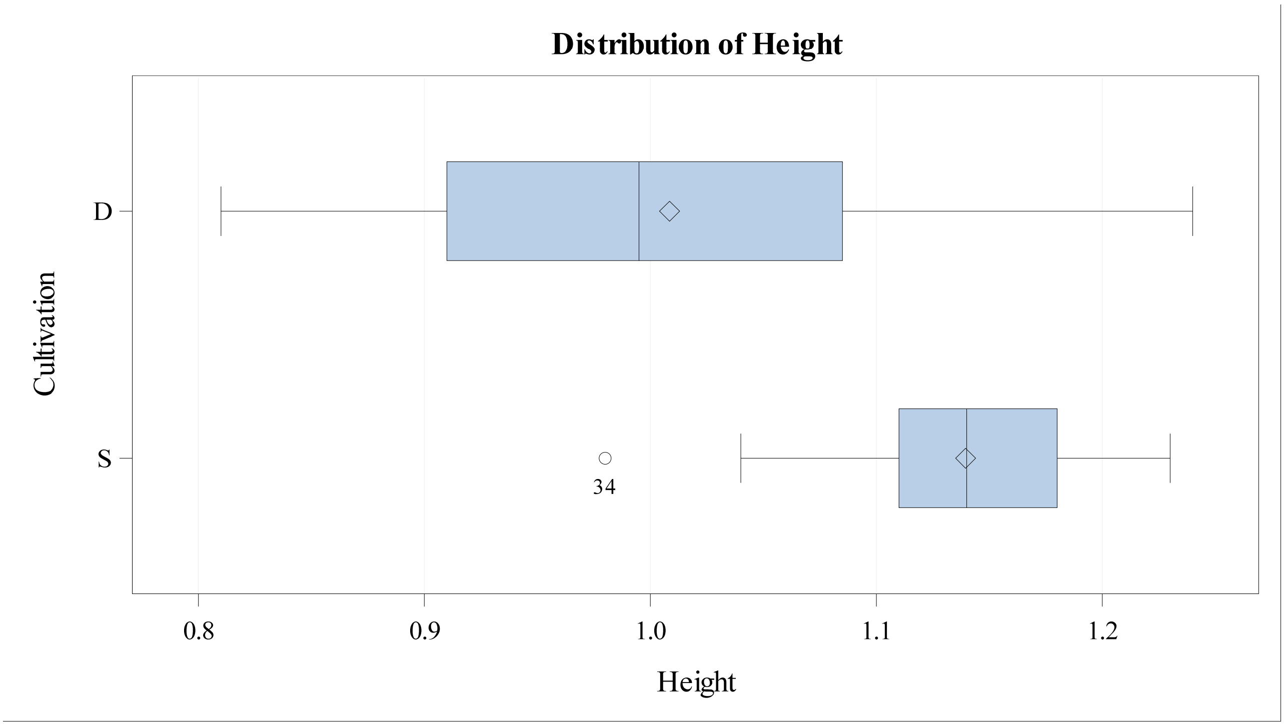

Distribution of Height
D
S
Cultivation
34
0.8
0.9
1.0
1.1
1.2
Height

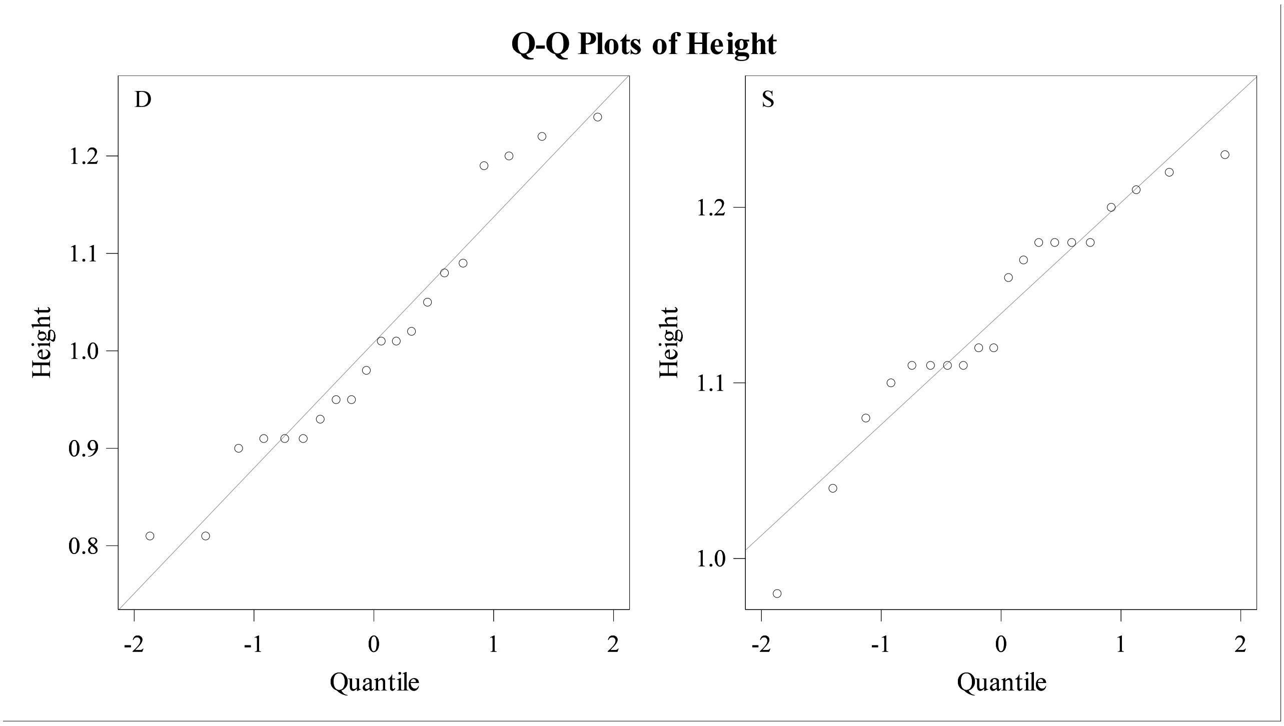

Variable: Height

Concentration=30

Cultivation	Method	N	Mean	Std Dev	Std Err	Minimum	Maximum
**D**		20	1.1250	0.0769	0.0172	0.9500	1.2400
**S**		20	1.1525	0.0555	0.0124	1.0500	1.2200
**Diff (1-2)**	**Pooled**		-0.0275	0.0671	0.0212		
**Diff (1-2)**	**Satterthwaite**		-0.0275		0.0212		

Cultivation	Method	Mean	95% CL Mean		Std Dev	95% CL Std Dev	
**D**		1.1250	1.0890	1.1610	0.0769	0.0585	0.1124
**S**		1.1525	1.1265	1.1785	0.0555	0.0422	0.0811
**Diff (1-2)**	**Pooled**	-0.0275	-0.0705	0.0155	0.0671	0.0548	0.0865
**Diff (1-2)**	**Satterthwaite**	-0.0275	-0.0706	0.0156			

Method	Variances	DF	t Value	Pr > |t|
**Pooled**	Equal	38	-1.30	0.2028
**Satterthwaite**	Unequal	34.566	-1.30	0.2035

Equality of Variances				
**Method**	**Num DF**	**Den DF**	**F Value**	**Pr > F**
**Folded F**	19	19	1.92	0.1640

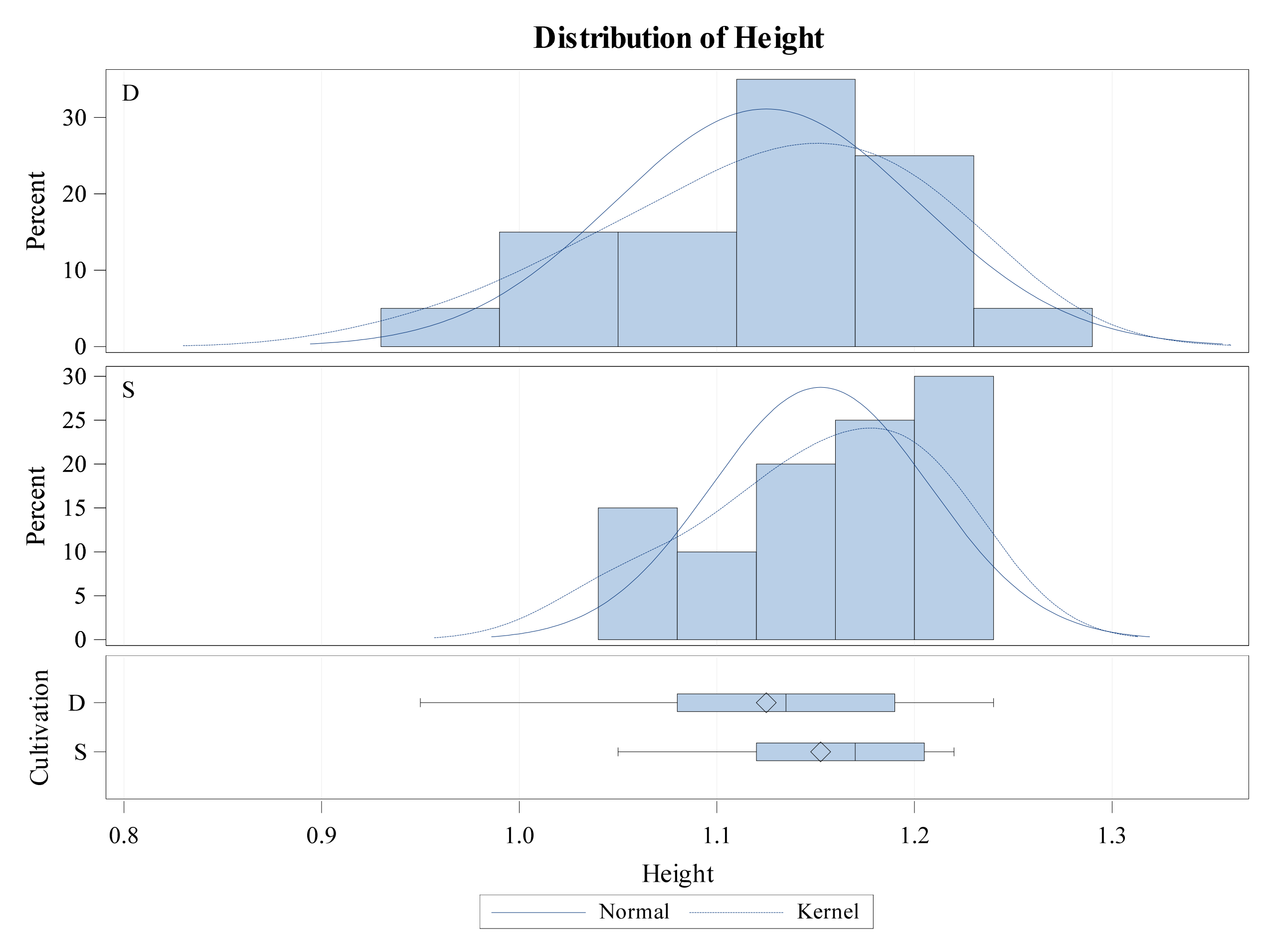

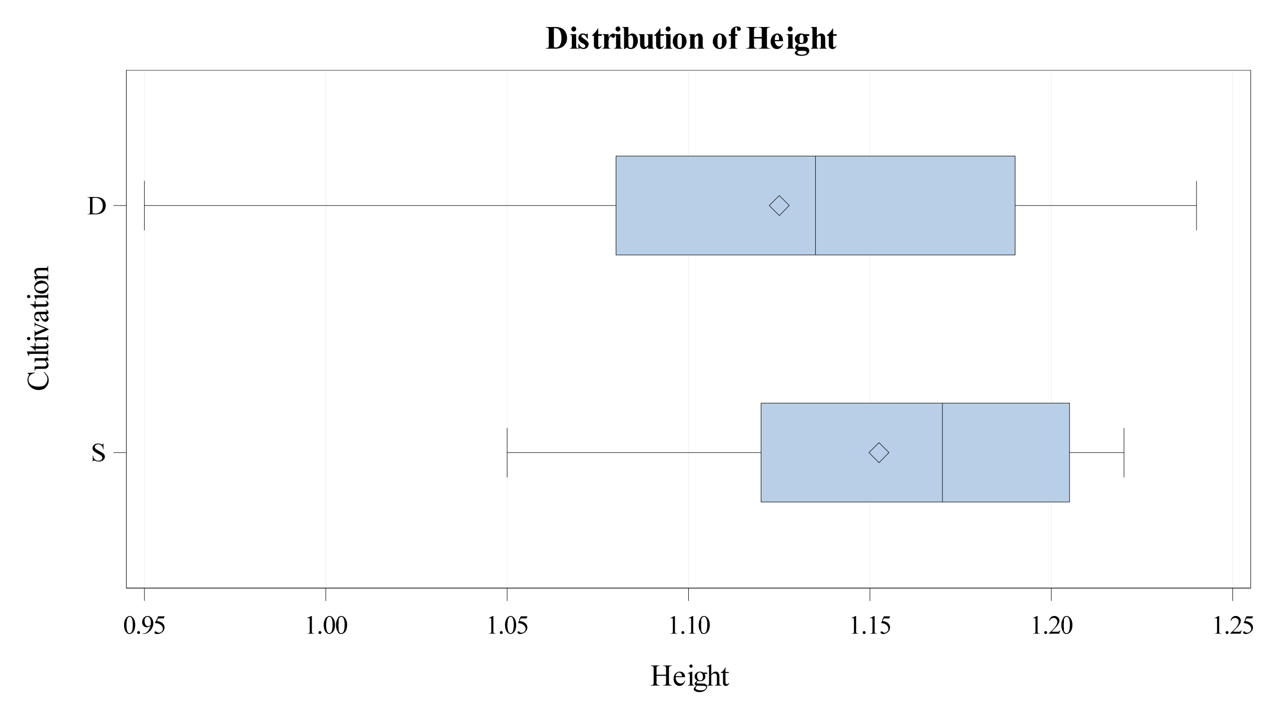

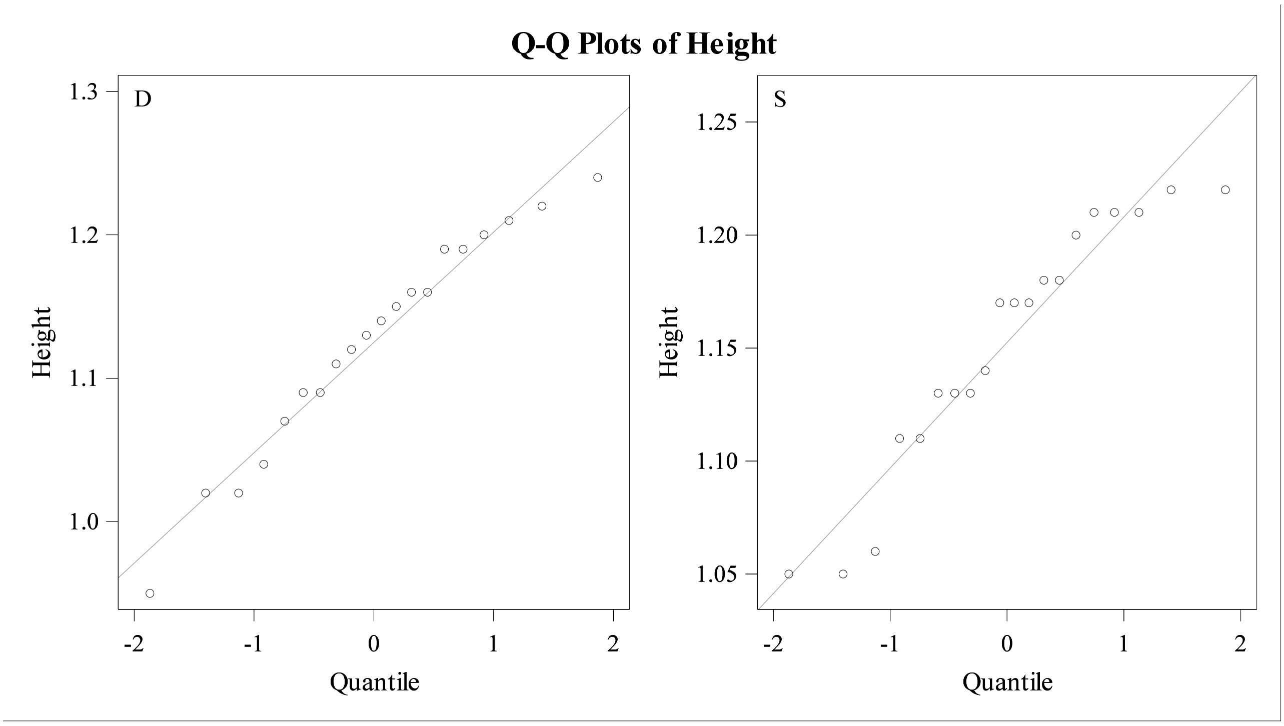

Biomechanical evaluation
Test between colagen concentration

Concentration=20

Class Level Information		
**Class**	**Levels**	**Values**
**Day**	5	0 1 3 5 7
**Cultivation**	2	D S

**Number of Observations Read**	40
**Number of Observations Used**	40

Dependent Variable: Area

Concentration=20

Source	DF	Sum of Squares	Mean Square	F Value	Pr > F
**Model**	5	79182.24154	15836.44831	90.87	<.0001
**Error**	34	5925.17616	174.26989		
**Corrected Total**	39	85107.41770			

R-Square	Coeff Var	Root MSE	Area Mean
0.930380	11.21689	13.20113	117.6898

Source	DF	Anova SS	Mean Square	F Value	Pr > F
Day	4	65492.61153	16373.15288	93.95	<.0001
Cultivation	1	13689.63000	13689.63000	78.55	<.0001

Concentration=30

Class Level Information		
Class	Levels	Values
Day	5	0 1 3 5 7
Cultivation	2	D S

Number of Observations Read	40
Number of Observations Used	40

Dependent Variable: Area

Concentration=30

Source	DF	Sum of Squares	Mean Square	F Value	Pr > F
Model	5	17115.42687	3423.08537	35.28	<.0001
Error	34	3298.74713	97.02197		
Corrected Total	39	20414.17400			

R-Square	Coeff Var	Root MSE	Area Mean
0.838409	5.633868	9.849973	174.8350

Source	DF	Anova SS	Mean Square	F Value	Pr > F
Day	4	8771.994625	2192.998656	22.60	<.0001
Cultivation	1	8343.432250	8343.432250	86.00	<.0001

Biomechanical evaluation
Test between colagen concentration

Model: MODEL1
Dependent Variable: Area

Concentration=20

**Number of Observations Read**	40
**Number of Observations Used**	40

Stepwise Selection: Step 1

Variable Day Entered: R-Square = 0.3876 and C(p) = 14.1799

Analysis of Variance					
**Source**	**DF**	**Sum of Squares**	**Mean Square**	**F Value**	**Pr > F**
**Model**	1	32987	32987	24.05	<.0001
**Error**	38	52121	1371.59832		
**Corrected Total**	39	85107			

Variable	Parameter Estimate	Standard Error	Type II SS	F Value	Pr > F
**Intercept**	153.56851	9.37101	368348	268.55	<.0001
**Day**	-11.21211	2.28629	32987	24.05	<.0001

Bounds on condition number: 1, 1

Stepwise Selection: Step 2

Variable cultivation_new Entered: R-Square = 0.5484 and C(p) = 3.0000

Analysis of Variance					
**Source**	**DF**	**Sum of Squares**	**Mean Square**	**F Value**	**Pr > F**
**Model**	2	46676	23338	22.47	<.0001
**Error**	37	38431	1038.67854		
**Corrected Total**	39	85107			

Variable	Parameter Estimate	Standard Error	Type II SS	F Value	Pr > F
**Intercept**	209.06776	17.32638	151231	145.60	<.0001
**Day**	-11.21211	1.98957	32987	31.76	<.0001
**cultivation_new**	-36.99950	10.19156	13690	13.18	0.0009

Bounds on condition number: 1, 4

All variables left in the model are significant at the 0.1500 level.

All variables have been entered into the model.

Summary of Stepwise Selection									
**Step**	**Variable Entered**	**Variable Removed**	**Label**	**Number Vars In**	**Partial R-Square**	**Model R-Square**	**C(p)**	**F Value**	**Pr > F**
**1**	Day			1	0.3876	0.3876	14.1799	24.05	<.0001
**2**	cultivation_new		Cultivation type	2	0.1609	0.5484	3.0000	13.18	0.0009

Model: MODEL1
Dependent Variable: Area

Concentration=20

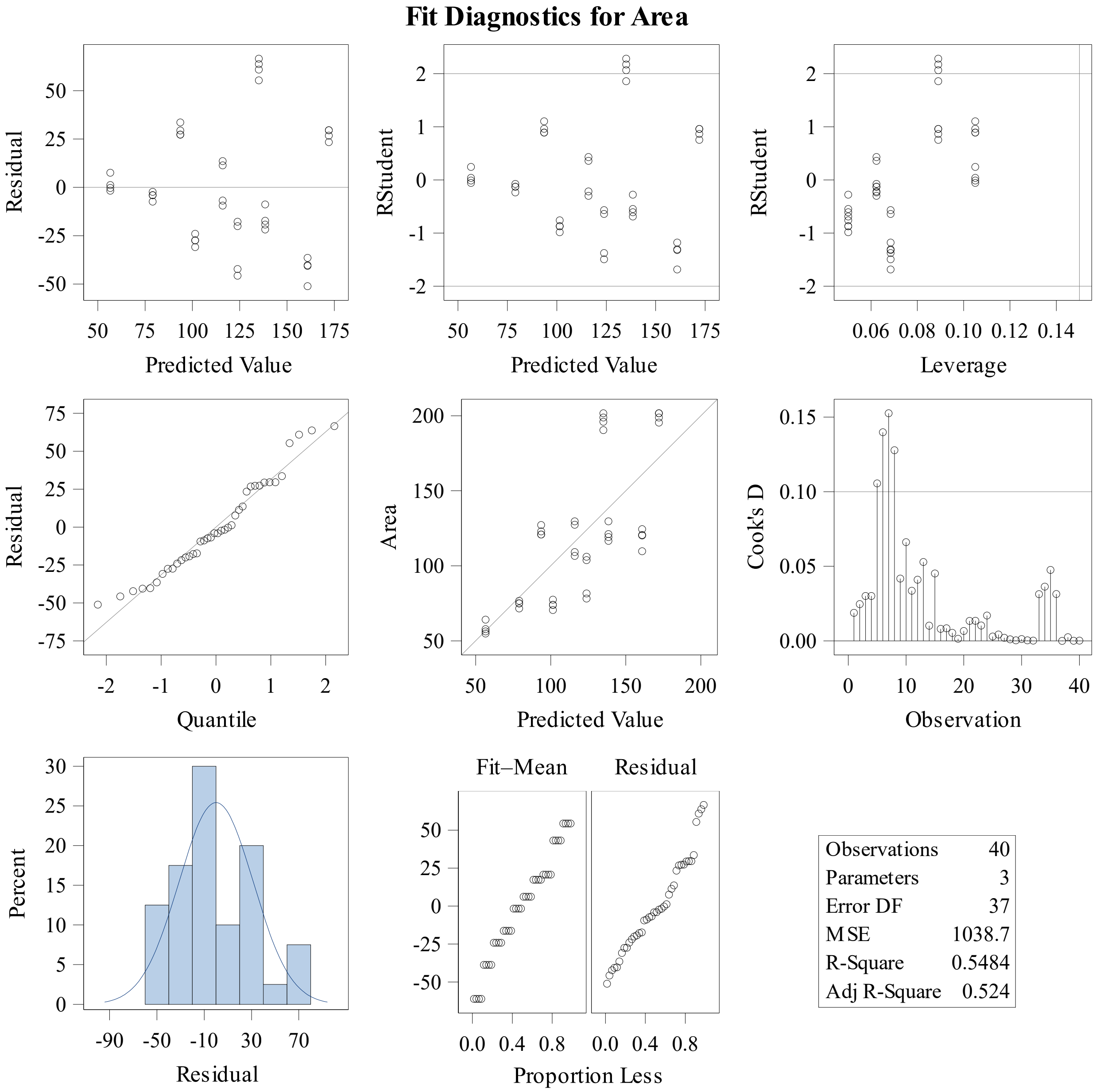

Observations	40
Parameters	3
Error DF	37
MSE	1038.7
R-Square	0.5484
Adj R-Square	0.524

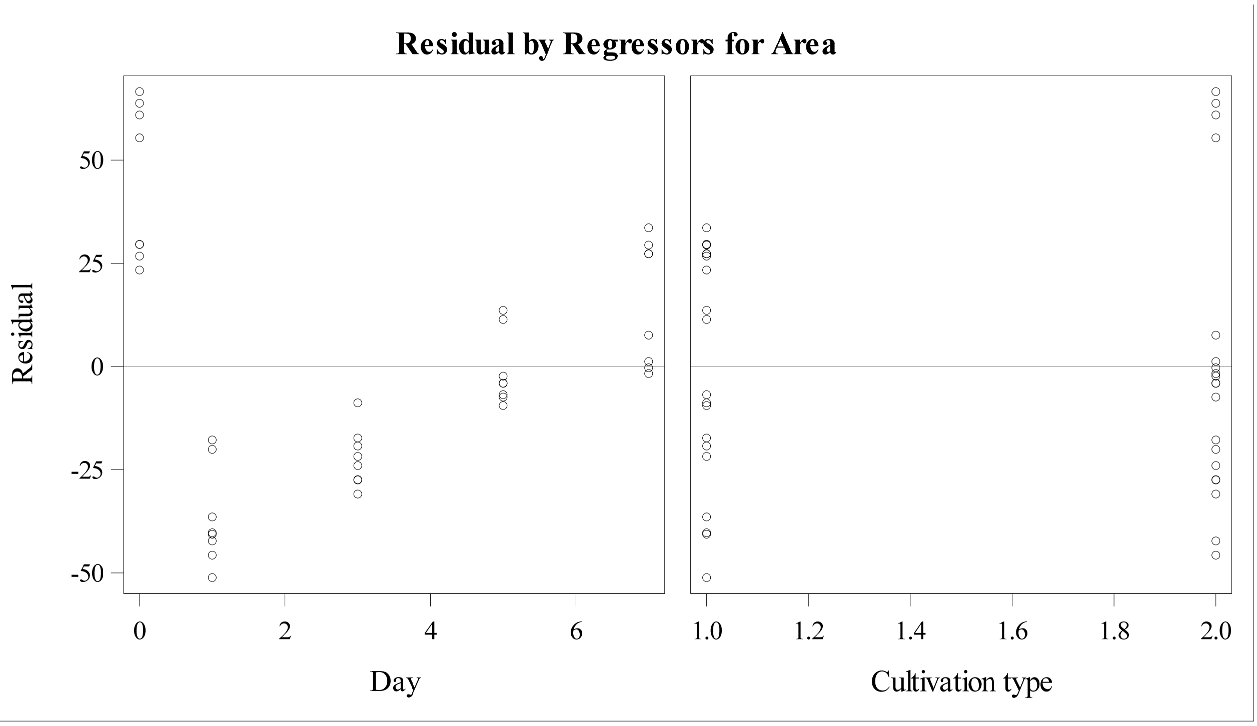

Model: MODEL1
Dependent Variable: Area

Concentration=30

**Number of Observations Read**	40
**Number of Observations Used**	40

Stepwise Selection: Step 1

Variable cultivation_new Entered: R-Square = 0.4087 and C(p) = 37.0438

Analysis of Variance					
**Source**	**DF**	**Sum of Squares**	**Mean Square**	**F Value**	**Pr > F**
**Model**	1	8343.43225	8343.43225	26.27	<.0001
**Error**	38	12071	317.65110		
**Corrected Total**	39	20414			

**Variable**	**Parameter Estimate**	**Standard Error**	**Type II SS**	**F Value**	**Pr > F**
**Intercept**	218.16250	8.91138	190380	599.34	<.0001
**cultivation_new**	-28.88500	5.63605	8343.43225	26.27	<.0001

Bounds on condition number: 1, 1

Stepwise Selection: Step 2

Variable Day Entered: R-Square = 0.7005 and C(p) = 3.0000

Analysis of Variance					
**Source**	**DF**	**Sum of Squares**	**Mean Square**	**F Value**	**Pr > F**
**Model**	2	14300	7149.89835	43.27	<.0001
**Error**	37	6114.37730	165.25344		
**Corrected Total**	39	20414			

Variable	Parameter Estimate	Standard Error	Type II SS	F Value	Pr > F
**Intercept**	233.40860	6.91103	188495	1140.64	<.0001
**Day**	-4.76441	0.79358	5956.36445	36.04	<.0001
**cultivation_new**	-28.88500	4.06514	8343.43225	50.49	<.0001

Bounds on condition number: 1, 4

All variables left in the model are significant at the 0.1500 level.

All variables have been entered into the model.

Summary of Stepwise Selection									
**Step**	**Variable Entered**	**Variable Removed**	**Label**	**Number Vars In**	**Partial R-Square**	**Model R-Square**	**C(p)**	**F Value**	**Pr > F**
**1**	cultivation_new		Cultivation type	1	0.4087	0.4087	37.0438	26.27	<.0001
**2**	Day			2	0.2918	0.7005	3.0000	36.04	<.0001

Model: MODEL1
Dependent Variable: Area

Concentration=30

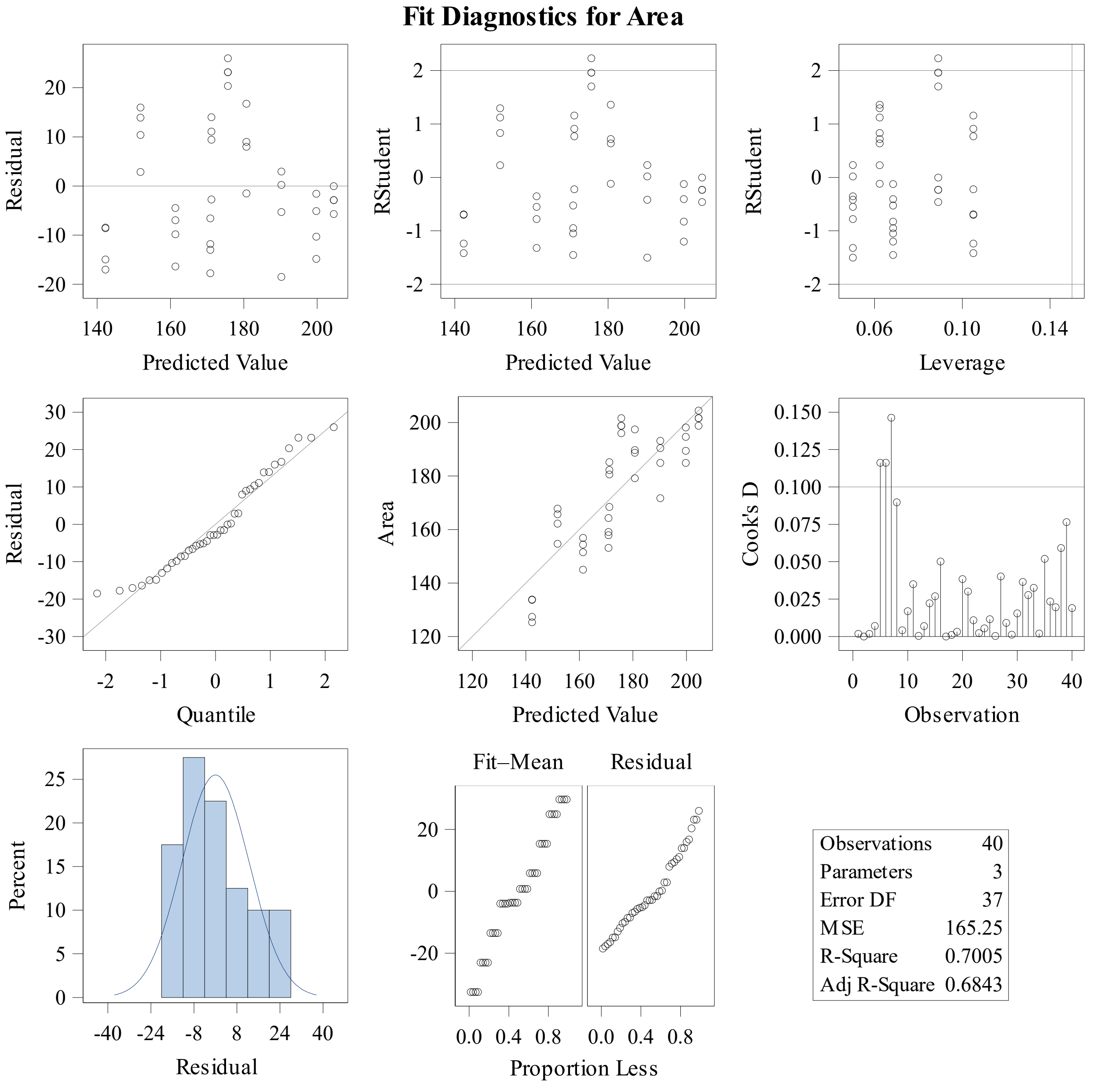

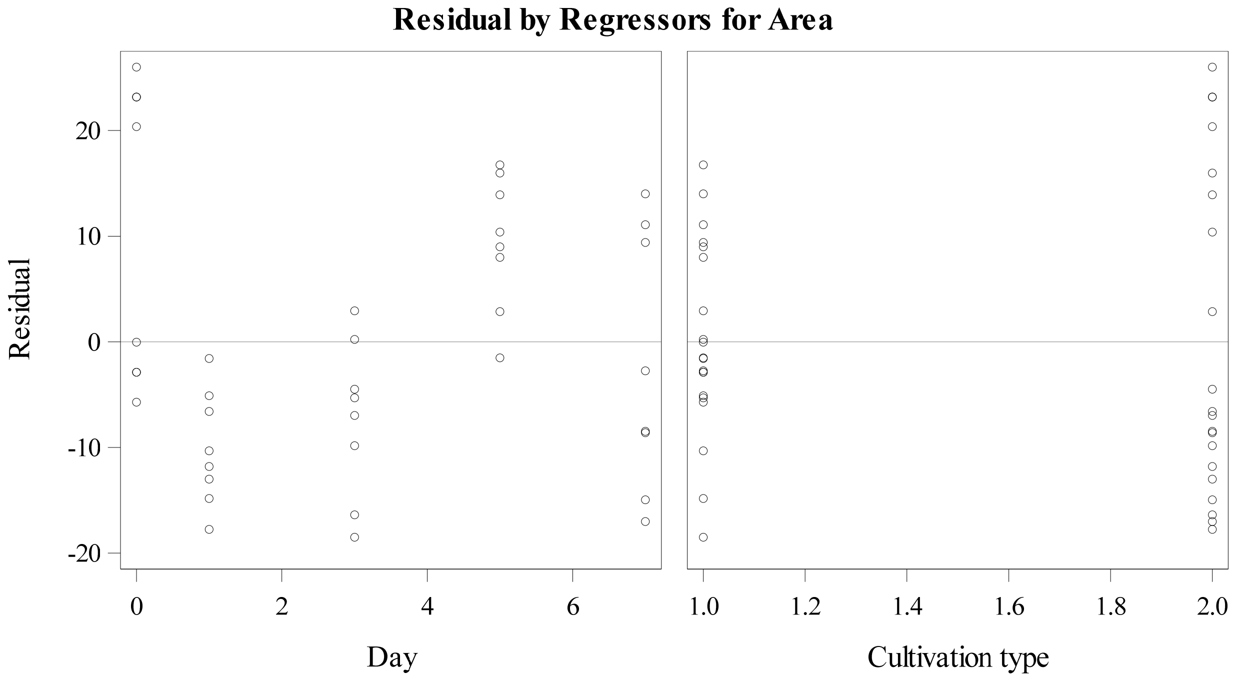

Biomechanical evaluation
Test of colagen concentration

Variable:
Area

Concentration	Method	N	Mean	Std Dev	Std Err	Minimum	Maximum
**20**		64	92.8005	74.4459	9.3057	9.8200	204.5
**30**		64	121.3	61.9355	7.7419	34.0300	208.8
**Diff (1-2)**	**Pooled**		-28.4780	68.4770	12.1051		
**Diff (1-2)**	**Satterthwaite**		-28.4780		12.1051		

Concentration	Method	Mean	95% CL Mean		Std Dev	95% CL Std Dev	
**20**		92.8005	74.2044	111.4	74.4459	63.4128	90.1629
**30**		121.3	105.8	136.7	61.9355	52.7565	75.0114
**Diff (1-2)**	**Pooled**	-28.4780	-52.4337	-4.5223	68.4770	60.9653	78.1166
**Diff (1-2)**	**Satterthwaite**	-28.4780	-52.4414	-4.5146			

Method	Variances	DF	t Value	Pr > |t|
**Pooled**	Equal	126	-2.35	0.0202
**Satterthwaite**	Unequal	121.96	-2.35	0.0202

Equality of Variances				
**Method**	**Num DF**	**Den DF**	**F Value**	**Pr > F**
**Folded F**	63	63	1.44	0.1469

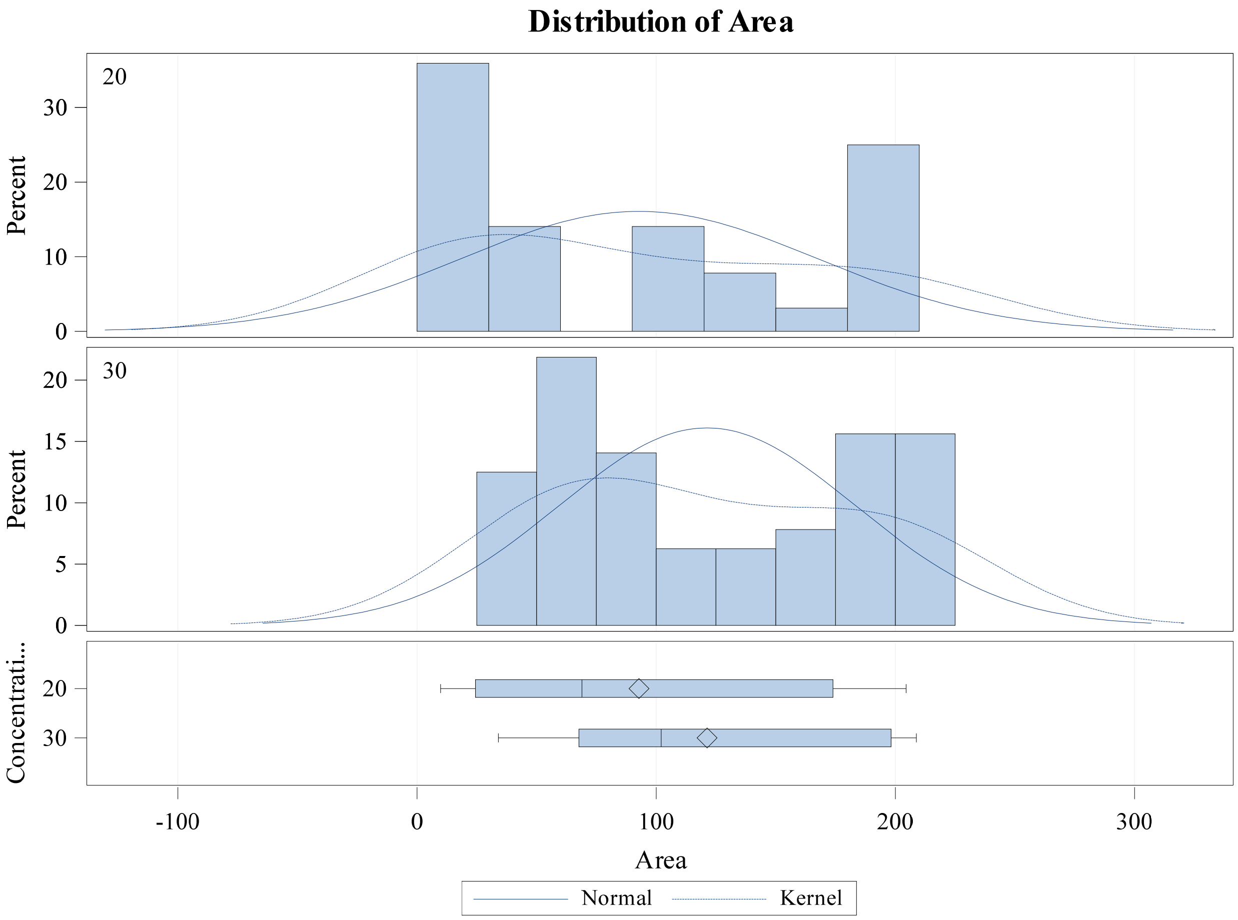

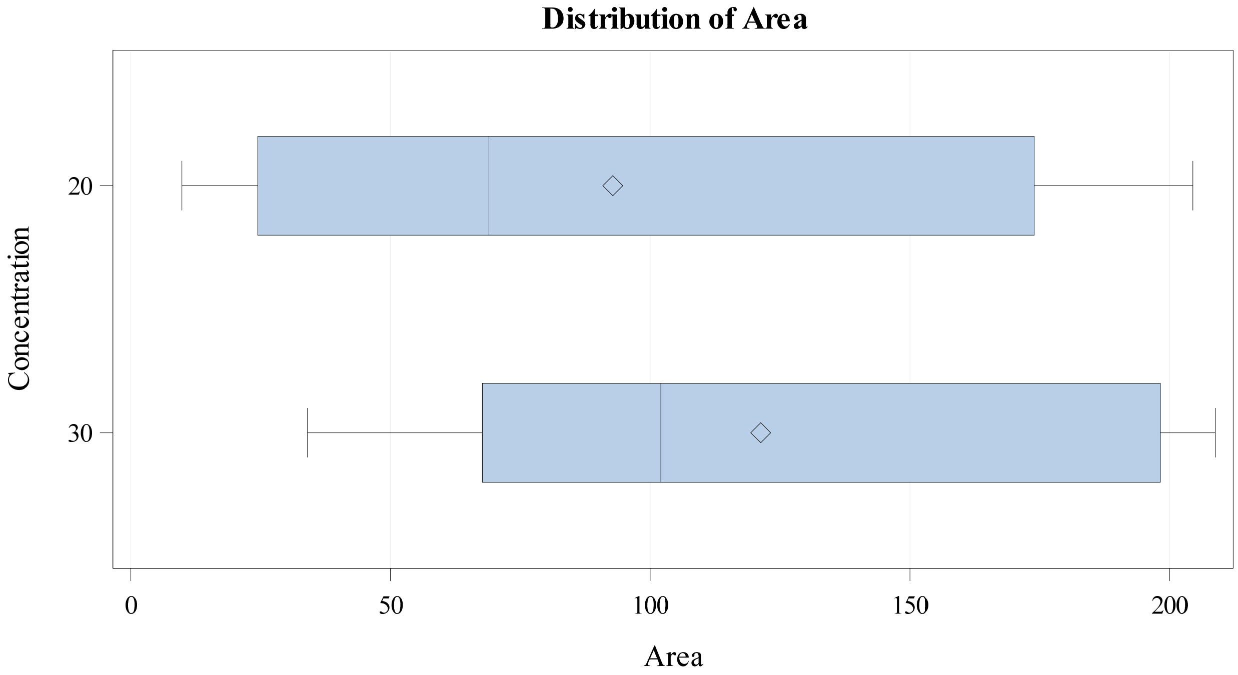

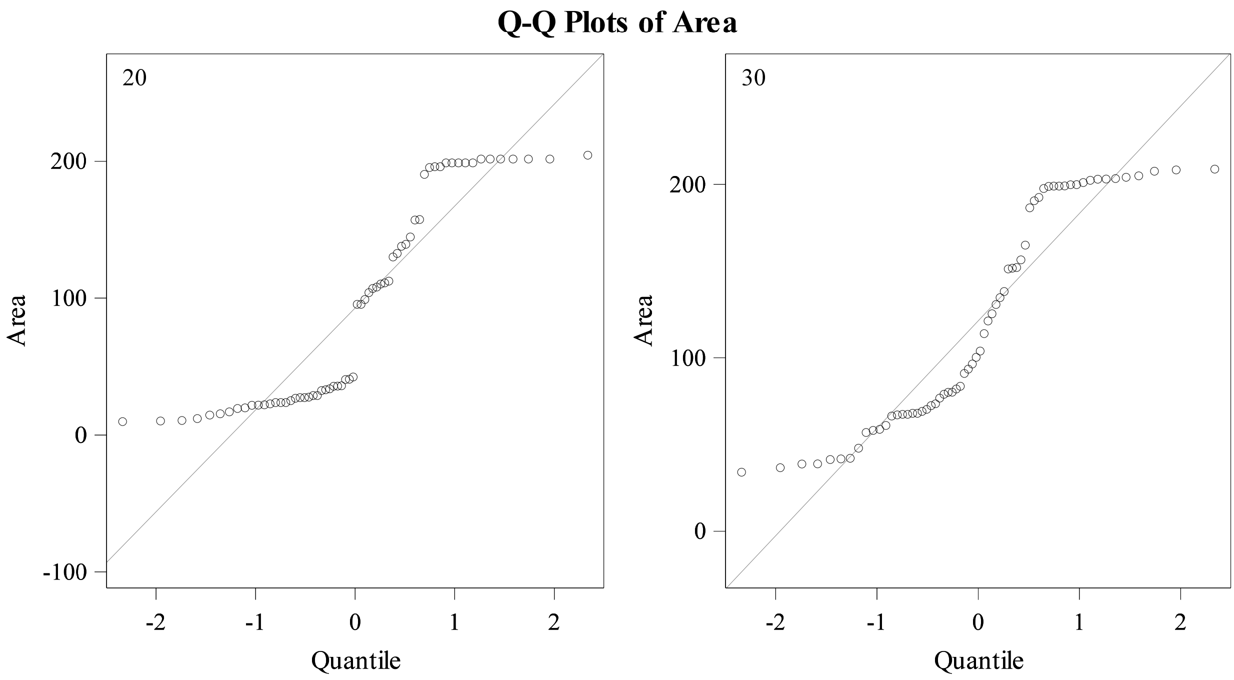

Biomechanical evaluation
Test of print height

Variable:
Area

Print_height	Method	N	Mean	Std Dev	Std Err	Minimum	Maximum
**0.8**		64	104.3	71.6215	8.9527	9.8200	207.6
**1.2**		64	109.8	68.1581	8.5198	16.9100	208.8
**Diff (1-2)**	**Pooled**		-5.4277	69.9113	12.3587		
**Diff (1-2)**	**Satterthwaite**		-5.4277		12.3587		

Print_height	Method	Mean	95% CL Mean		Std Dev	95% CL Std Dev	
**0.8**		104.3	86.4351	122.2	71.6215	61.0071	86.7423
**1.2**		109.8	92.7279	126.8	68.1581	58.0569	82.5476
**Diff (1-2)**	**Pooled**	-5.4277	-29.8851	19.0298	69.9113	62.2422	79.7527
**Diff (1-2)**	**Satterthwaite**	-5.4277	-29.8857	19.0304			

Method	Variances	DF	t Value	Pr > |t|
**Pooled**	Equal	126	-0.44	0.6613
**Satterthwaite**	Unequal	125.69	-0.44	0.6613

Equality of Variances				
**Method**	**Num DF**	**Den DF**	**F Value**	**Pr > F**
**Folded F**	63	63	1.10	0.6952

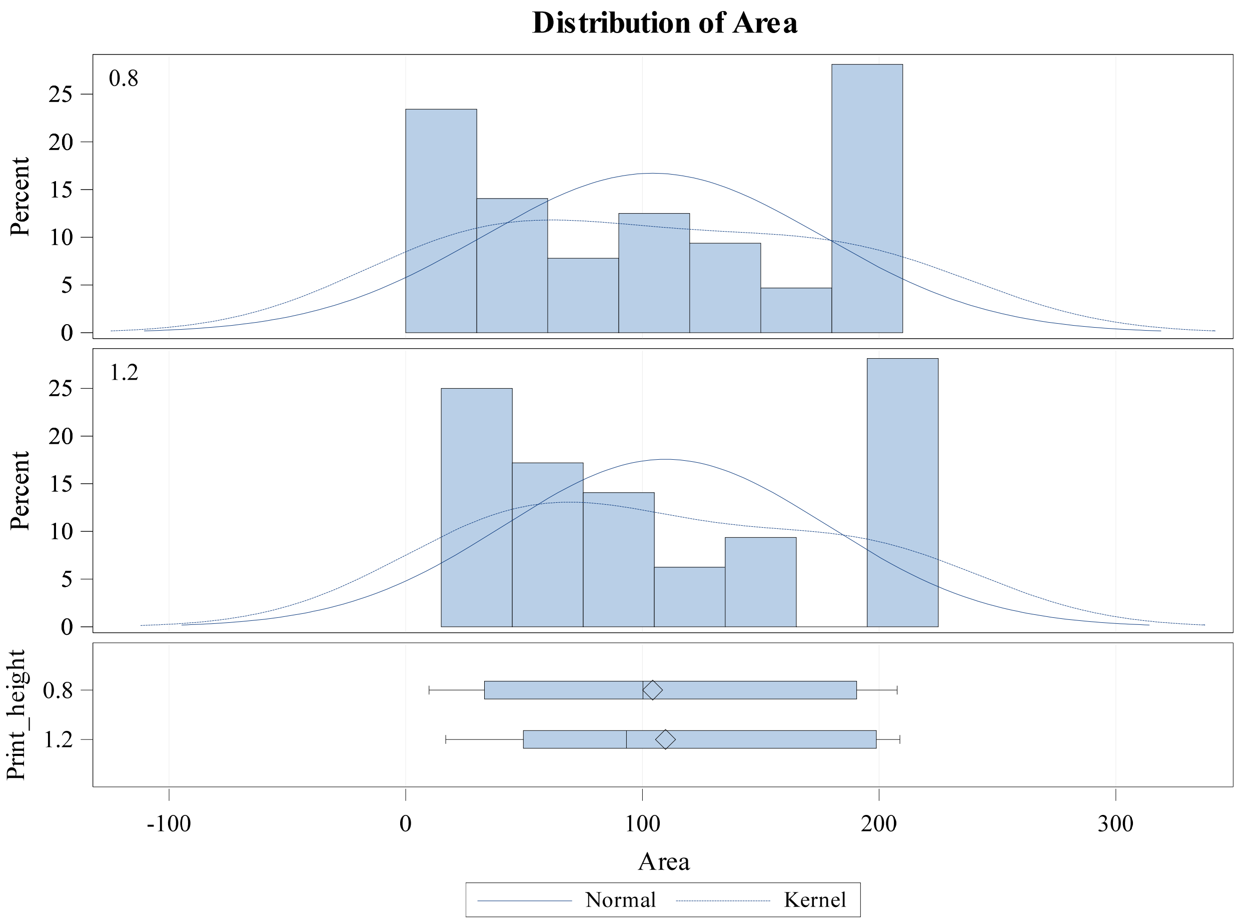

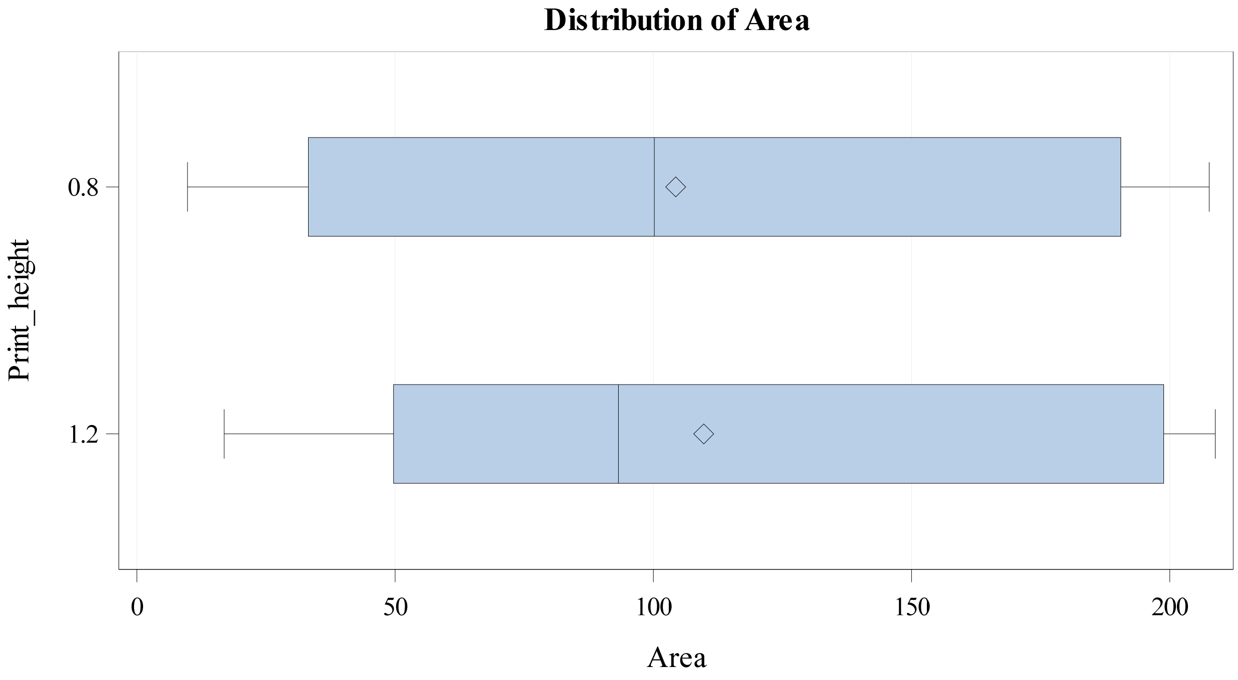

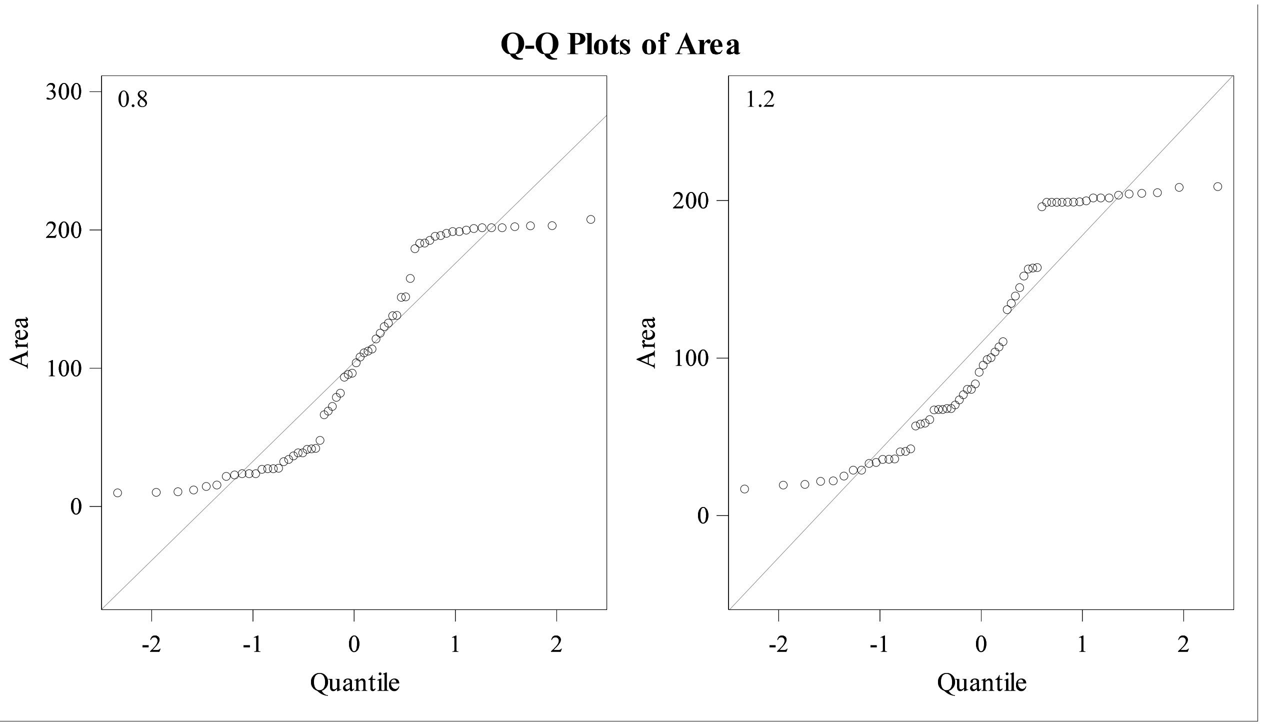

Biomechanical evaluation
Test of print height by colagen concentration

Variable:
Area

Concentration=20

Print_height	Method	N	Mean	Std Dev	Std Err	Minimum	Maximum
**0.8**		32	88.9450	75.8813	13.4141	9.8200	201.6
**1.2**		32	96.6559	73.9898	13.0797	16.9100	204.5
**Diff (1-2)**	**Pooled**		-7.7109	74.9415	18.7354		
**Diff (1-2)**	**Satterthwaite**		-7.7109		18.7354		

Print_height	Method	Mean	95% CL Mean		Std Dev	95% CL Std Dev	
**0.8**		88.9450	61.5869	116.3	75.8813	60.8343	100.9
**1.2**		96.6559	69.9798	123.3	73.9898	59.3178	98.3679
**Diff (1-2)**	**Pooled**	-7.7109	-45.1624	29.7406	74.9415	63.7595	90.9166
**Diff (1-2)**	**Satterthwaite**	-7.7109	-45.1629	29.7410			

Method	Variances	DF	t Value	Pr > |t|
**Pooled**	Equal	62	-0.41	0.6821
**Satterthwaite**	Unequal	61.961	-0.41	0.6821

Equality of Variances				
**Method**	**Num DF**	**Den DF**	**F Value**	**Pr > F**
**Folded F**	31	31	1.05	0.8891

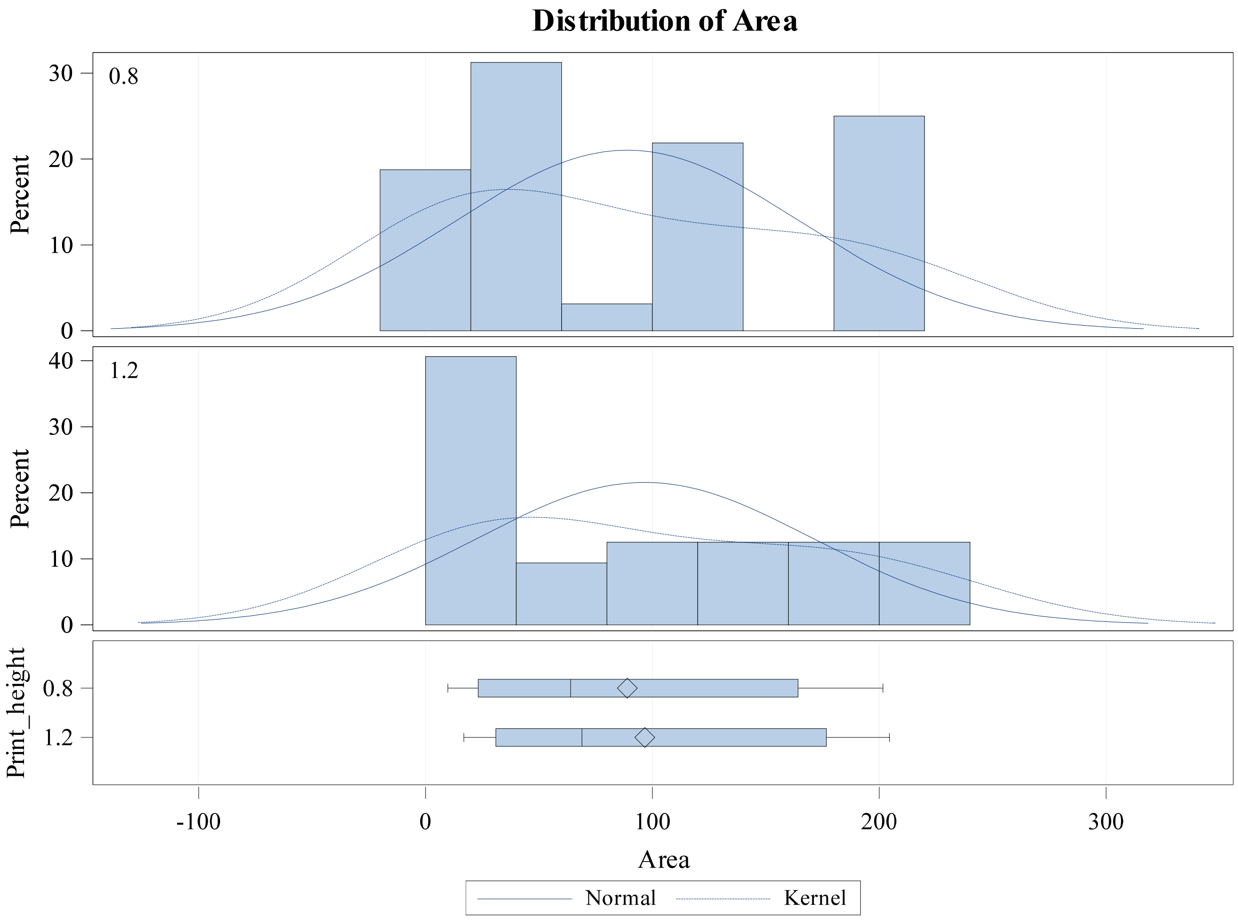

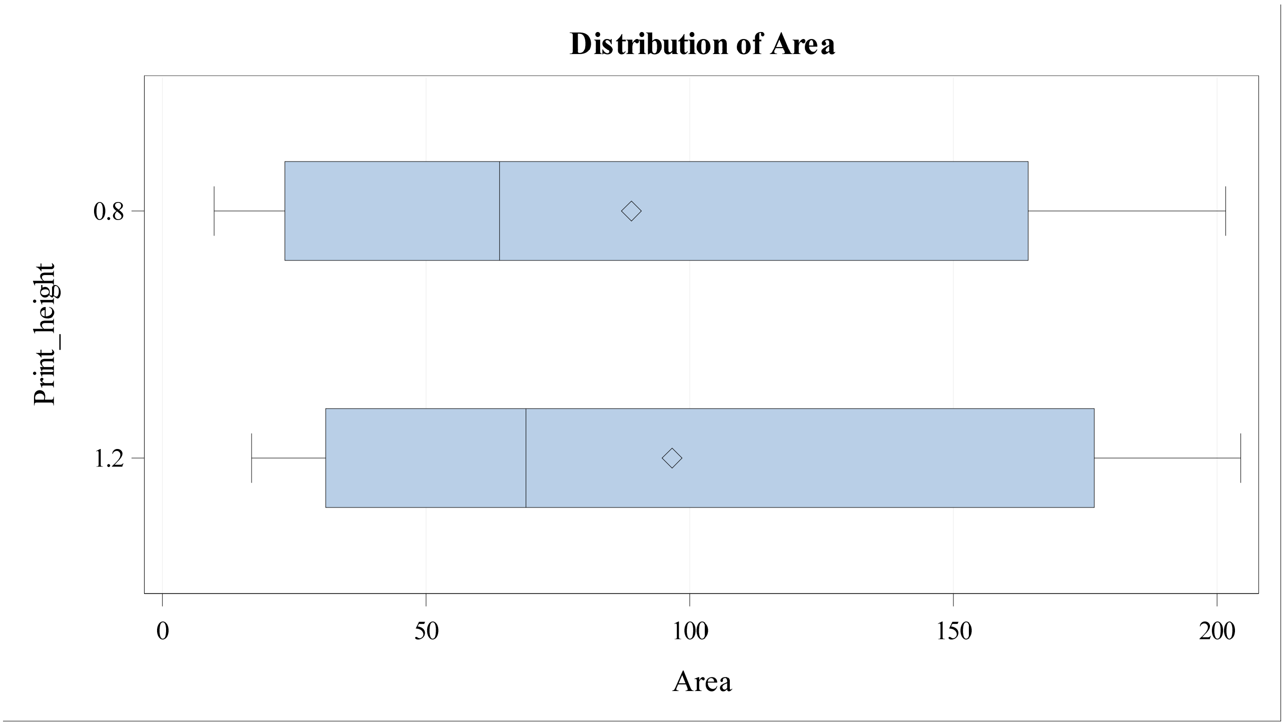

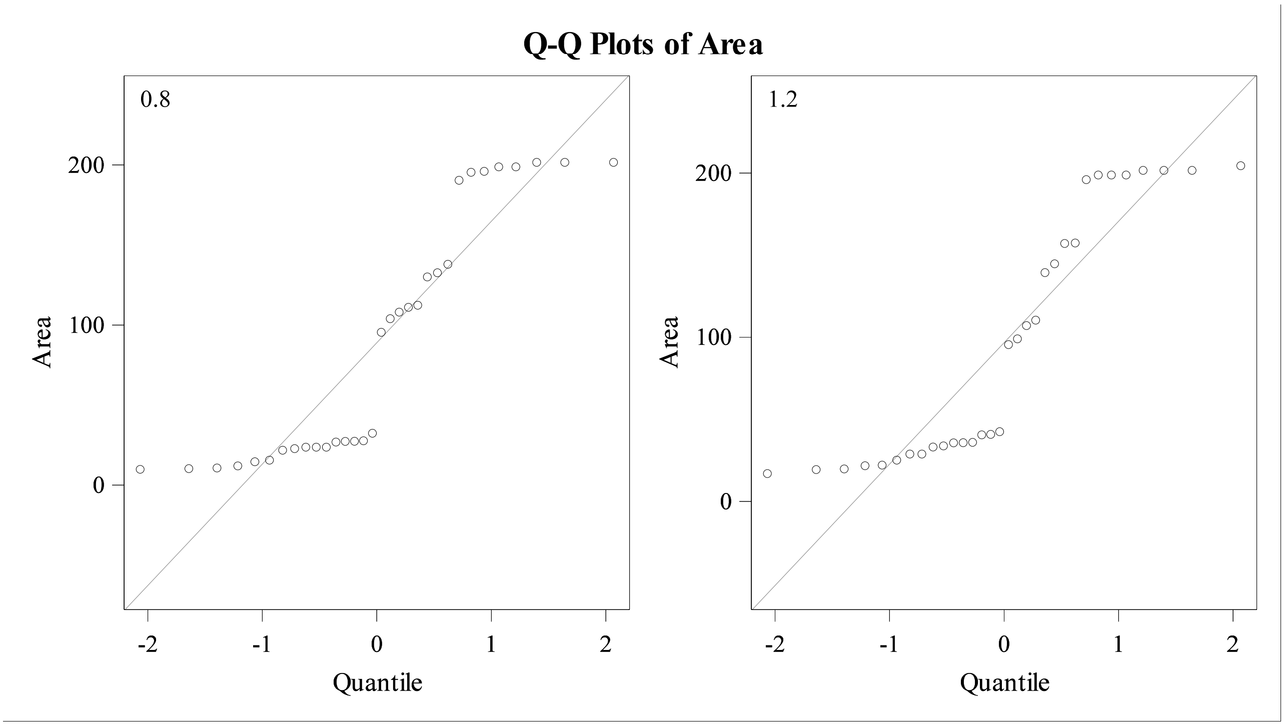

Variable: Area

Concentration=30

Print_height	Method	N	Mean	Std Dev	Std Err	Minimum	Maximum
**0.8**		32	119.7	64.6405	11.4269	34.0300	207.6
**1.2**		32	122.9	60.1021	10.6247	56.9400	208.8
**Diff (1-2)**	**Pooled**		-3.1444	62.4126	15.6031		

Print_height	Method	N	Mean	Std Dev	Std Err	Minimum	Maximum
Diff (1-2)	Satterthwaite		-3.1444		15.6031		

Print_height	Method	Mean	95% CL Mean		Std Dev	95% CL Std Dev	
0.8		119.7	96.4009	143.0	64.6405	51.8225	85.9382
1.2		122.9	101.2	144.5	60.1021	48.1841	79.9046
Diff (1-2)	Pooled	-3.1444	-34.3346	28.0459	62.4126	53.1000	75.7169
Diff (1-2)	Satterthwaite	-3.1444	-34.3379	28.0491			

Method	Variances	DF	t Value	Pr > |t|
Pooled	Equal	62	-0.20	0.8410
Satterthwaite	Unequal	61.674	-0.20	0.8410

Equality of Variances				
Method	Num DF	Den DF	F Value	Pr > F
Folded F	31	31	1.16	0.6878

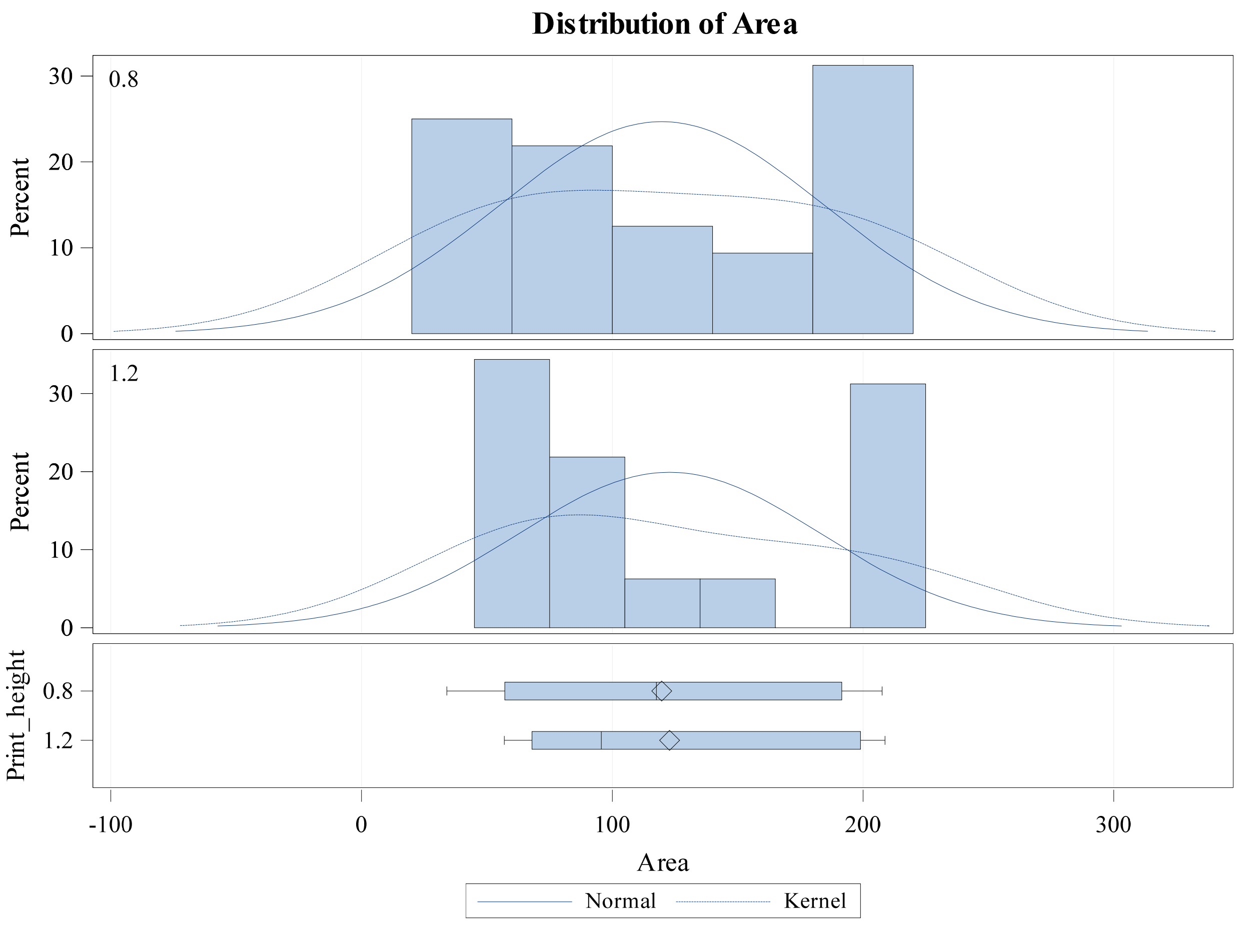

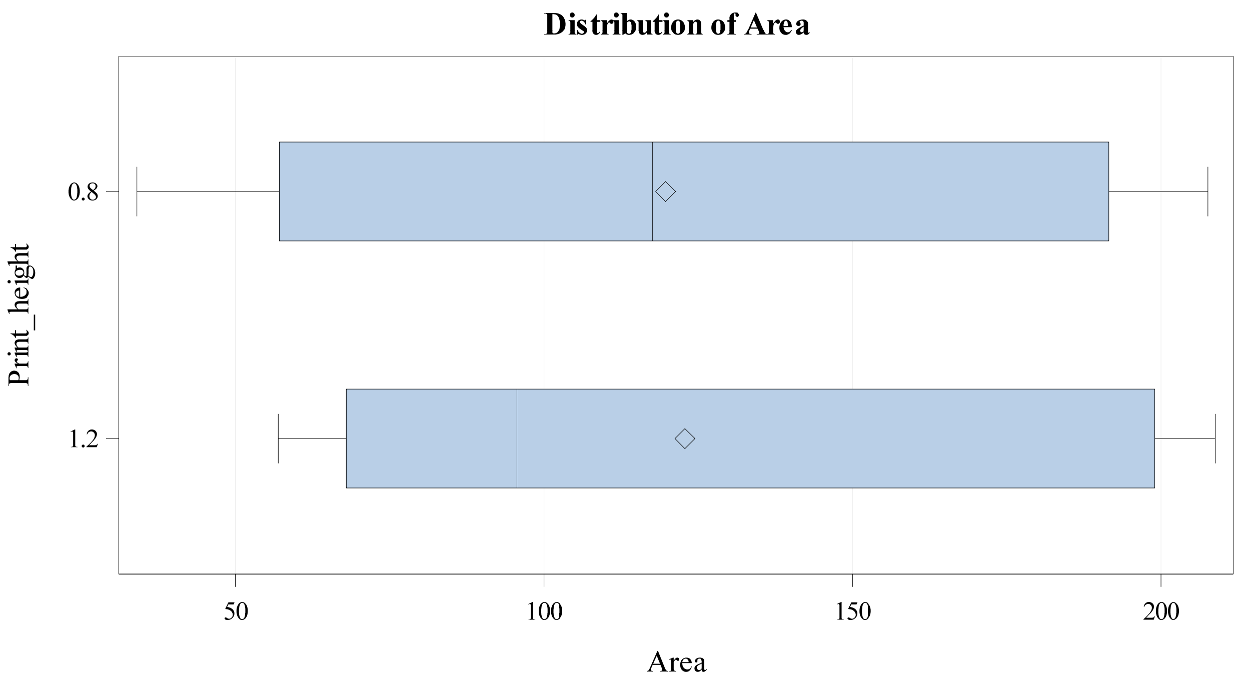

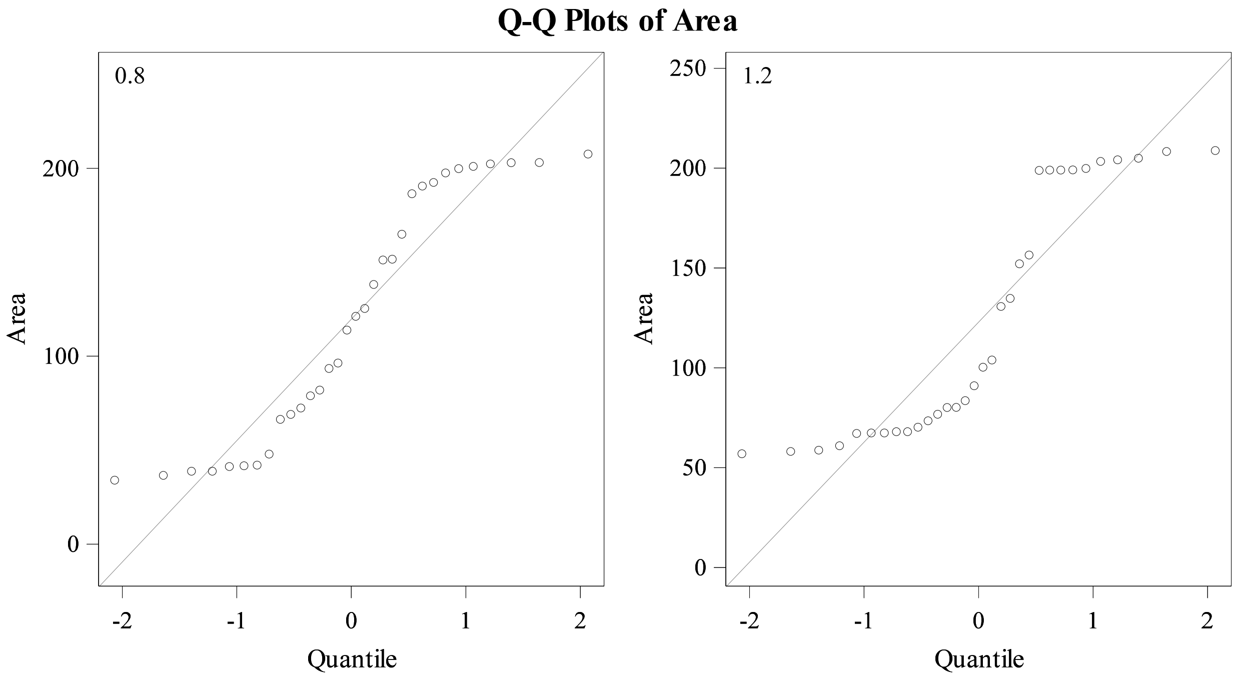

Biomechanical evaluation
Test of day by colagen concentration

Concentration=20

Class Level Information		
**Class**	**Levels**	**Values**
**Day**	5	0 1 3 5 7

**Number of Observations Read**	40
**Number of Observations Used**	40

Dependent Variable: Area

Concentration=20

Source	DF	Sum of Squares	Mean Square	F Value	Pr > F
**Model**	4	65492.61153	16373.15288	29.22	<.0001
**Error**	35	19614.80616	560.42303		
**Corrected Total**	39	85107.41770			

R-Square	Coeff Var	Root MSE	Area Mean
0.769529	20.11497	23.67326	117.6898

Source	DF	Anova SS	Mean Square	F Value	Pr > F
**Day**	4	65492.61153	16373.15288	29.22	<.0001

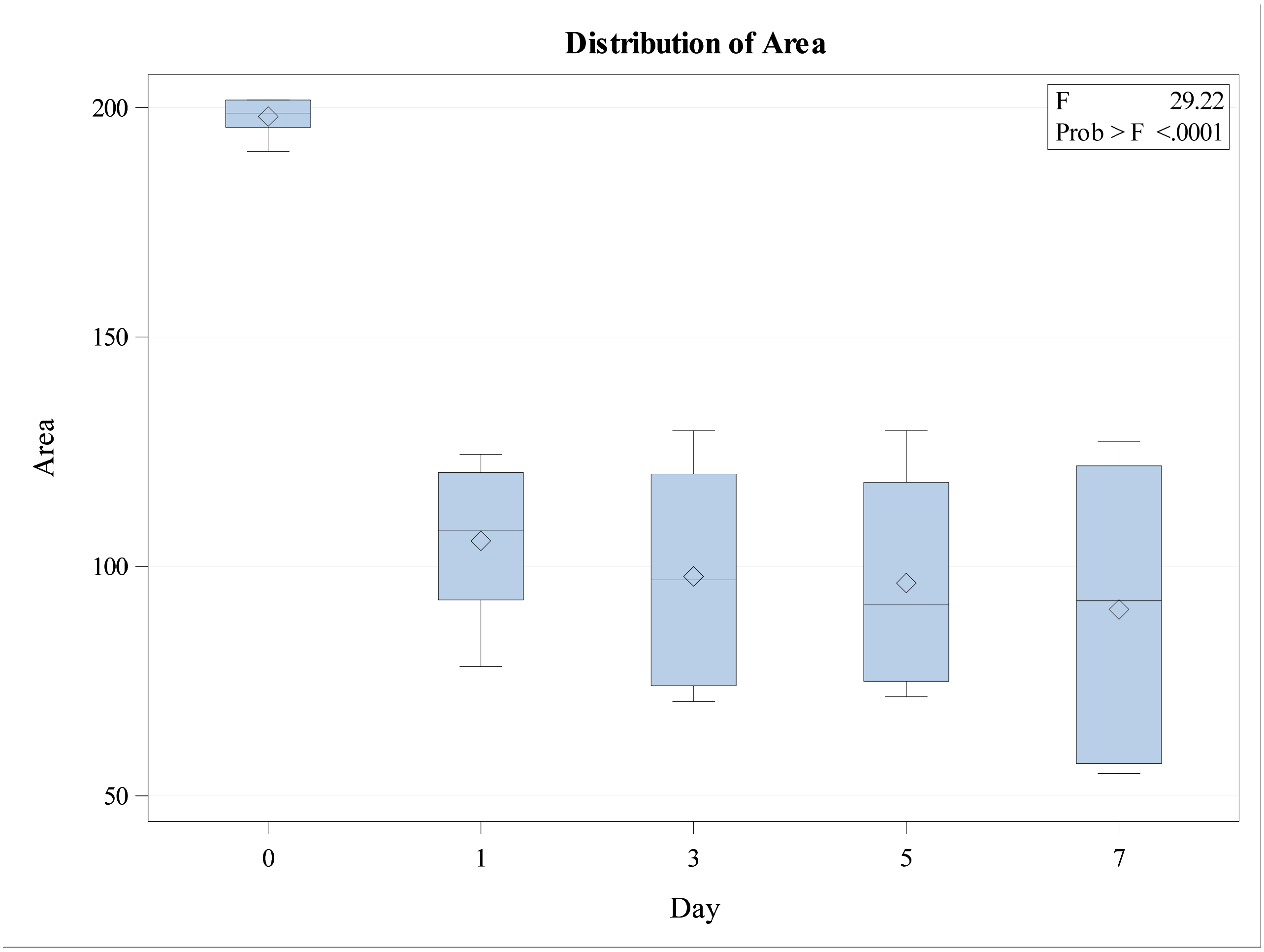

Concentration=30

Class Level Information		
**Class**	**Levels**	**Values**
**Day**	5	0 1 3 5 7

**Number of Observations Read**	40
**Number of Observations Used**	40

Dependent Variable: Area

Concentration=30

Source	DF	Sum of Squares	Mean Square	F Value	Pr > F
**Model**	4	8771.99462	2192.99866	6.59	0.0005
**Error**	35	11642.17938	332.63370		
**Corrected Total**	39	20414.17400			

R-Square	Coeff Var	Root MSE	Area Mean
0.429701	10.43169	18.23825	174.8350

Source	DF	Anova SS	Mean Square	F Value	Pr > F
**Day**	4	8771.994625	2192.998656	6.59	0.0005

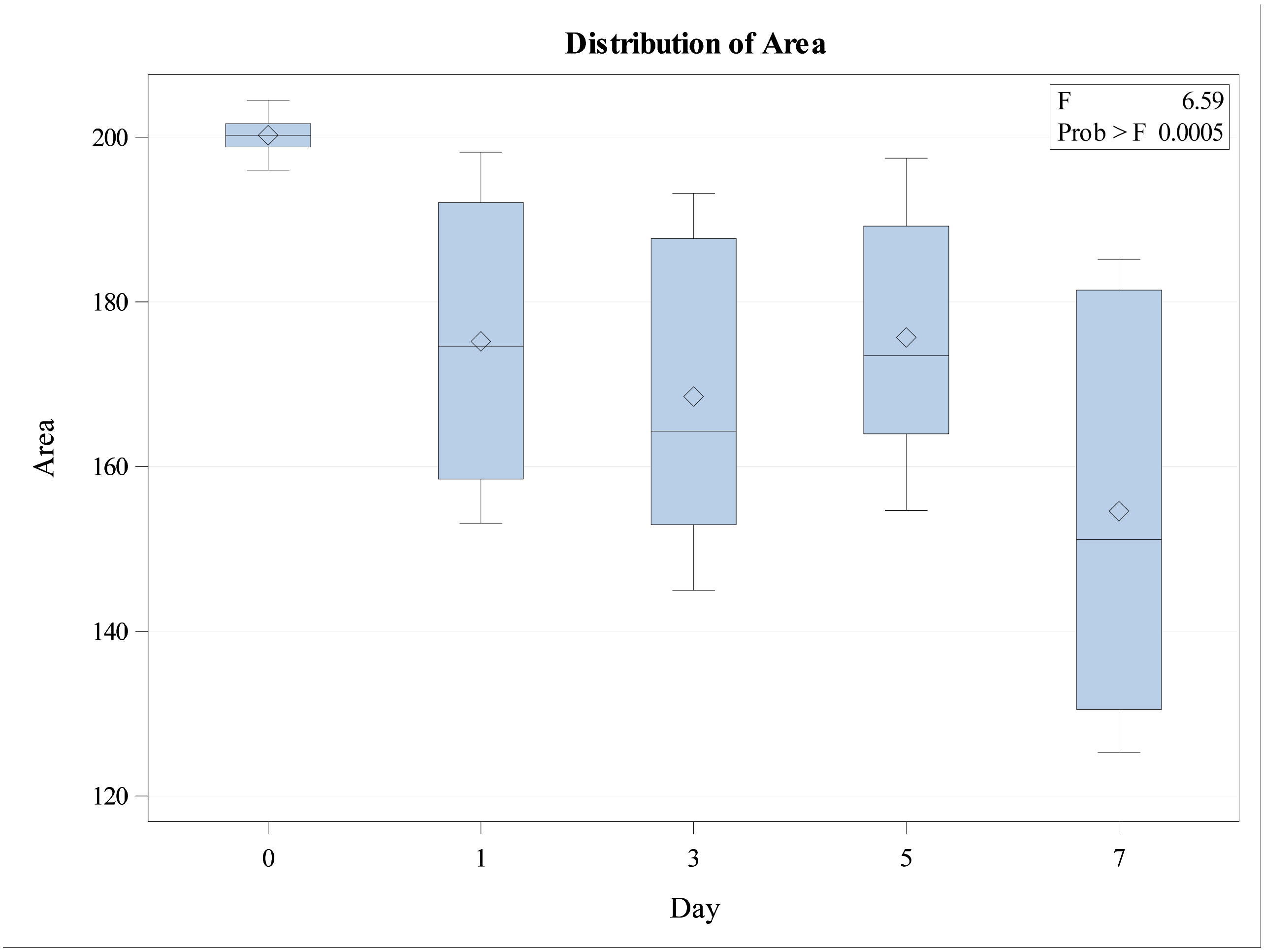

Biomechanical evaluation
Test of day by colagen concentration

Variable:
Area

Concentration=20

Cultivation	Method	N	Mean	Std Dev	Std Err	Minimum	Maximum
**D**		20	99.1900	51.6515	11.5496	54.8700	201.6
**S**		20	136.2	33.0296	7.3857	106.6	201.6
**Diff (1-2)**	**Pooled**		-36.9995	43.3522	13.7092		
**Diff (1-2)**	**Satterthwaite**		-36.9995		13.7092		

Cultivation	Method	Mean	95% CL Mean		Std Dev	95% CL Std Dev	
D		99.1900	75.0164	123.4	51.6515	39.2804	75.4407
S		136.2	120.7	151.6	33.0296	25.1187	48.2422
Diff (1-2)	Pooled	-36.9995	-64.7523	-9.2467	43.3522	35.4294	55.8714
Diff (1-2)	Satterthwaite	-36.9995	-64.9136	-9.0854			

Method	Variances	DF	t Value	Pr > |t|
Pooled	Equal	38	-2.70	0.0103
Satterthwaite	Unequal	32.313	-2.70	0.0110

Equality of Variances				
Method	Num DF	Den DF	F Value	Pr > F
Folded F	19	19	2.45	0.0583

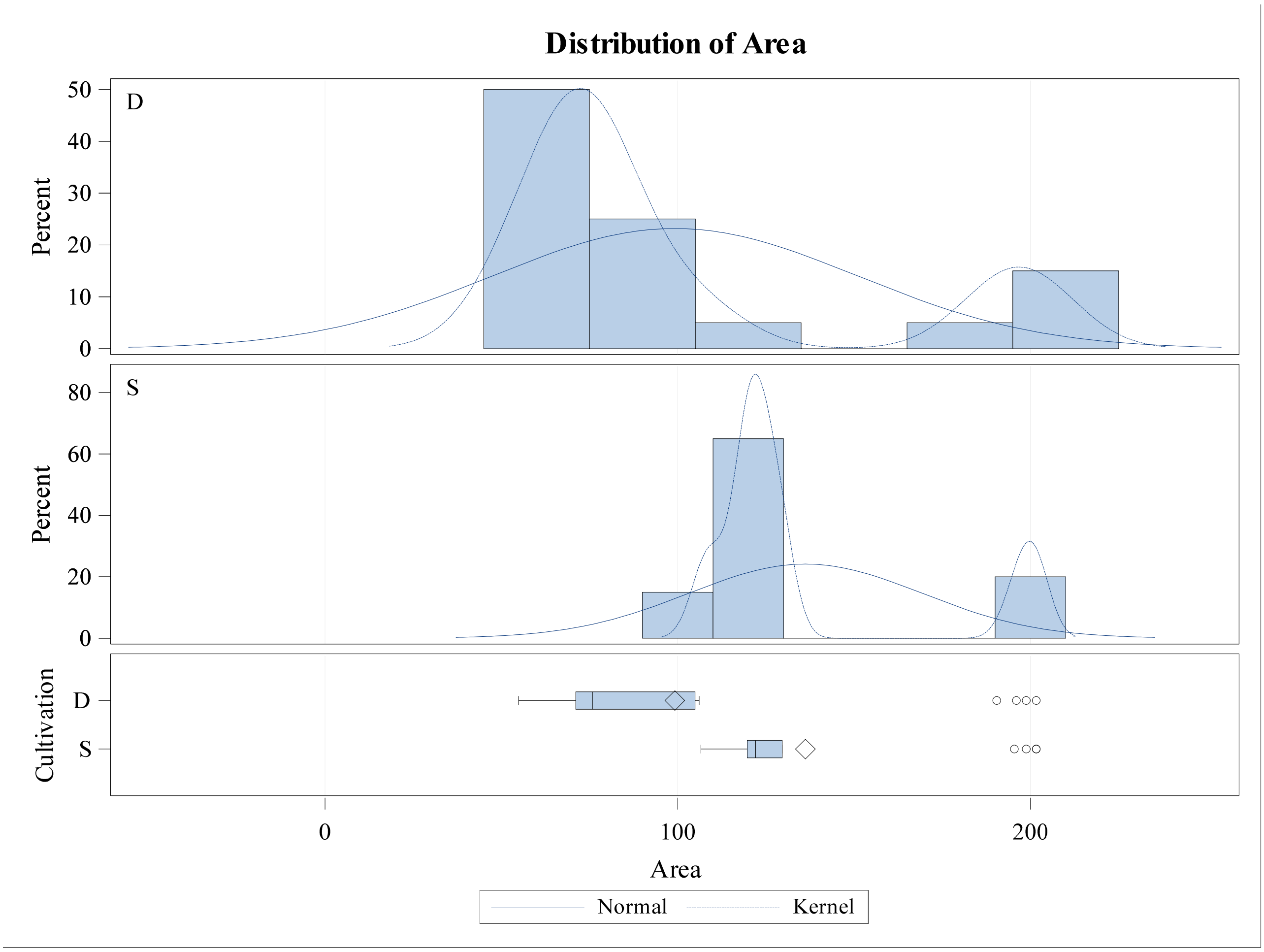

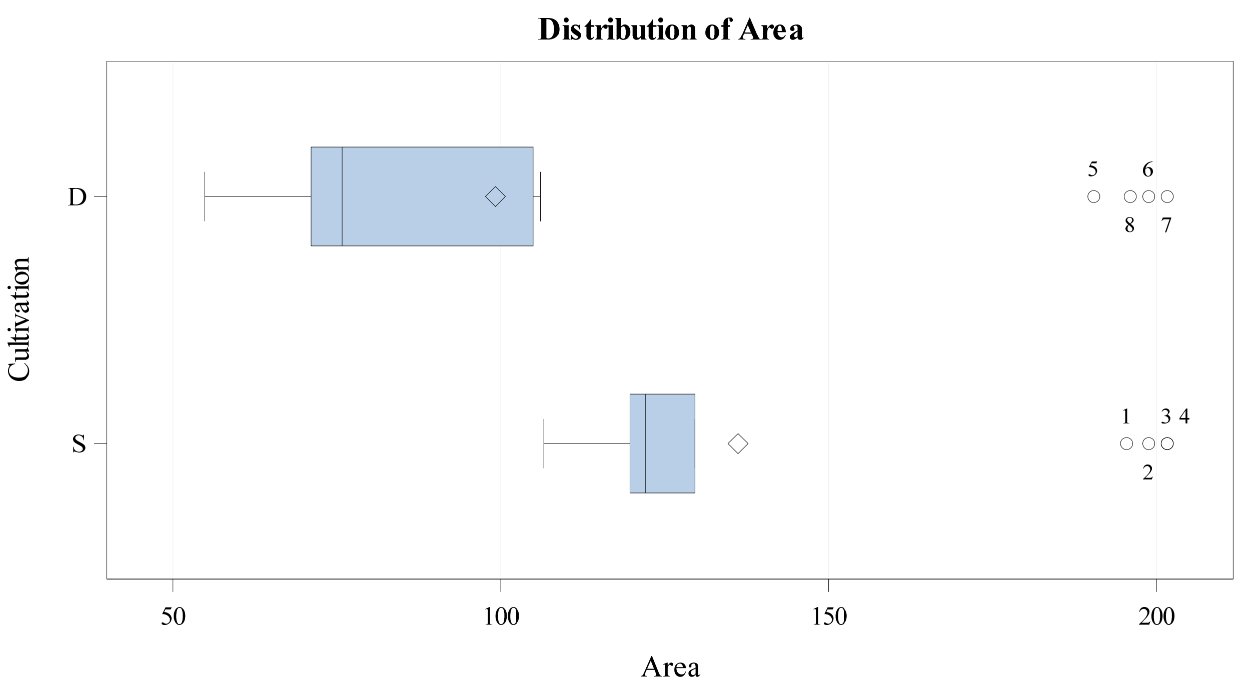

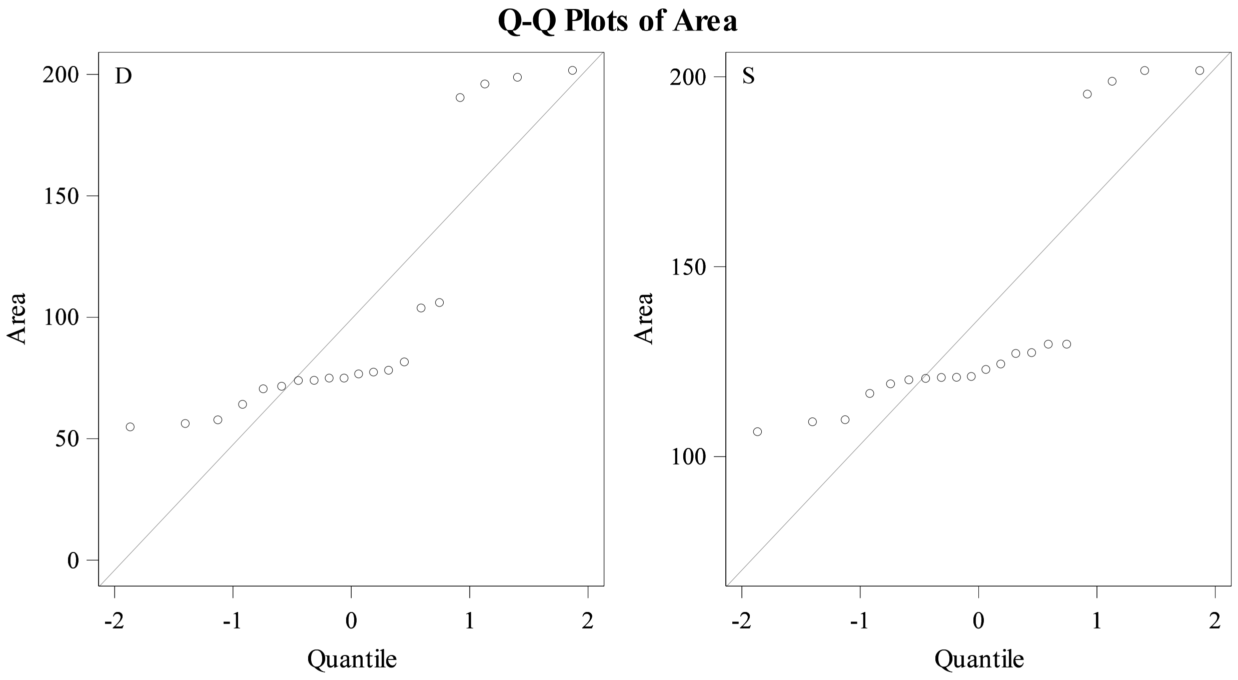

Variable: Area

Concentration=30

Cultivation	Method	N	Mean	Std Dev	Std Err	Minimum	Maximum
**D**		20	160.4	23.2004	5.1878	125.3	201.6
**S**		20	189.3	9.8511	2.2028	168.4	204.5
**Diff (1-2)**	**Pooled**		-28.8850	17.8228	5.6361		

Cultivation	Method	N	Mean	Std Dev	Std Err	Minimum	Maximum
Diff (1-2)	Satterthwaite		-28.8850		5.6361		

Cultivation	Method	Mean	95% CL Mean		Std Dev	95% CL Std Dev	
D		160.4	149.5	171.3	23.2004	17.6437	33.8858
S		189.3	184.7	193.9	9.8511	7.4917	14.3882
Diff (1-2)	Pooled	-28.8850	-40.2946	-17.4754	17.8228	14.5656	22.9696
Diff (1-2)	Satterthwaite	-28.8850	-40.4781	-17.2919			

Method	Variances	DF	t Value	Pr > |t|
Pooled	Equal	38	-5.13	<.0001
Satterthwaite	Unequal	25.635	-5.13	<.0001

Equality of Variances				
Method	Num DF	Den DF	F Value	Pr > F
Folded F	19	19	5.55	0.0005

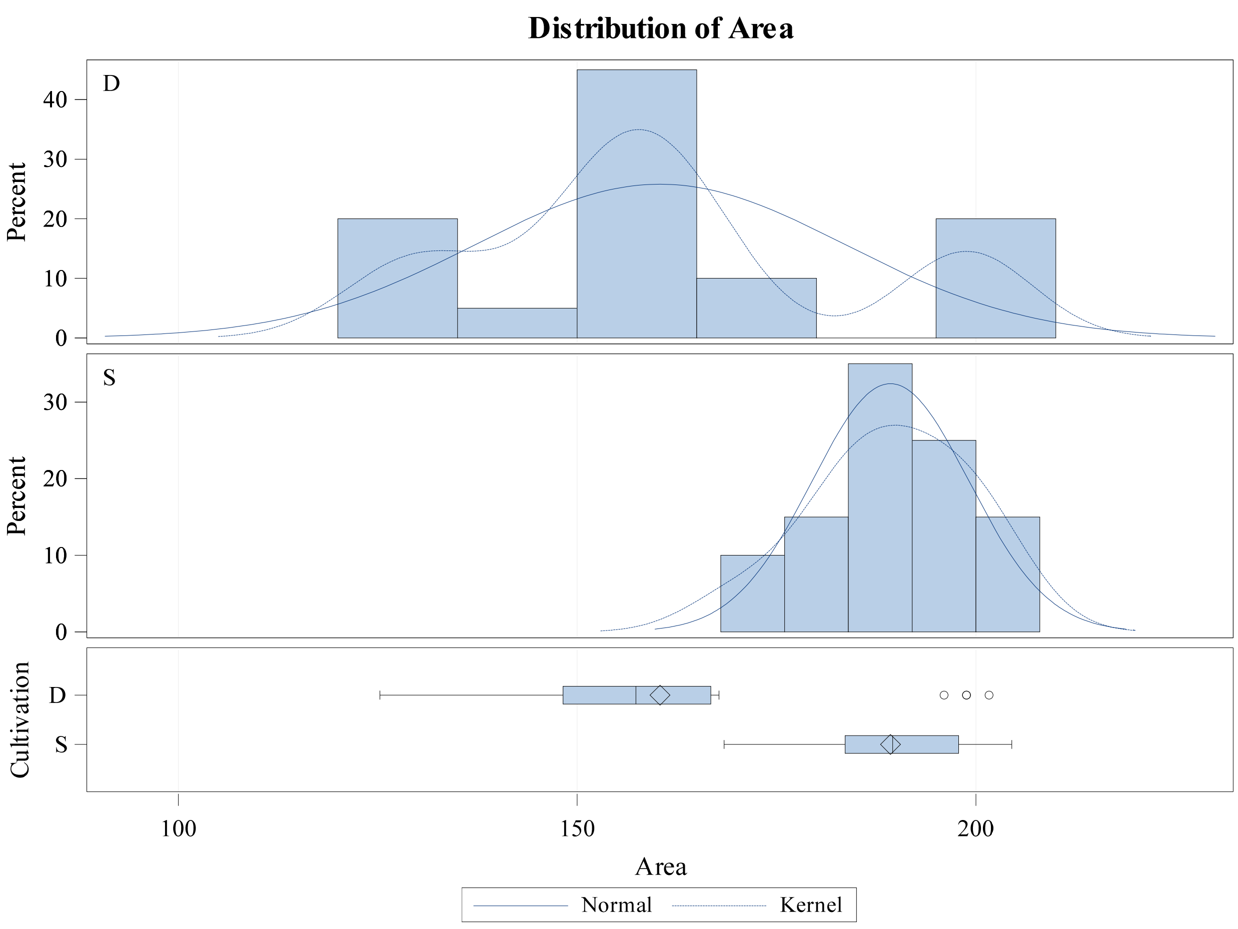

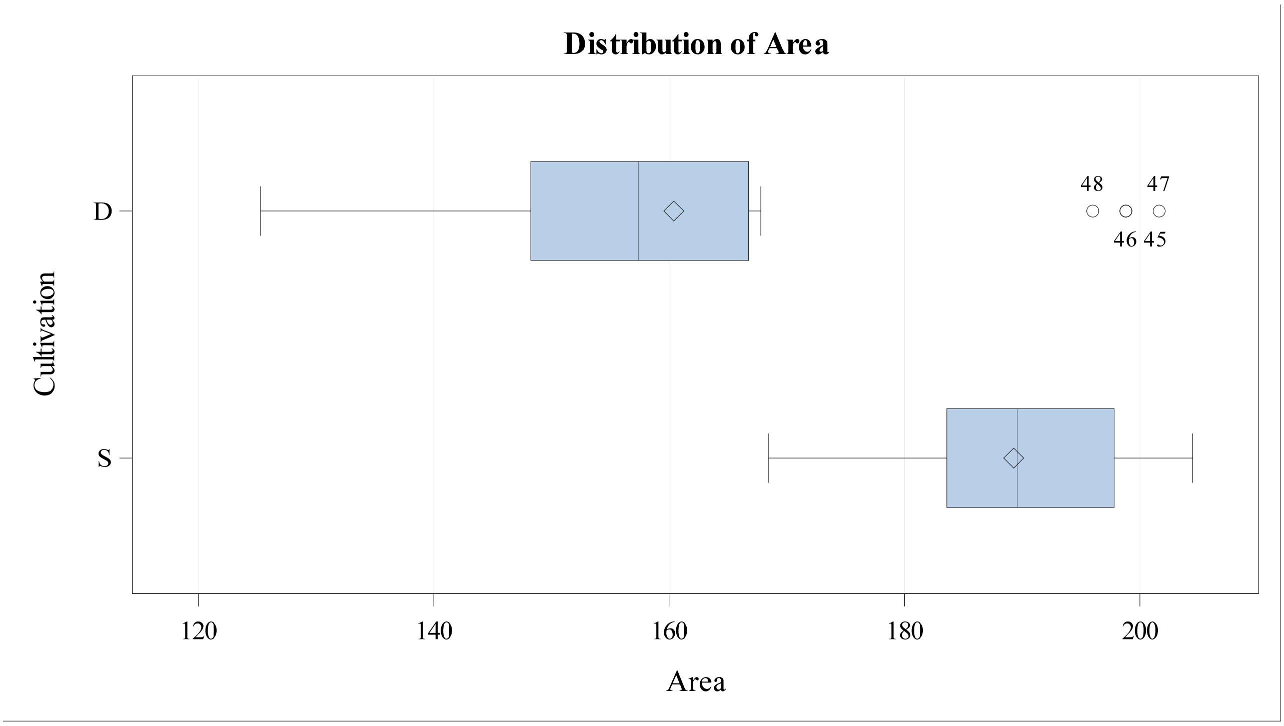

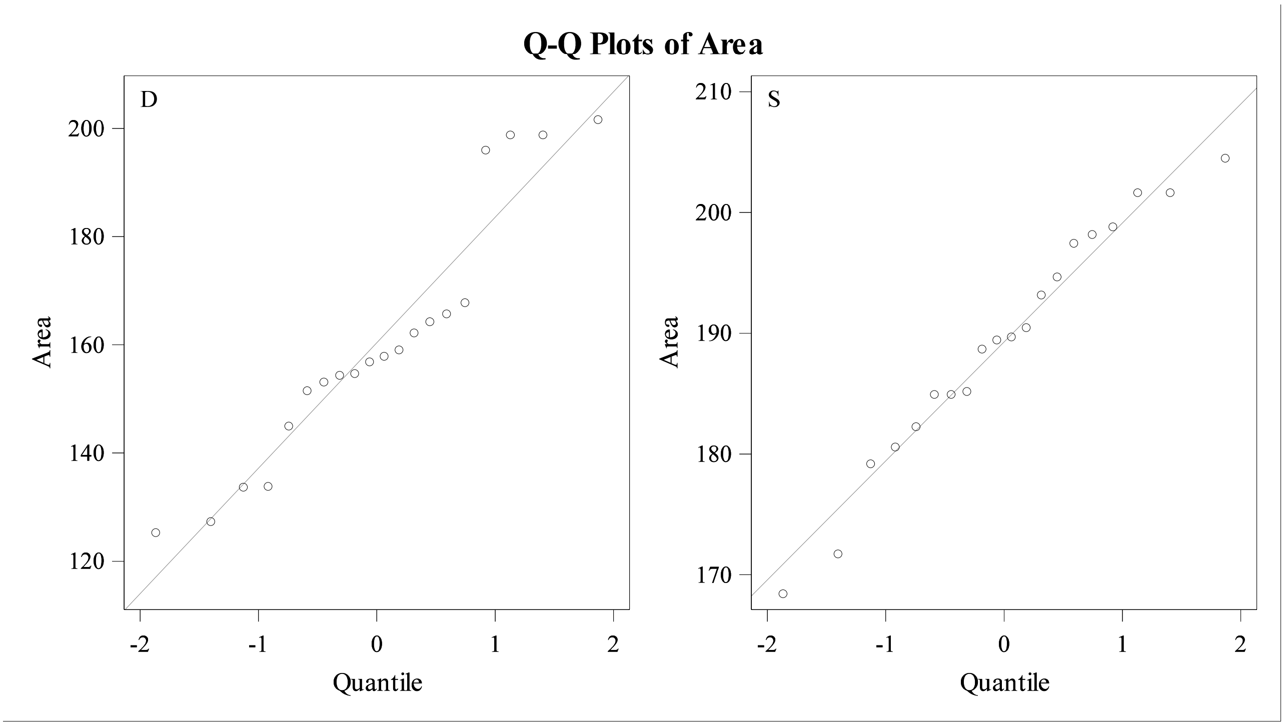

Biomechanical evaluation
Linear regression of area by colagen concentration

Model: MODEL1
Dependent Variable: Area

Concentration=20

**Number of Observations Read**	64

Number of Observations Used	64

Stepwise Selection: Step 1

Variable Day Entered: R-Square = 0.8147 and C(p) = 1.0390

Analysis of Variance					
Source	DF	Sum of Squares	Mean Square	F Value	Pr > F
Model	1	284452	284452	272.56	<.0001
Error	62	64705	1043.63325		
Corrected Total	63	349158			

Variable	Parameter Estimate	Standard Error	Type II SS	F Value	Pr > F
Intercept	170.91492	6.22045	787887	754.95	<.0001
Day	-34.71753	2.10290	284452	272.56	<.0001

Bounds on condition number: 1, 1

All variables left in the model are significant at the 0.1500 level.

No other variable met the 0.1500 significance level for entry into the model.

Summary of Stepwise Selection									
Step	Variable Entered	Variable Removed	Label	Number Vars In	Partial R-Square	Model R-Square	C(p)	F Value	Pr > F
1	Day			1	0.8147	0.8147	1.0390	272.56	<.0001

Model: MODEL1
Dependent Variable: Area

Concentration=20

Fit Diagnostics for Area

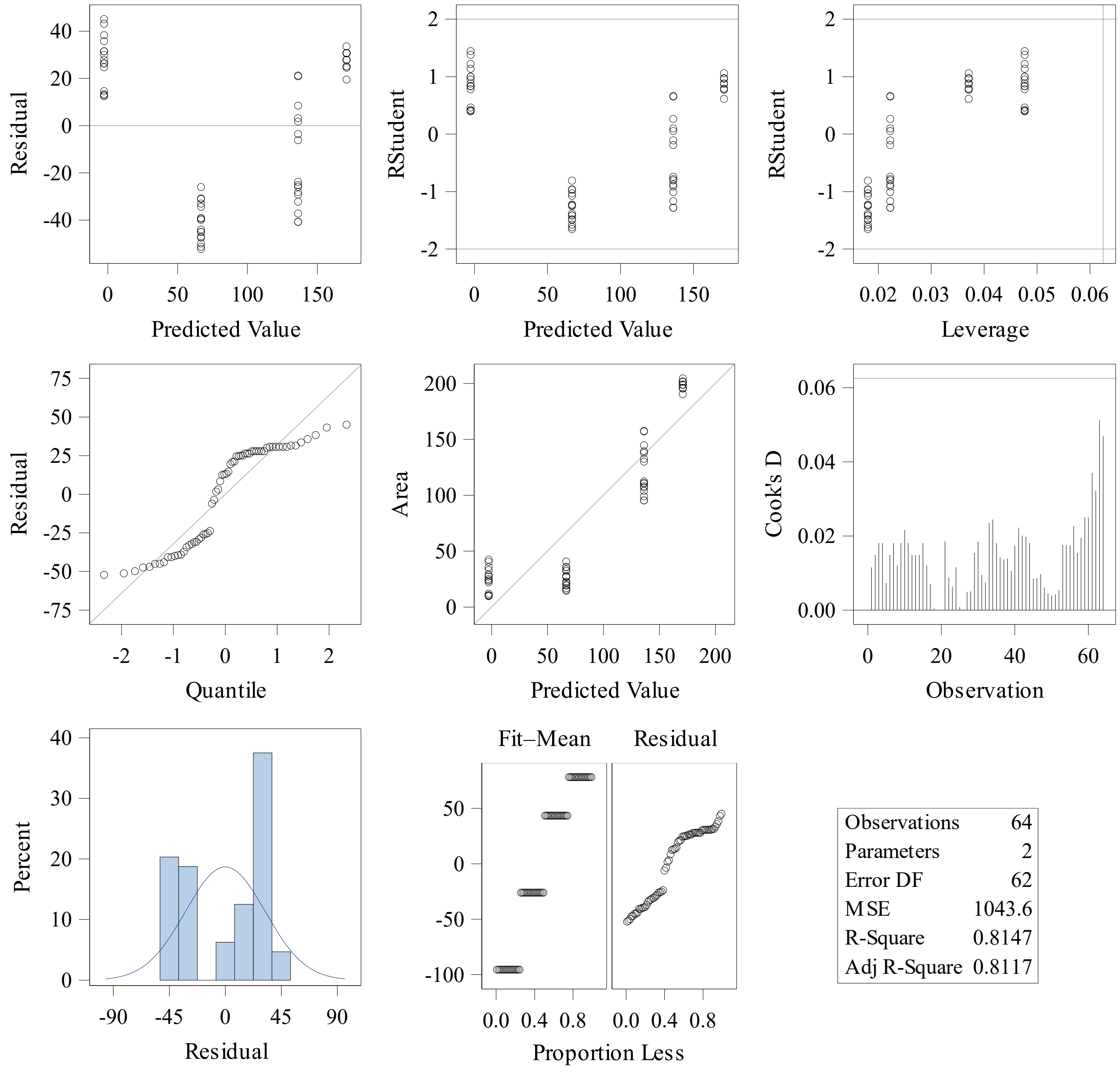

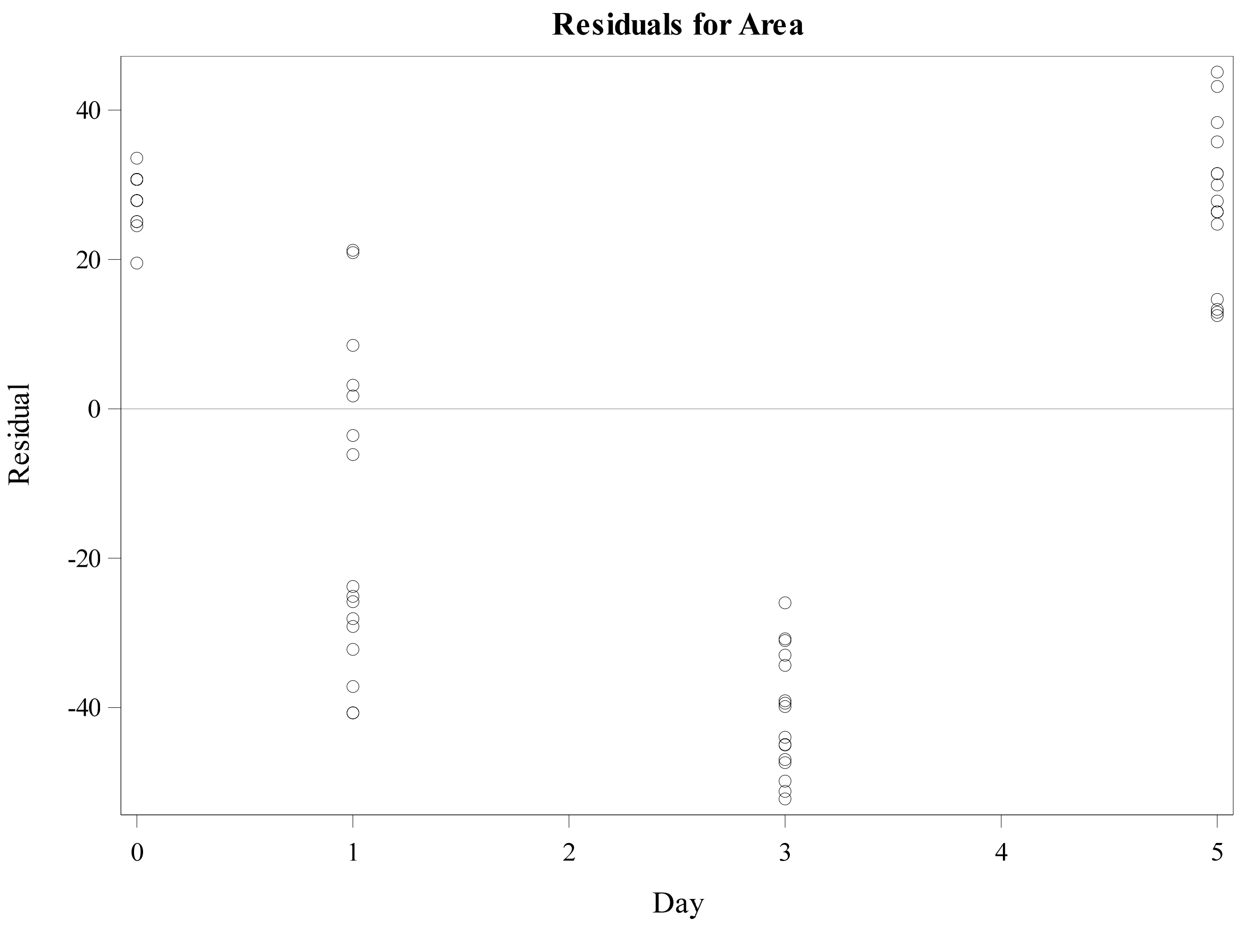

Residuals for Area
Residual
Day
40
20
0
-20
-40
0
1
2
3
4
5

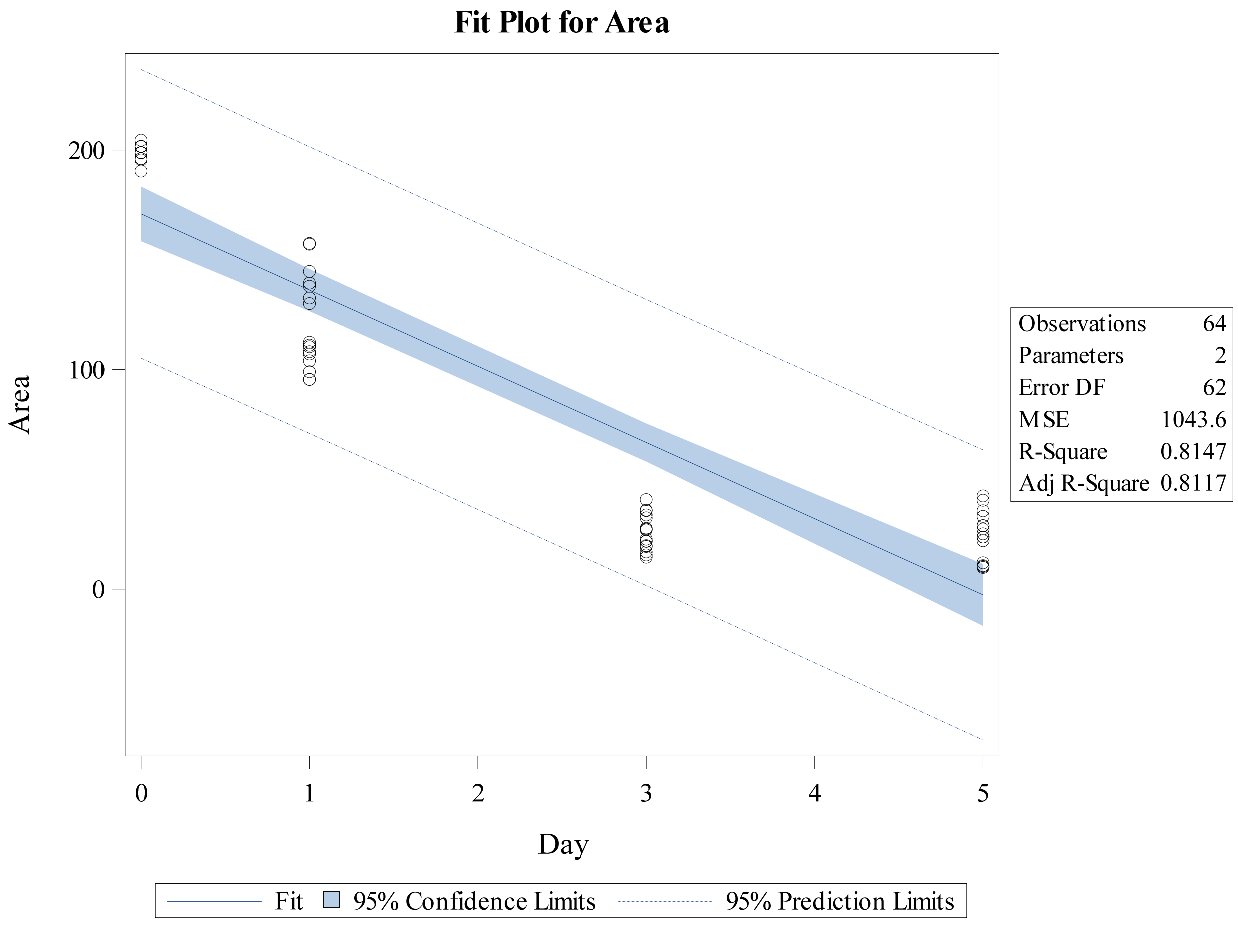

Fit Plot for Area
Area
Day
200
100
0
0
1
2
3
4
5
Observations 64
Parameters 2
Error DF 62
MSE 1043.6
R-Square 0.8147
Adj R-Square 0.8117
Fit
95% Confidence Limits
95% Prediction Limits

Model: MODEL1
Dependent Variable: Area

Concentration=30

**Number of Observations Read**	64
**Number of Observations Used**	64

Stepwise Selection: Step 1

Variable Day Entered: R-Square = 0.7269 and C(p) = 0.1631

**Analysis of Variance**					
**Source**	**DF**	**Sum of Squares**	**Mean Square**	**F Value**	**Pr > F**
**Model**	1	175675	175675	165.04	<.0001
**Error**	62	65994	1064.41800		
**Corrected Total**	63	241669			

**Variable**	**Parameter Estimate**	**Standard Error**	**Type II SS**	**F Value**	**Pr > F**
**Intercept**	182.66611	6.28209	899953	845.49	<.0001
**Day**	-27.28341	2.12373	175675	165.04	<.0001

Bounds on condition number: 1, 1

All variables left in the model are significant at the 0.1500 level.

No other variable met the 0.1500 significance level for entry into the model.

**Summary of Stepwise Selection**									
**Step**	**Variable Entered**	**Variable Removed**	**Label**	**Number Vars In**	**Partial R-Square**	**Model R-Square**	**C(p)**	**F Value**	**Pr > F**
**1**	Day			1	0.7269	0.7269	0.1631	165.04	<.0001

Model: MODEL1
Dependent Variable: Area

Concentration=30

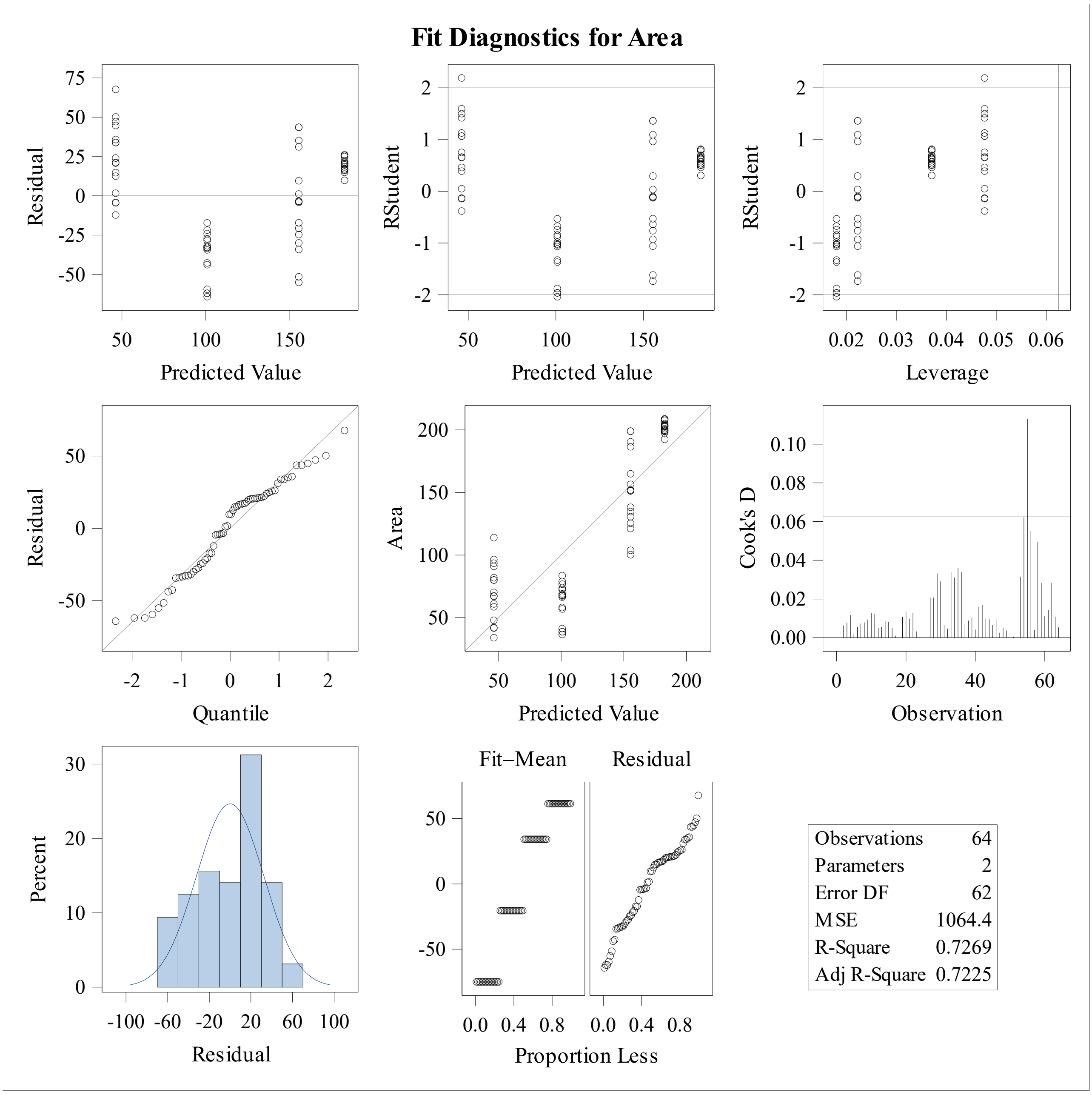

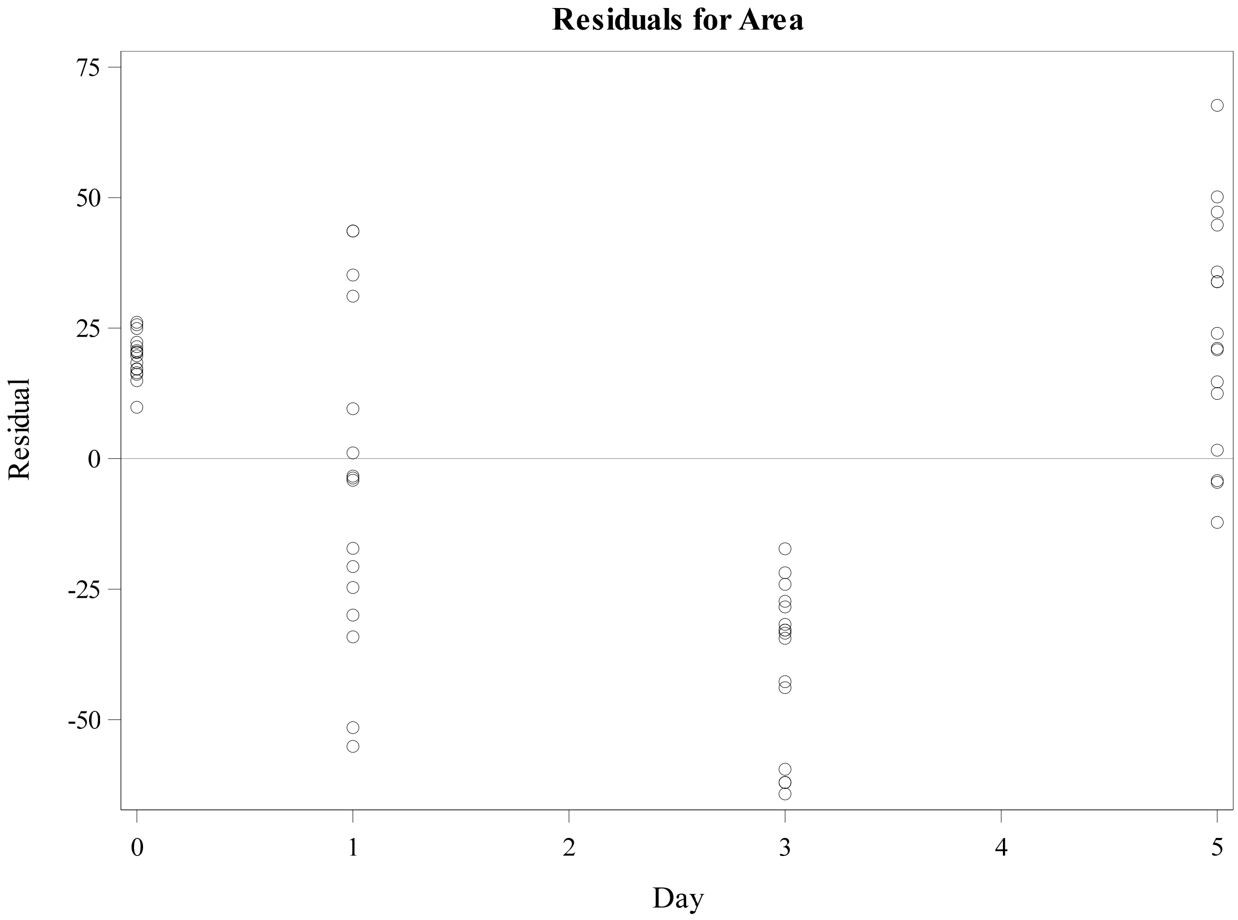

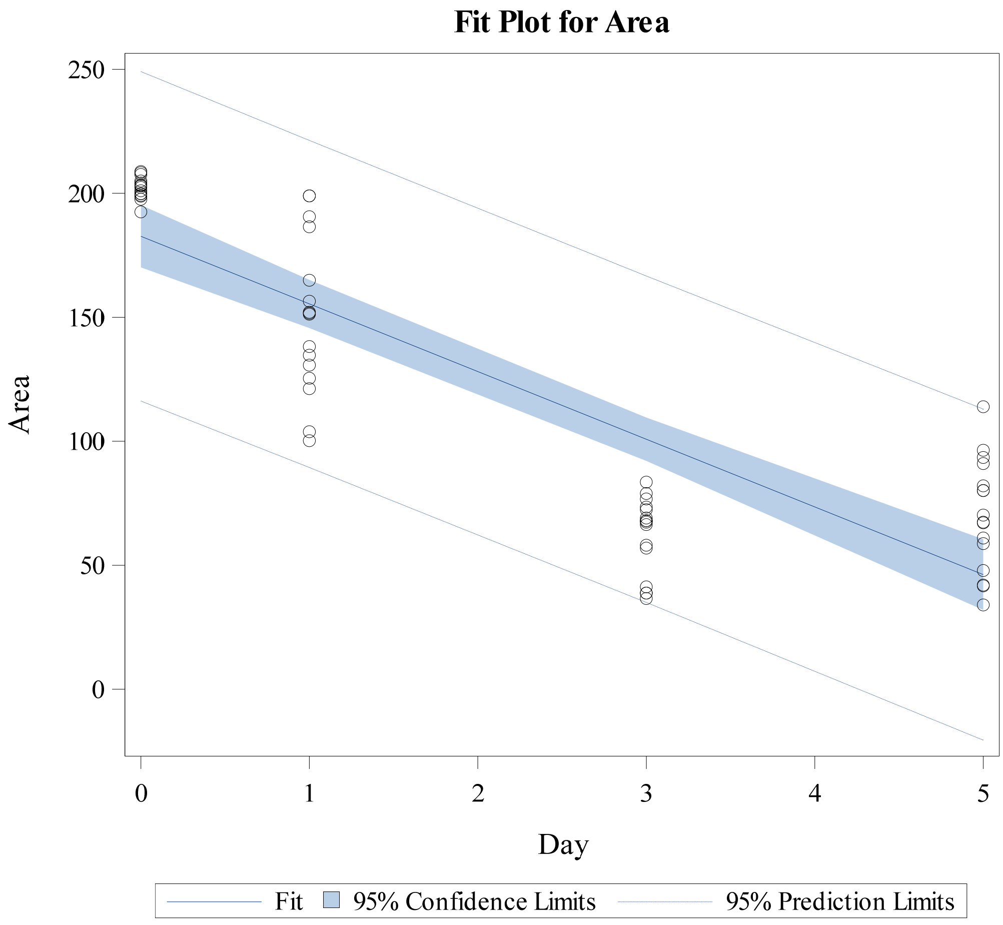

Observations	64
Parameters	2
Error DF	62
MSE	1064.4
R-Square	0.7269
Adj R-Square	0.7225

Additional evaluations
Proliferation medium
Linear Regression of Area by Day, Concentration a Cultivation

Model: MODEL1
Dependent Variable: Area Area

**Number of Observations Read**	80
**Number of Observations Used**	80

Stepwise Selection: Step 1

Variable Concentration Entered: R-Square = 0.3713 and C(p) = 89.0535

Analysis of Variance					
**Source**	**DF**	**Sum of Squares**	**Mean Square**	**F Value**	**Pr > F**
**Model**	1	64198	64198	46.07	<.0001
**Error**	78	108698	1393.55855		
**Corrected Total**	79	172896			

**Variable**	**Parameter Estimate**	**Standard Error**	**Type II SS**	**F Value**	**Pr > F**
**Intercept**	4.86700	21.28160	72.88520	0.05	0.8197
**Concentration**	5.66560	0.83473	64198	46.07	<.0001

Bounds on condition number: 1, 1

Stepwise Selection: Step 2

Variable Day Entered: R-Square = 0.5849 and C(p) = 34.9901

Analysis of Variance					
**Source**	**DF**	**Sum of Squares**	**Mean Square**	**F Value**	**Pr > F**
**Model**	2	101119	50560	54.24	<.0001
**Error**	77	71776	932.16142		
**Corrected Total**	79	172896			

Variable	Parameter Estimate	Standard Error	Type II SS	F Value	Pr > F
**Intercept**	31.70750	17.92041	2918.22923	3.13	0.0808
**Day**	-8.38766	1.33275	36921	39.61	<.0001
**Concentration**	5.66560	0.68270	64198	68.87	<.0001

Bounds on condition number: 1, 4

Stepwise Selection: Step 3

Variable Cultivation__static_1_dynamic_2_ Entered: R-Square = 0.7105 and C(p) = 4.0000

Analysis of Variance					
**Source**	**DF**	**Sum of Squares**	**Mean Square**	**F Value**	**Pr > F**
**Model**	3	122845	40948	62.18	<.0001
**Error**	76	50051	658.55942		
**Corrected Total**	79	172896			

Variable	Parameter Estimate	Standard Error	Type II SS	F Value	Pr > F
**Intercept**	81.14600	17.34848	14408	21.88	<.0001
**Day**	-8.38766	1.12021	36921	56.06	<.0001
**Concentration**	5.66560	0.57383	64198	97.48	<.0001
**Cultivation__static_1_dynamic_2_**	-32.95900	5.73829	21726	32.99	<.0001

Bounds on condition number: 1, 9

All variables left in the model are significant at the 0.1500 level.

All variables have been entered into the model.

Summary of Stepwise Selection									
**Step**	**Variable Entered**	**Variable Removed**	**Label**	**Number Vars In**	**Partial R-Square**	**Model R-Square**	**C(p)**	**F Value**	**Pr > F**
**1**	Concentration		Concentration	1	0.3713	0.3713	89.0535	46.07	<.0001
**2**	Day		Day	2	0.2135	0.5849	34.9901	39.61	<.0001
**3**	Cultivation__static_1_dynamic_2_		Cultivation	3	0.1257	0.7105	4.0000	32.99	<.0001

Model: MODEL1
Dependent Variable: Area Area

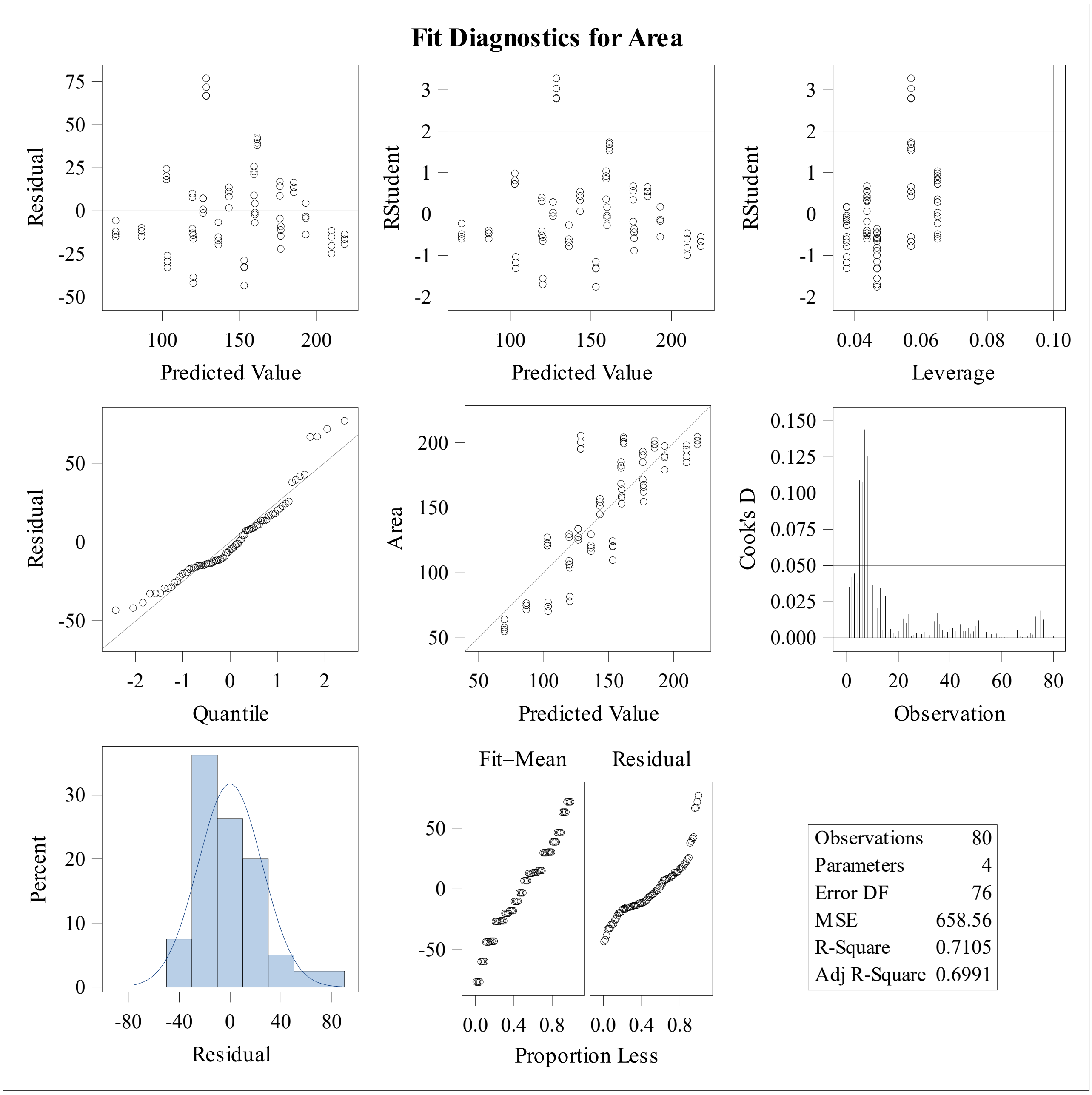

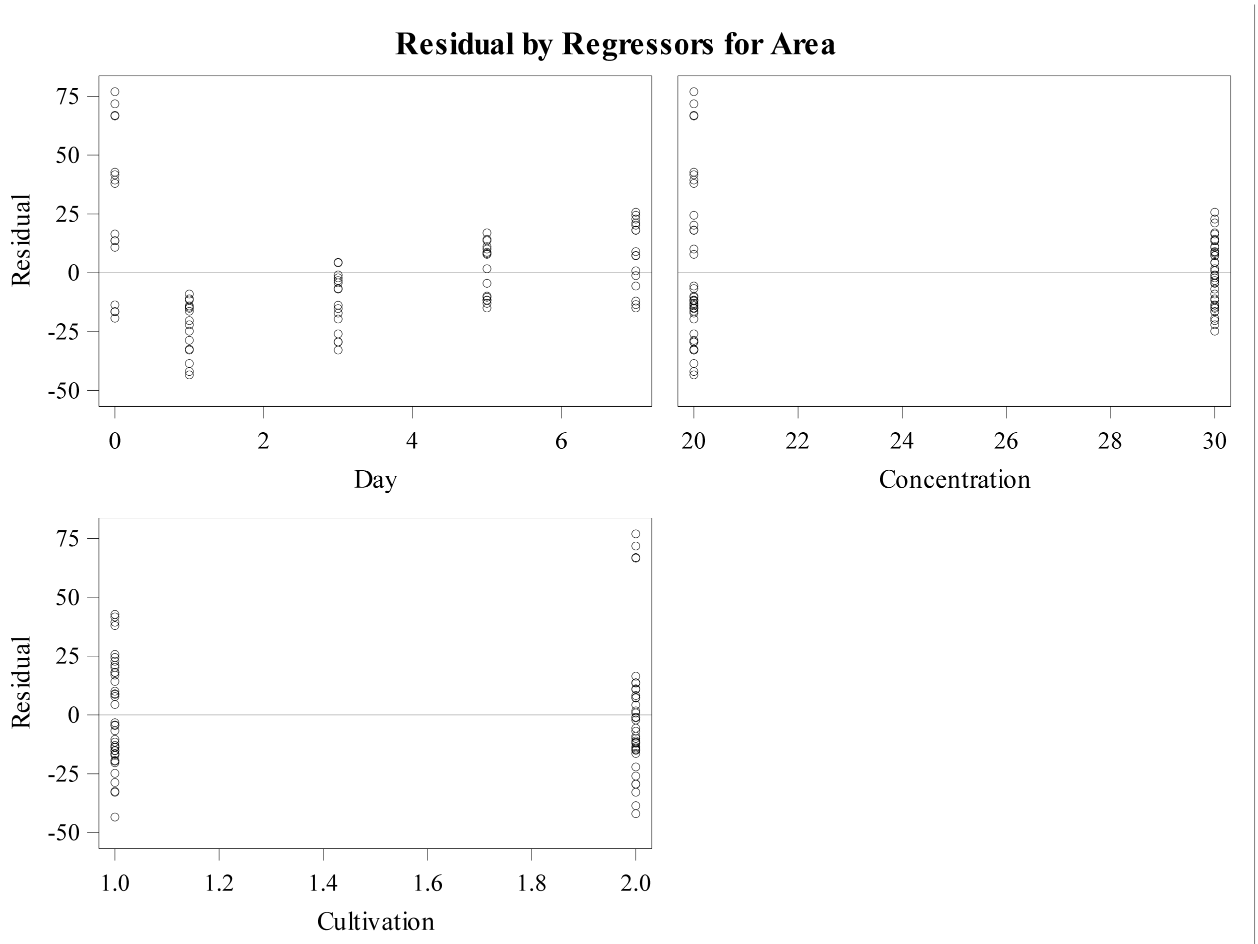

Additional evaluations
Proliferation medium
Linear Regression of Thickness by Day, Concentration a Cultivation

Model: MODEL1
Dependent Variable: Thickness Thickness

**Number of Observations Read**	80
**Number of Observations Used**	80

Stepwise Selection: Step 1

Variable Day Entered: R-Square = 0.4804 and C(p) = 72.6053

Analysis of Variance					
Source	DF	Sum of Squares	Mean Square	F Value	Pr > F
Model	1	0.39633	0.39633	72.11	<.0001
Error	78	0.42872	0.00550		
Corrected Total	79	0.82505			

Variable	Parameter Estimate	Standard Error	Type II SS	F Value	Pr > F
Intercept	1.19431	0.01326	44.55758	8106.70	<.0001
Day	-0.02748	0.00324	0.39633	72.11	<.0001

Bounds on condition number: 1, 1

Stepwise Selection: Step 2

Variable Cultivation__static_1_dynamic_2_ Entered: R-Square = 0.6326 and C(p) = 31.0651

Analysis of Variance					
Source	DF	Sum of Squares	Mean Square	F Value	Pr > F
Model	2	0.52194	0.26097	66.30	<.0001
Error	77	0.30311	0.00394		
Corrected Total	79	0.82505			

Variable	Parameter Estimate	Standard Error	Type II SS	F Value	Pr > F
Intercept	1.31319	0.02385	11.93301	3031.41	<.0001
Day	-0.02748	0.00274	0.39633	100.68	<.0001
Cultivation__static_1_dynamic_2_	-0.07925	0.01403	0.12561	31.91	<.0001

Bounds on condition number: 1, 4

Stepwise Selection: Step 3

Variable Concentration Entered: R-Square = 0.7343 and C(p) = 4.0000

Analysis of Variance					
Source	DF	Sum of Squares	Mean Square	F Value	Pr > F
Model	3	0.60579	0.20193	69.99	<.0001
Error	76	0.21926	0.00288		
Corrected Total	79	0.82505			

Variable	Parameter Estimate	Standard Error	Type II SS	F Value	Pr > F
Intercept	1.15131	0.03631	2.90041	1005.36	<.0001
Day	-0.02748	0.00234	0.39633	137.38	<.0001
Concentration	0.00648	0.00120	0.08385	29.07	<.0001
Cultivation__static_1_dynamic_2_	-0.07925	0.01201	0.12561	43.54	<.0001

Bounds on condition number: 1, 9

All variables left in the model are significant at the 0.1500 level.

All variables have been entered into the model.

Summary of Stepwise Selection									
Step	Variable Entered	Variable Removed	Label	Number Vars In	Partial R-Square	Model R-Square	C(p)	F Value	Pr > F
1	Day		Day	1	0.4804	0.4804	72.6053	72.11	<.0001
2	Cultivation__static_1_dynamic_2_		Cultivation	2	0.1522	0.6326	31.0651	31.91	<.0001
3	Concentration		Concentration	3	0.1016	0.7343	4.0000	29.07	<.0001

Model: MODEL1
Dependent Variable: Thickness Thickness

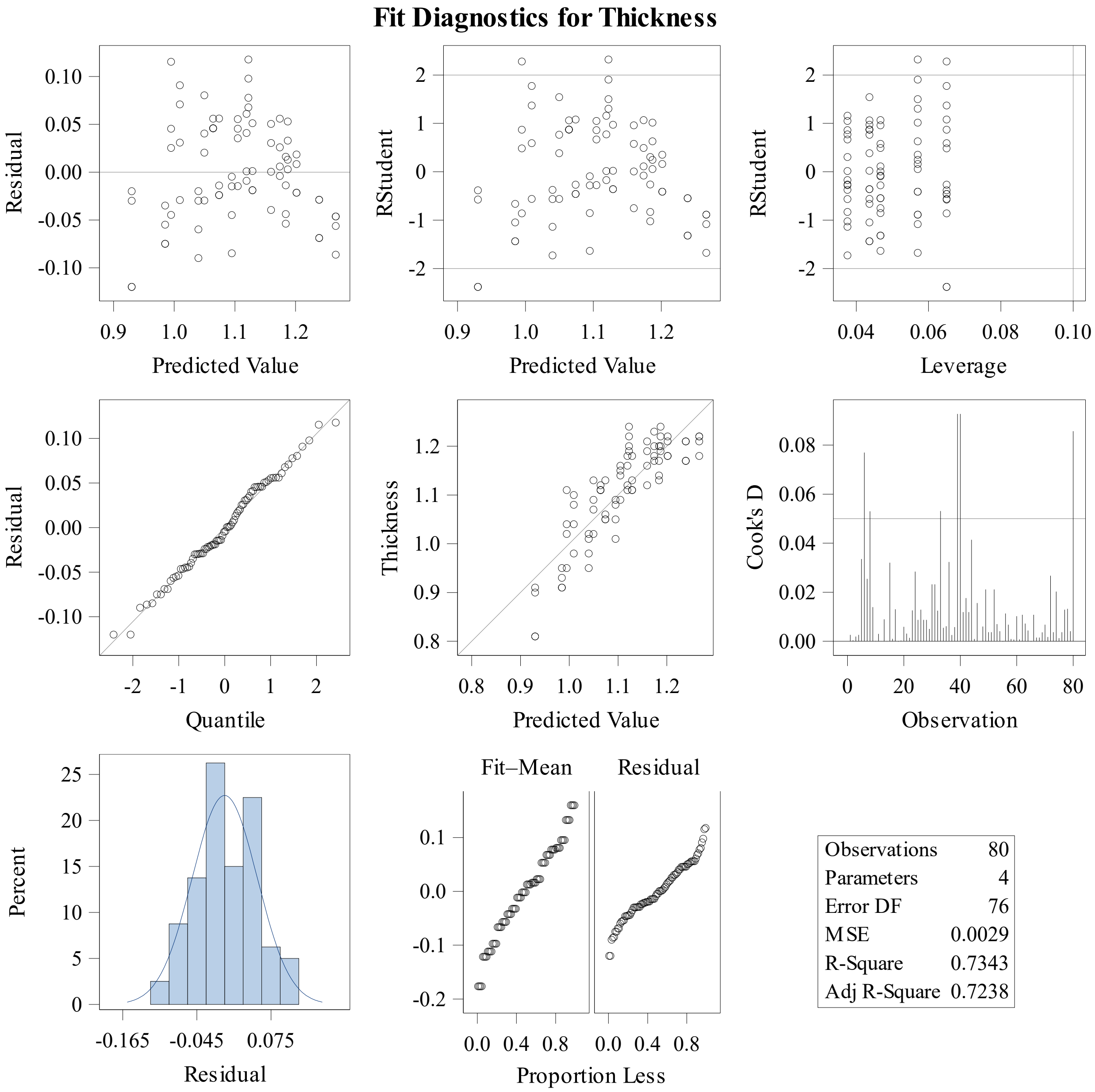
Fit Diagnostics for Thickness
Residual
Predicted Value
RStudent
Predicted Value
RStudent
Leverage
Residual
Quantile
Thickness
Predicted Value
Cook's D
Observation
Percent
Residual
Fit–Mean
Residual
Proportion Less
Observations 80
Parameters 4
Error DF 76
MSE 0.0029
R-Square 0.7343
Adj R-Square 0.7238

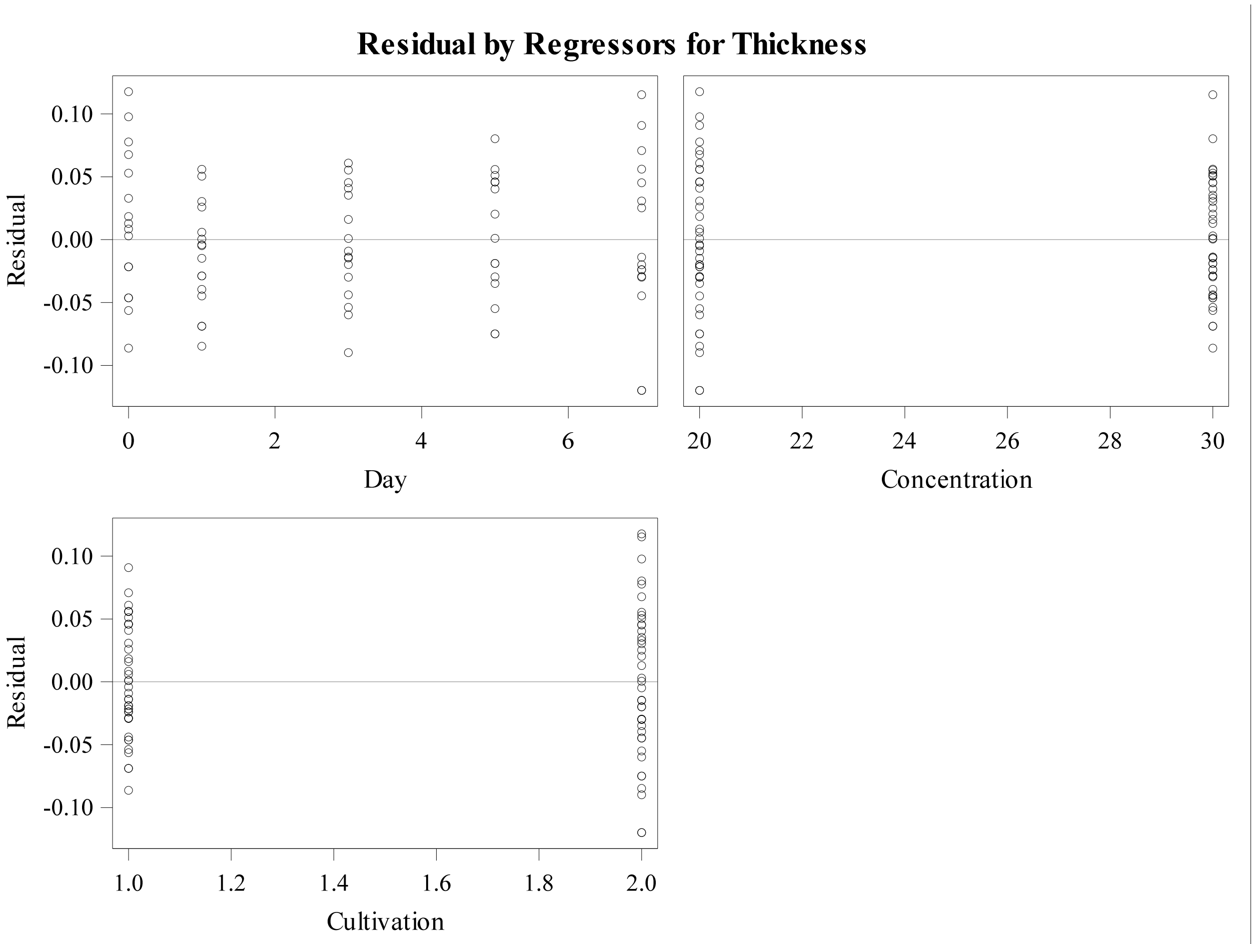

Additional evaluations
Differentaiation medium SMC
Linear Regression of Area by Concentration, Day, Print Height and Cultivation

Model: MODEL1
Dependent Variable: Area Area

**Number of Observations Read**	128
**Number of Observations Used**	128

Stepwise Selection: Step 1

Variable Day Entered: R-Square = 0.7354 and C(p) = 23.4268

Analysis of Variance					
Source	DF	Sum of Squares	Mean Square	F Value	Pr > F
Model	1	453606	453606	350.27	<.0001
Error	126	163172	1295.01916		
Corrected Total	127	616778			

Variable	Parameter Estimate	Standard Error	Type II SS	F Value	Pr > F
Intercept	176.79051	4.89972	1685978	1301.89	<.0001
Day	-31.00047	1.65641	453606	350.27	<.0001

Bounds on condition number: 1, 1

Stepwise Selection: Step 2

Variable Concentration Entered: R-Square = 0.7775 and C(p) = 1.9792

Analysis of Variance					
Source	DF	Sum of Squares	Mean Square	F Value	Pr > F
Model	2	479558	239779	218.42	<.0001
Error	125	137221	1097.76467		
Corrected Total	127	616778			

Variable	Parameter Estimate	Standard Error	Type II SS	F Value	Pr > F
Intercept	105.59559	15.32180	52141	47.50	<.0001
Concentration	2.84780	0.58571	25952	23.64	<.0001
Day	-31.00047	1.52505	453606	413.21	<.0001

Bounds on condition number: 1, 4

All variables left in the model are significant at the 0.1500 level.

No other variable met the 0.1500 significance level for entry into the model.

Summary of Stepwise Selection									
Step	Variable Entered	Variable Removed	Label	Number Vars In	Partial R-Square	Model R-Square	C(p)	F Value	Pr > F
1	Day		Day	1	0.7354	0.7354	23.4268	350.27	<.0001
2	Concentration		Concentration	2	0.0421	0.7775	1.9792	23.64	<.0001

Model: MODEL1
Dependent Variable: Area Area

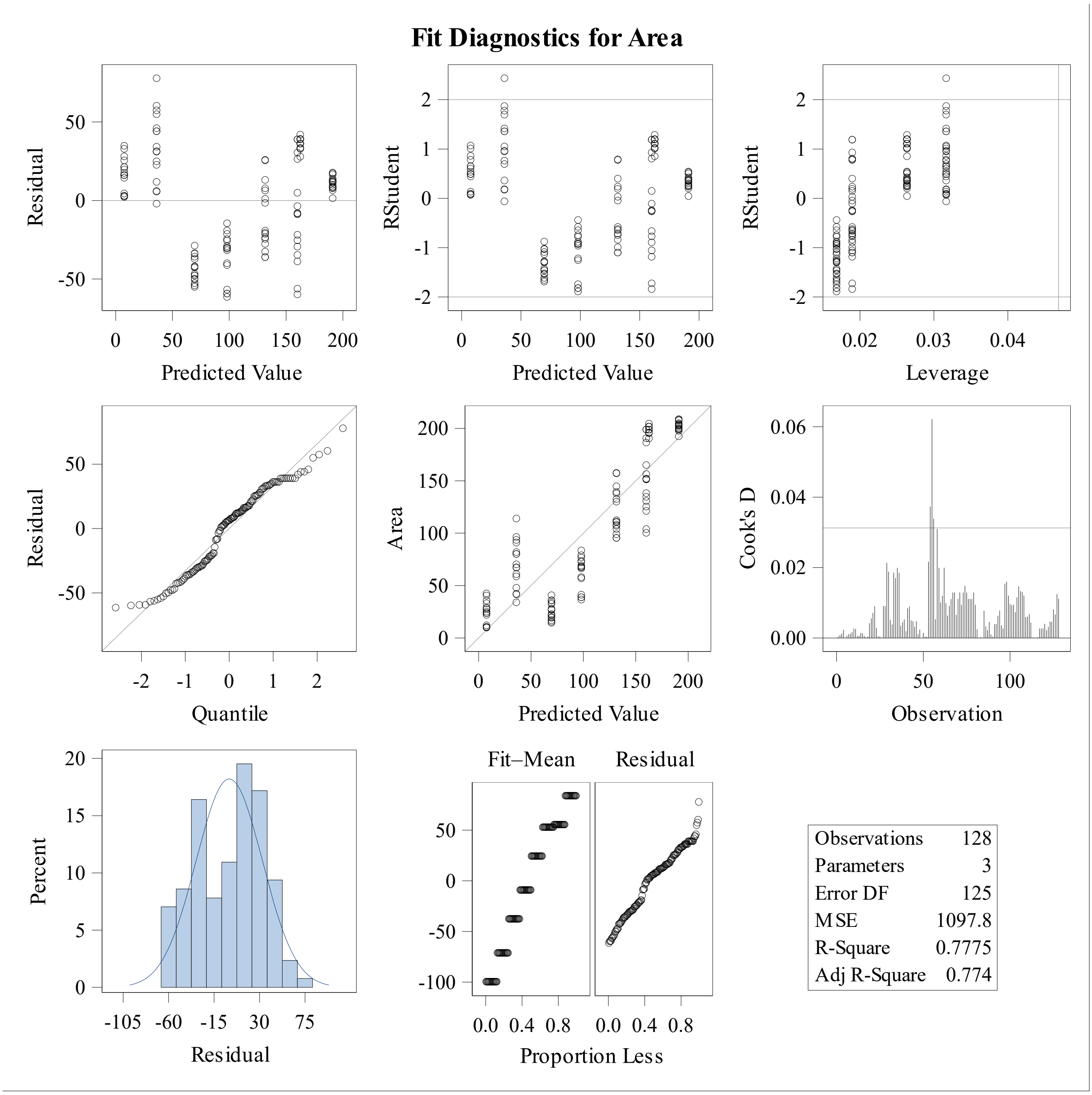

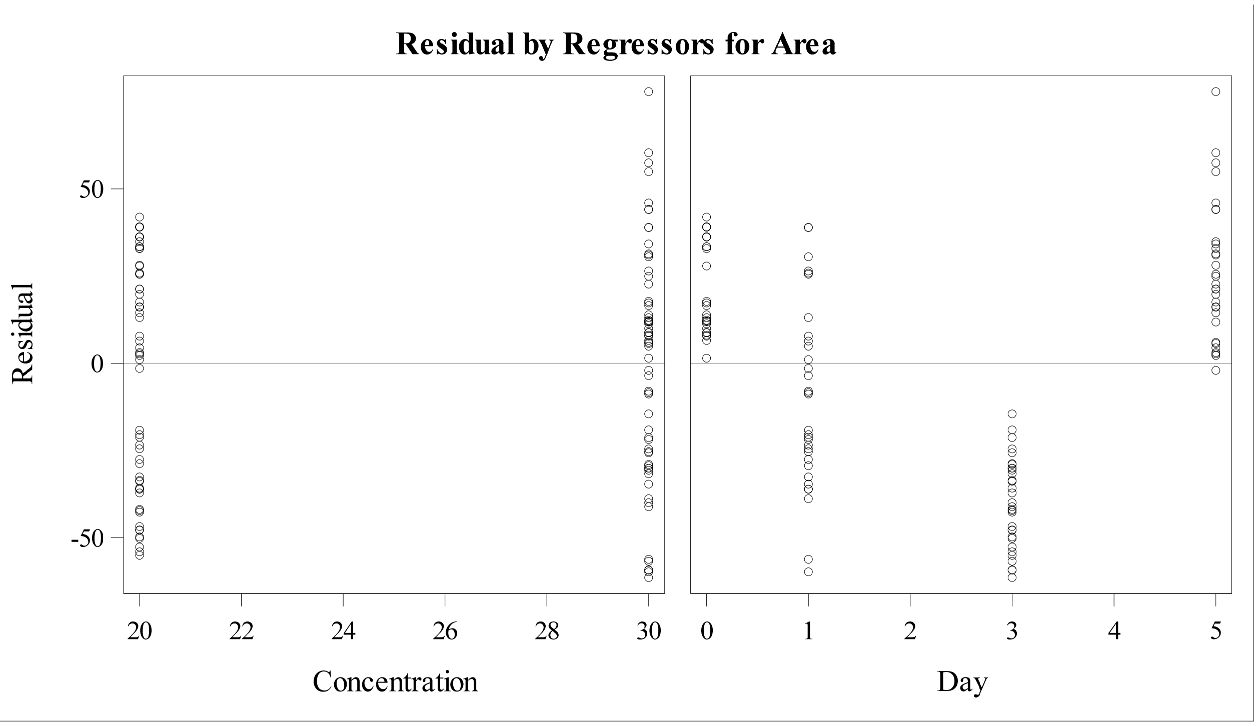

Additional evaluations
Differentaiation medium SMC
Linear Regression of Thickness by Concentration, Day, Print Height and Cultivation

Model: MODEL1
Dependent Variable: Thickness Thickness

**Number of Observations Read**	128
**Number of Observations Used**	128

Stepwise Selection: Step 1

Variable Print_height Entered: R-Square = 0.7065 and C(p) = 159.9157

Analysis of Variance					
**Source**	**DF**	**Sum of Squares**	**Mean Square**	**F Value**	**Pr > F**
**Model**	1	4.73935	4.73935	303.33	<.0001
**Error**	126	1.96870	0.01562		
**Corrected Total**	127	6.70806			

Variable	Parameter Estimate	Standard Error	Type II SS	F Value	Pr > F
Intercept	0.02125	0.05634	0.00222	0.14	0.7067
Print_height	0.96211	0.05524	4.73935	303.33	<.0001

Bounds on condition number: 1, 1

Stepwise Selection: Step 2

Variable Cultivation_thickness__static__1 Entered: R-Square = 0.8576 and C(p) = 15.7497

Analysis of Variance					
Source	DF	Sum of Squares	Mean Square	F Value	Pr > F
Model	2	5.75288	2.87644	376.43	<.0001
Error	125	0.95517	0.00764		
Corrected Total	127	6.70806			

Variable	Parameter Estimate	Standard Error	Type II SS	F Value	Pr > F
Intercept	0.02125	0.03940	0.00222	0.29	0.5906
Print_height	0.96211	0.03863	4.73935	620.22	<.0001
Cultivation_thickness__static__1	-0.08898	0.00773	1.01353	132.64	<.0001

Bounds on condition number: 1, 4

Stepwise Selection: Step 3

Variable Day Entered: R-Square = 0.8727 and C(p) = 3.1714

Analysis of Variance					
Source	DF	Sum of Squares	Mean Square	F Value	Pr > F
Model	3	5.85397	1.95132	283.30	<.0001
Error	124	0.85408	0.00689		
Corrected Total	127	6.70806			

Variable	Parameter Estimate	Standard Error	Type II SS	F Value	Pr > F
**Intercept**	0.05418	0.03838	0.01373	1.99	0.1606
**Day**	-0.01463	0.00382	0.10109	14.68	0.0002
**Print_height**	0.96211	0.03668	4.73935	688.08	<.0001
**Cultivation_thickness__static__1**	-0.08898	0.00734	1.01353	147.15	<.0001

Bounds on condition number: 1, 9

All variables left in the model are significant at the 0.1500 level.

No other variable met the 0.1500 significance level for entry into the model.

Summary of Stepwise Selection									
**Step**	**Variable Entered**	**Variable Removed**	**Label**	**Number Vars In**	**Partial R-Square**	**Model R-Square**	**C(p)**	**F Value**	**Pr > F**
**1**	Print_height		Print_height	1	0.7065	0.7065	159.916	303.33	<.0001
**2**	Cultivation_thickness__static__1		Cultivation	2	0.1511	0.8576	15.7497	132.64	<.0001
**3**	Day		Day	3	0.0151	0.8727	3.1714	14.68	0.0002

Model: MODEL1
Dependent Variable: Thickness Thickness

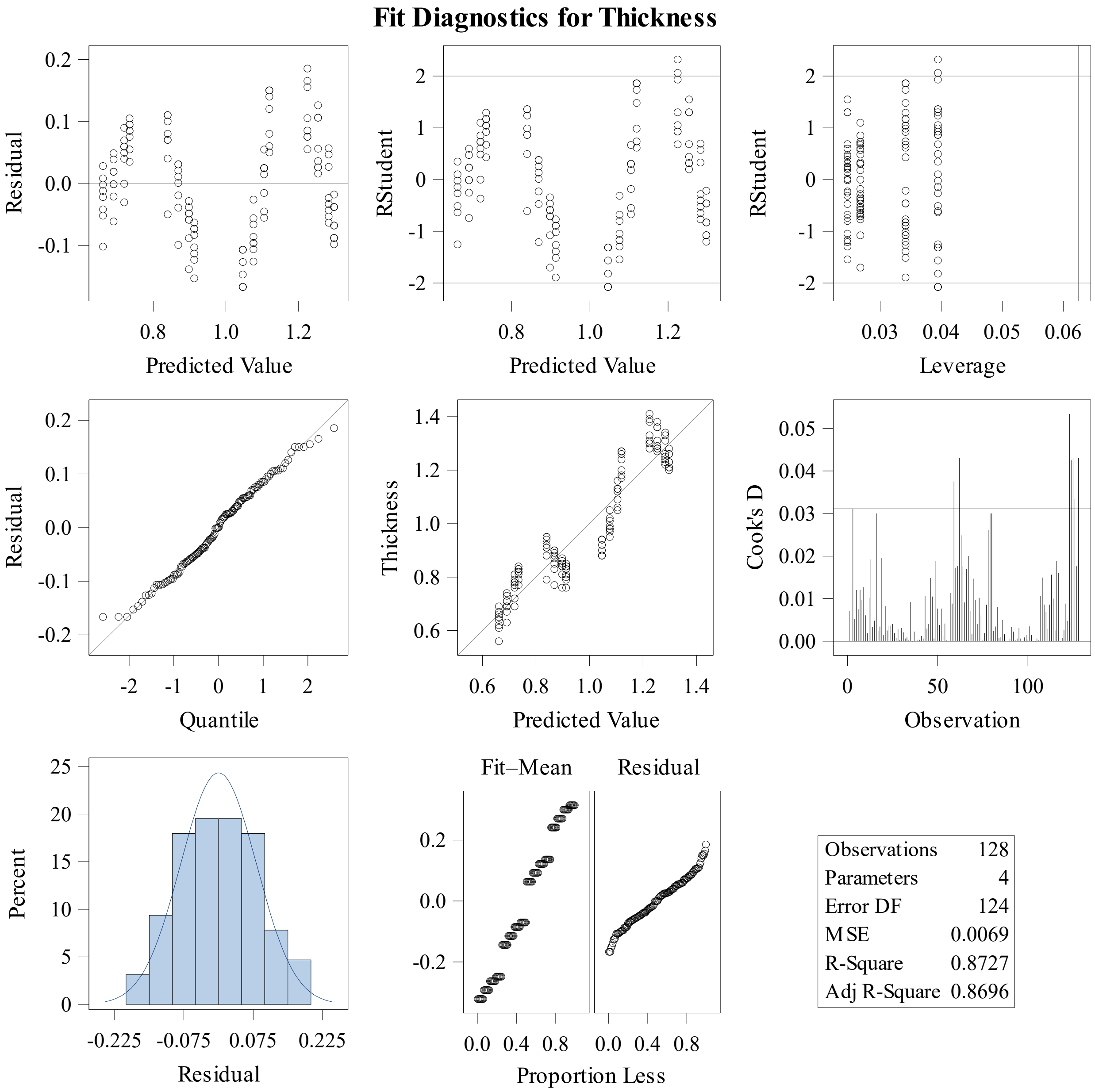
Fit Diagnostics for Thickness
Residual
Predicted Value
RStudent
Predicted Value
RStudent
Leverage
Residual
Quantile
Thickness
Predicted Value
Cook's D
Observation
Percent
Residual
Fit–Mean
Residual
Proportion Less
Observations 128
Parameters 4
Error DF 124
MSE 0.0069
R-Square 0.8727
Adj R-Square 0.8696

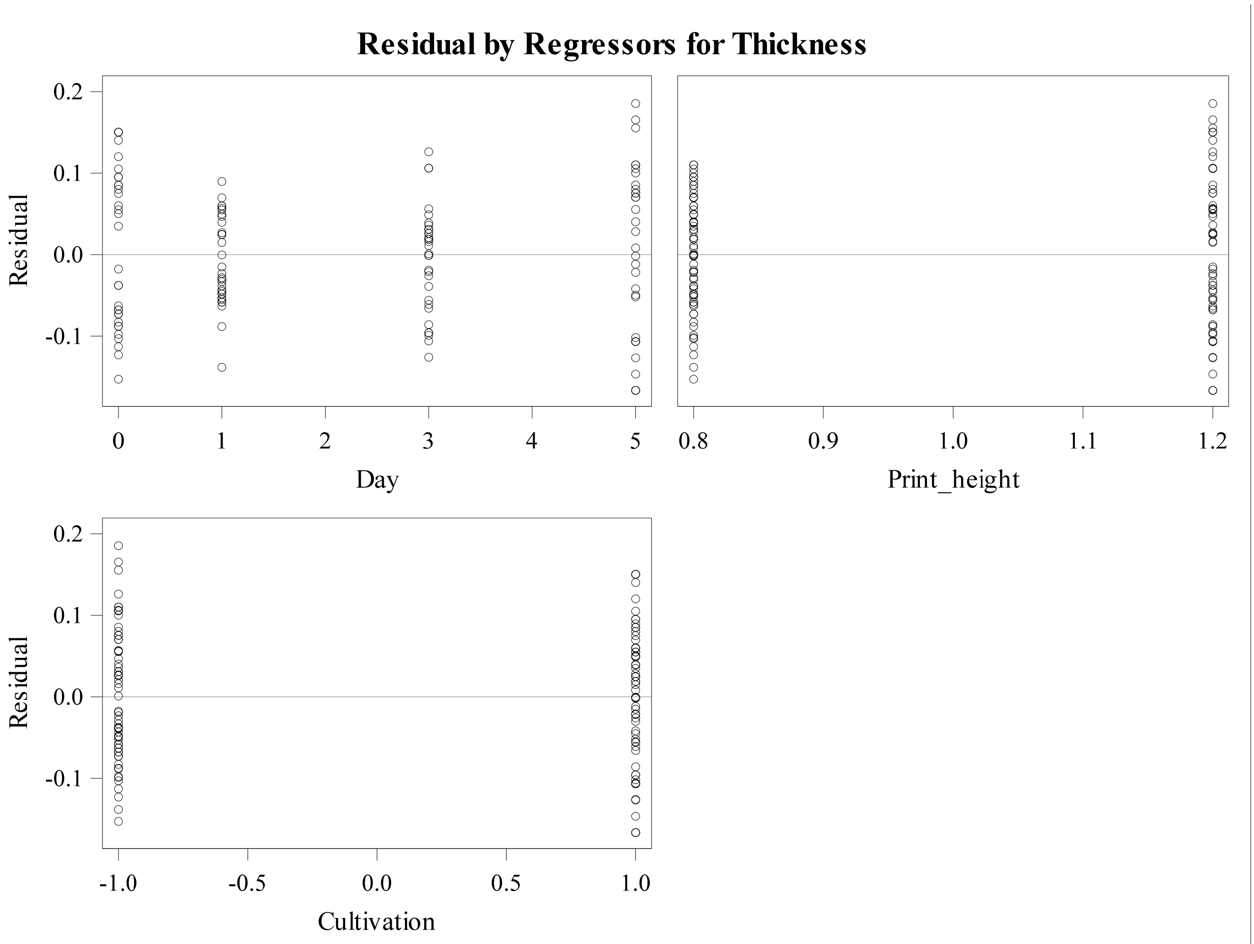
Residual by Regressors for Thickness
Residual
0.2
0.1
0.0
-0.1
0 1 2 3 4 5
Day
0.8 0.9 1.0 1.1 1.2
Print_height
-1.0 -0.5 0.0 0.5 1.0
Cultivation
